# Integrating large language model-generated culturally adapted verbal suggestions into music therapy for university students: A pilot randomized exploratory study

**DOI:** 10.1371/journal.pone.0360478

**Published:** 2026-10-09

**Authors:** Wen Li, Guoqing Wang, Pravina Manoharan, Xuerong Cui, Lu Dai, Li Huang

**Affiliations:** 1 School of Teacher Education, Aba Teachers College, Wenchuan, Sichuan, China; 2 School of the Arts, Universiti Sains Malaysia, Penang, Malaysia; 3 Institute of Higher Music Education, Zhejiang Conservatory of Music, China; Federal University of Paraiba, BRAZIL

## Abstract

Previous research has suggested that culturally mismatched interventions may cause resistance and reduce the effectiveness of music-based interventions. This pilot randomized exploratory study examined the implementation and preliminary outcomes of a music-based intervention incorporating large language model (LLM)-generated, culturally adapted verbal suggestions among Chinese undergraduate students. A total of 113 participants were randomly assigned to three multicomponent conditions: (1) Chinese film music accompanied by LLM-generated verbal suggestions involving Eastern imagery, (2) a standardized music therapy recording with conventional verbal guidance, or (3) piano music without verbal guidance. Following a 40-minute cognitively demanding task, participants completed pre- and post-intervention measures of state anxiety, positive and negative affect, and heart rate. Baseline-adjusted ANCOVAs showed no group differences in positive affect, *F*(2, 109) = 1.03, *p* = .360, *η*_*p*_^2^ = .019, or state anxiety, *F*(2, 109) = 0.68, *p* = .511, *η*_*p*_^2^ = .012. A significant group effect was found for negative affect, *F*(2, 109) = 4.60, *p* = .012, *η*_*p*_^2^ = .078; the Eastern imagery group had lower adjusted post-intervention scores than both comparison groups (Holm-adjusted *ps* = .026). Heart rate also differed across groups, *F*(2, 109) = 10.56, *p* < .001, *η*_*p*_^2^ = .162, with lower adjusted values in the standardized therapy group than in the other two groups. The findings identify potentially meaningful response patterns but do not establish efficacy or the independent effect of culturally adapted verbal suggestions because the conditions differed in musical material and duration. Larger, controlled trials are required. (ClinicalTrials.gov, NCT07359950).

## Introduction

The increasing prevalence of mental health problems among university students has attracted substantial scholarly attention. University students are vulnerable to multiple stressors, including academic pressure, employment concerns, and interpersonal difficulties [1]. In the post-pandemic period, intensified job market competition, fewer internship opportunities, and continuing economic uncertainty have further increased employment-related anxiety among undergraduates [2]. Cross-national studies have shown that academic stress has the strongest negative effect on mental health and subjective well-being, and it may contribute to academic burnout [1,3,4]. Research involving Chinese undergraduates has also found that perceived academic stress significantly increases employment anxiety [2]. Therefore, developing effective stress management strategies and strengthening psychological resilience may help students engage more positively in academic and daily life, thereby improving their overall well-being.

Current interventions for stress reduction primarily encompass cognitive behavioral therapy, mindfulness and meditation practices, and pharmacological treatments for anxiety [5,6]. Although these approaches have demonstrated effectiveness in alleviating anxiety, their application is often constrained by issues such as limited accessibility or potential side effects, particularly among younger populations [7–9]. Therefore, there is a pressing need to investigate alternative stress management strategies that ensure both efficacy and safety.

Music therapy is a non-pharmacological psychological intervention for stress and anxiety reduction [10–15]. It influences emotional experience through listening to music, emotional activation, and guided imagery. Verbal suggestions, as a key component of this approach, may further strengthen emotional engagement [11]. Previous studies have found that music-based interventions not only reduce subjective anxiety but also regulate physiological indicators such as heart rate, producing both psychological and physiological benefits [14–16]. Music therapy is also characterized by its high safety, low cost, and ease of implementation [17]. These features make it particularly suitable for university student populations [18].

In music therapy, verbal suggestions serve as supportive, directive, or reflective tools that strengthen therapist–client interaction and engagement with the musical experience [19–23]. In Chinese cultural context, verbal suggestions translated directly from English often fail to align with local communication norms [24–26]. For example, music therapists commonly use statements such as “You are relaxed” during sessions [27]. However, traditional Chinese culture often values metaphorical, indirect, and context-dependent expressions of emotion [25]. The well-known Chinese proverb “喜怒不形于色” (“Do not reveal joy or anger on one’s face”) is widely regarded as a symbol of wisdom and emotional restraint [28]. Empirical studies suggest that culturally incongruent verbal cues may evoke resistance and reduce the therapeutic effects of music-based interventions [28,29]. However, despite the recognized importance of culturally adapted verbal suggestions, quantitative research on how specific linguistic choices influence intervention outcomes remains limited.

Moreover, existing studies suggest that incorporating local cultural elements into verbal suggestions used in music therapy can enhance recipients’ trust and increase their willingness to follow treatment protocols [5,7,25,29]. Compared with mindfulness exercises, interventions with poetic verbal suggestions produced greater reductions in negative affect [30]. These findings highlight the potential value of integrating culturally grounded language into music-based interventions. However, empirical research on the use of Large Language Models (LLMs) in music therapy remains limited, although LLMs have demonstrated advantages in natural language generation [31,32].

Based on the existing literature, this study aims to compared three intervention conditions, including Group 1: Chinese film music accompanied by LLM-generated Eastern imagery verbal suggestions (music therapy incorporating LLM-generated culturally adapted verbal suggestions), Group 2: a standardized music therapy recording with conventional verbal guidance, and Group 3: piano music without verbal guidance (music only intervention). It was hypothesized that participants receiving music therapy combined with culturally adapted verbal suggestions generated by an LLM would show greater reductions in anxiety levels and heart rate, greater increases in positive affect, and greater decreases in negative affect, compared with participants who received standardized music therapy or music-only intervention.

The remainder of this paper is organized as follows. The next section describes the study design, participants, intervention procedures, and measurement instruments. The following section presents the statistical analysis and results. This is followed by a discussion of the main findings and study limitations. Finally, the paper concludes with a summary of the key insights and directions for future research.

## Methods

In the cultural adaptation literature, experimental evaluation is considered a critical step in assessing the effectiveness of adapted interventions [33]. Accordingly, the present study employed a three-arm pilot randomized design to explore the implementation of LLM-generated culturally adapted verbal suggestions within music therapy and to examine preliminary psychological and physiological response patterns.

### Participants

This study focused on Chinese undergraduate students. A total of 156 undergraduate students were screened for eligibility following recruitment through email invitations at a university in China. Of these, 43 were excluded before randomization: four did not meet the inclusion criteria, and 39 declined participation or withdrew. The remaining 113 participants (19 males and 94 females) were randomly allocated to the three study conditions. Eligible participants were required to be between 18 and 25 years of age. Exclusion criteria included severe hearing impairment and diagnosed cardiovascular conditions. The study protocol was approved by the Institutional Review Board of Universiti Sains Malaysia, Aba Teachers College, registered at ClinicalTrials.gov (NCT07359950). Participants were recruited between January 23 2026 and January 25 2026. All participants provided written informed consent before participation. One of the authors, a music therapist license-holder, administered the music intervention.

### Materials

#### Intervention Group 1: Chinese film music accompanied by LLM-generated Eastern imagery verbal suggestions.

The music used with Intervention Group 1 was drawn from the original soundtrack of the film *Hero* (2002), directed by Zhang Yimou; the music was composed by Tan Dun. The auditory stimulus for the Eastern imagery group was the original soundtrack, the total duration of which was 26 minutes and 19 seconds. It comprised seven musical sections: *Hero Overture*, *For the World*, *Warriors*, *Swift Sword*, *Longing*, *Farewell Hero,* and *Yearning for the Peace*. These selections were chosen for their strong representation of traditional Eastern aesthetic imagery.

The verbal suggestions accompanying the music were generated by ChatGPT 5.2, with a two-step prompt procedure written in Chinese. Prompt 1 (Chinese) was “请生成具有东方意象与文化符号特征的催眠引导语。” (English translation: “Please generate hypnotic suggestive language incorporating Eastern imagery and culturally symbolic elements.”). Prompt 2 (Chinese) was “你知道中国电影《英雄》吗 ? 请基于这部电影的特点, 设计一份大约30分钟的用于音乐治疗的暗示词。” (English translation: “Are you familiar with the Chinese film *Hero*? Based on the characteristics of this film, please design approximately 30 minutes of suggestive language for use in music therapy.”)

The intervention scripts were structured into four sequential therapeutic phases: opening imagery, breath guidance, body sensation, and closing. They are intended to facilitate a distinct affective process: emotional engagement, tension and release, emotional integration, and relaxation [34]. Specifically, the selected musical sections were aligned with these therapeutic-emotional phases: *Hero Overture*, *For the World* (emotional engagement), *Warriors* and *Swift Sword* (tension and release), *Longing* and *Farewell Hero* (emotional integration), and *Yearning for the Peace* (relaxation). In addition, these musical selections exhibit clear musical structures and smooth emotional transitions. Following multiple rounds of discussion between the researchers and certified music therapists, it was determined that embedding verbal suggestions within these sections would not disrupt the listener’s immersive experience [35].

To ensure that the generated suggestions reflected genuine cultural adaptation, the full script underwent multiple rounds of expert review and refinement by the research team, including researchers with backgrounds in Chinese aesthetics and a licensed music therapist. A comparison between standardized music therapy verbal suggestions from *Introduction to Music Therapy* by Professor Gao [35] and LLM-generated culturally adapted suggestions is presented in Table 1. The final script is provided in S1 Appendix.

**Table 1:** Examples of standardized music therapy verbal suggestions and LLM-generated culturally adapted eastern imagery suggestions across intervention phases.

Aspect	Standardized music therapy verbal suggestions	LLM-generated culturally adapted eastern imagery suggestions
Opening imagery *(emotional engagement)*	Imagine a glowing, rotating sphere in front of you…	The wind drifts gently from the east. You hear the distant sound of a *qin*.
Breath guidance *(tension and release)*	With each breath, your body becomes more and more relaxed…	Each breath echoes through the universe.
Body sensation *(emotional integration)*	The sphere shines on your forehead, gently warming it…	Imagine a soft light descending, gathering at your chest, slowly warming...
Closing *(relaxation)*	You are now deeply relaxed...	Mountains and rivers lie in stillness. In this moment, all things are with you.

#### Intervention Group 2: a standardized music therapy recording with conventional verbal guidance.

The music used with Intervention Group 2 was selected from therapeutic recordings presented in *Introduction to Music Therapy* by Professor Tian Gao, a leading music therapy scholar in China. This book provides standardized music therapy protocols with corresponding verbal suggestions. The recording lasted 29 minutes and 56 seconds. Its verbal guidance followed a conventional therapeutic structure, including opening imagery, breath guidance, body awareness, and closing guidance (see Table 1). The music was soft and accompanied by spoken guidance. The suggestions used neutral language and included no specific cultural references.

#### Intervention Group 3: piano music without verbal guidance.

This group listened to a selection of instrumental tracks composed by Japanese musician Joe Hisaishi for the film *Kikujiro* (1999). The playlist included eight pieces: *Summer, Going Out, Mad Summer, The Rain, Kindness, Mother, Two Hearts,* and *Summer Road*. The music was selected according to three criteria. First, all the tracks needed to be instrumental and contain no lyrics, thus reducing potential verbal interference. Second, the pieces had to have a moderate tempo and an accessible melodic structure. Third, the playlist had to provide a gradual emotional transition from mild activation to relaxation. The total duration of the soundtrack was 25 minutes 35 seconds. The sequence followed a structured progression from mild arousal to relaxation, consistent with established principles of emotional regulation in music-based interventions [36]. No verbal suggestions or guided instructions were provided during playback. Emotional arousal and regulation were facilitated solely through music.

### Procedure

The study procedure is presented in Fig 1. Eligible participants were randomly assigned to one of the three groups using a computer-generated simple randomization method (Group 1: n = 36; Group 2: n = 38; Group 3: n = 39). All 113 randomized participants completed the baseline assessment, assigned listening task, and post-intervention assessment. No participants were lost to follow-up after randomization. Participants were not informed of the specific study hypotheses or the differences between the groups. They were also not told whether the verbal suggestions were generated by an LLM or based on conventional therapeutic scripts. We intentionally paired culturally similar musical materials with the corresponding verbal suggestions to enhance the cultural consistency of the intervention content. The study protocol and CONSORT checklist are provided in S2 and S3 Appendix. The experiment was conducted in a quiet multimedia room at the university. To reduce potential therapist-related bias, the same therapist conducted all the intervention sessions. Procedural interactions were standardized across groups.

**Fig 1:**
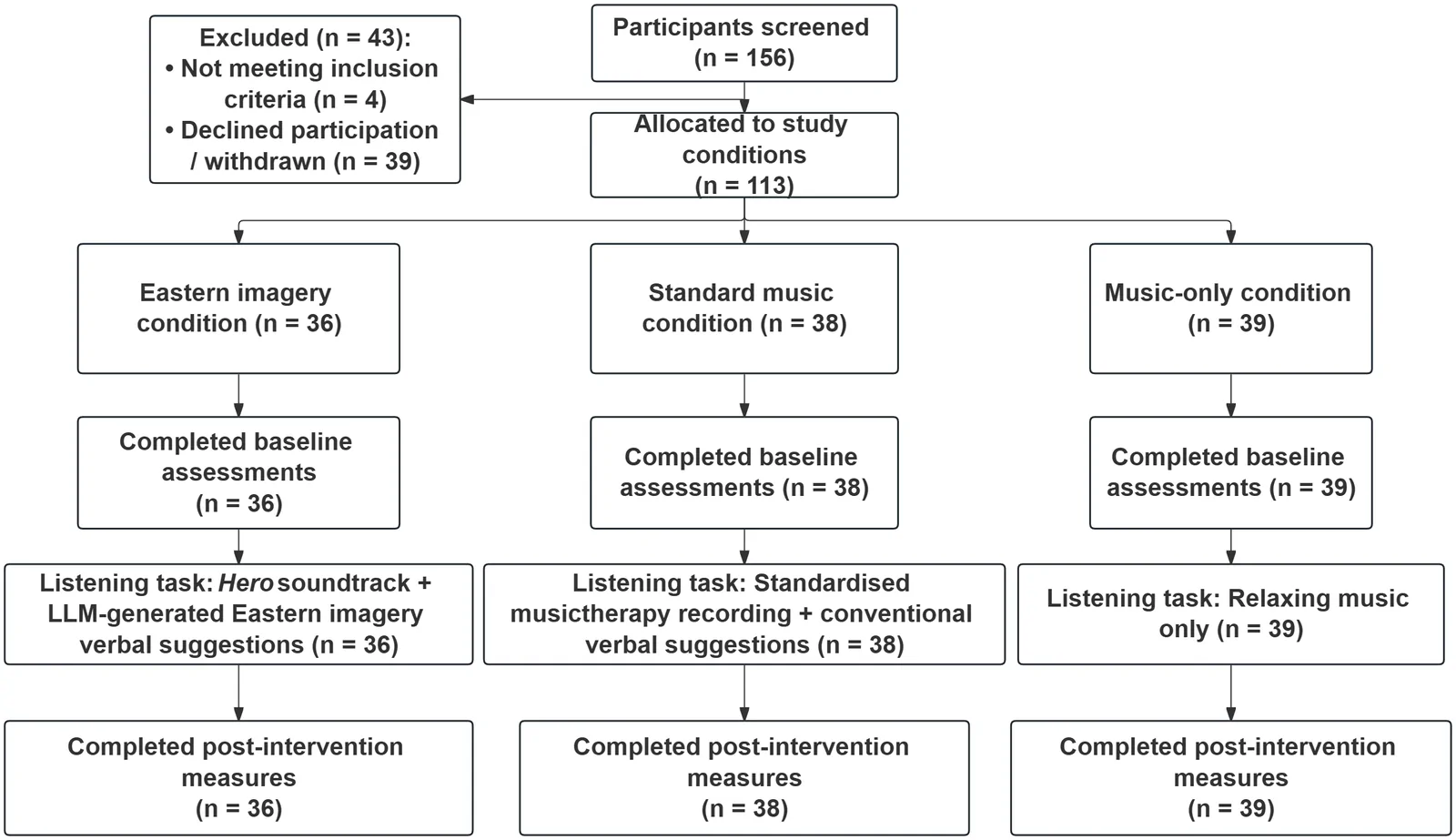
CONSORT flowchart.

After providing written informed consent, the participants completed a 40-minute time-constrained cognitive task. They were required to organize course-related content into a structured mind map. The task was intended to create a cognitively demanding academic context involving information organization. Previous research has shown that high cognitive load under time pressure can effectively induce stress responses in experimental settings [36,37].

Immediately after the stress induction phase, participants completed the pre-test via the online survey platform Wenjuanxing. Therefore, these assessments served as a post-induction baseline, capturing the psychological and physiological states of the participants after stress induction but prior to the music intervention. The measures included demographic information (gender, age), the State-Trait Anxiety Inventory (STAI), the Positive and Negative Affect Schedule (PANAS), and heart rate recordings as objective physiological indicators. Participants received the music intervention after the survey. They were instructed to sit comfortably with their eyes closed, concentrating exclusively on the music without undertaking any additional tasks. After the intervention, a post-test was conducted using the same instruments. At the end of the study, participants received a full debriefing and were compensated with a gift valued at 20 Chinese Yuan.

### Measurement instruments

The STAI and PANAS are widely used self-report scales. They enable efficient assessment without labor-intensive scoring or interpretation [38–41]. These measures mainly record subjective emotional responses. Heart rate was also included in this study as an objective physiological indicator of arousal.

#### State–Trait Anxiety Inventory (STAI).

Participants’ anxiety levels were measured using the STAI, developed by Spielberger et al. [38]. This internationally validated tool demonstrates strong reliability and validity [39]. Previous studies also have confirmed the applicability and psychometric soundness of the STAI among Chinese university students [40]. It includes two subscales: State Anxiety (STAI-S) and Trait Anxiety (STAI-T).

The STAI-S assesses transient anxiety experienced in specific situations, and it is suitable for pre-post comparisons in short-term interventions. In contrast, the STAI-T evaluates stable, long-term anxiety predispositions. Each subscale comprises 20 items rated on a four-point Likert scale, with higher scores indicating greater anxiety levels. In this study, the STAI-S demonstrated high internal consistency, with Cronbach’s α coefficients of 0.85 (pre-test) and 0.84 (post-test). The STAI-T was collected only at pre-test. Its Cronbach’s α coefficient was 0.85, indicating good internal reliability.

#### Positive and Negative Affect Schedule (PANAS).

The PANAS was used to assess emotional experience [41]. A standardized and extensively used tool in emotion research, the PANAS can capture short-term emotional fluctuations. It includes two independent subscales: positive affect and negative affect, each comprising 10 items rated on a five-point Likert scale. Item-mean scores were calculated for each PANAS subscale. Higher PA scores reflect greater positive emotional experiences, whereas higher NA scores indicate more intense negative emotional states.

In this study, the PA subscale demonstrated excellent internal consistency, with Cronbach’s α values of 0.93 (pre-test) and 0.95 (post-test). The NA subscale also showed strong reliability, with Cronbach’s α values of 0.87 (pre-test) and 0.90 (post-test). These results confirm the robust measurement properties of both PANAS subscales in the sample used in this study.

### Heart rate

This study also included heart rate as an objective physiological indicator of arousal. To obtain objective observational data, the heart rate changes of the participants were measured using the photoplethysmography (PPG) technology embedded in mobile phones. PPG uses a smartphone camera and flash to detect pulsatile changes in fingertip blood flow and estimate heart rate [42]. Specifically, participants were instructed to use the “JingDong Health” mobile application to record their heart rates. In previous study, the mobile-based method has demonstrated reliability for resting heart rate measurements in academic research [43]. Heart rate was measured before and immediately after the intervention, with each recording lasting approximately one minute. Participants were instructed to remain seated in a relaxed position, minimize movement, and maintain stable finger placement on the camera sensor during measurement.

Heart rate data were collected twice: a pre-intervention baseline and a post-intervention recovery period. Each measurement lasted for approximately one minute. Heart rates were measured immediately after the intervention to capture the immediate physiological response. All the measurements were conducted under standardized environmental conditions, including controlled room temperature, lighting, and noise levels, to minimize any potential external influences on the HR recordings.

### Post-hoc exploratory assessment of perceived cultural resonance

To explore the participants’ perceptions of the cultural relevance of the Eastern imagery verbal suggestions, a post-hoc exploratory assessment was conducted after the intervention in the culturally framed condition. Participants rated two items on seven-point Likert scales: (1) “To what extent did the verbal suggestions feel culturally relevant to Chinese culture?” and (2) “To what extent did the verbal suggestions feel personally resonant and meaningful to you?” (1 = *not at all*, 7 = *extremely*). Since this assessment was not administered to the comparison groups, it was not treated as a formal manipulation check.

### Data analysis

All the statistical analyses were conducted using IBM SPSS Statistics version 28.0 and SPSSAU, an intelligent online statistical analysis platform. Primary between-group analyses were conducted using analysis of covariance (ANCOVA). For each outcome—state anxiety, positive affect, negative affect, and heart rate—the post-intervention score was entered as the dependent variable, randomized group as a fixed factor, and the pre-intervention score as the baseline covariate. The homogeneity-of-regression-slopes assumption was evaluated by testing the group × baseline interaction. Furthermore, the raw heart-rate data in the present study were not normally distributed. Residual normality and homogeneity of residual variance were assessed using the Shapiro–Wilk and Levene tests, respectively. Heart rate is regulated by the autonomic nervous system, and cardiac responses may vary across physiological and experimental contexts [44,45]. Because the normality assumption of ANCOVA primarily concerns the model residuals rather than the raw distribution of the dependent variable, the suitability of the heart-rate ANCOVA model was evaluated by examining the normality of its residuals. Omnibus group effects are reported with partial eta squared, and adjusted group means are presented with 95% confidence intervals. When the omnibus group effect was statistically significant, pairwise comparisons of adjusted means were performed with Holm correction across the three contrasts within each outcome. Potential model outliers were defined as observations with an absolute externally studentized residual greater than 3. All valid observations were retained in the primary analyses. For outcomes containing observations that met this criterion, the ANCOVA was repeated after their exclusion as a sensitivity analysis. For outcomes with no identified model outliers, an outlier-excluded sensitivity analysis was not applicable. Inferential robustness was further examined using HC3 heteroskedasticity-consistent standard errors. All tests were two-sided, with α = .05 [46].

## Results

The analytic sample comprised 113 participants: 36 in Group 1 (music therapy incorporating LLM-generated culturally adapted eastern imagery verbal suggestions), 38 in Group 2 (standardized music therapy group), and 39 in Group 3 (music-only relaxation group). Data for positive affect, negative affect, state anxiety, and heart rate were complete within this analytic sample. The observed pre-intervention (baseline) and post-intervention means and standard deviations are presented in Table 2. These unadjusted descriptive values are provided to characterize the outcome distributions; comparative inferences were based on the baseline-adjusted models reported below. The group × baseline interactions were not statistically significant in any of the complete-data models (*ps* = .113–.709), supporting the homogeneity-of-regression-slopes assumption. Levene tests were also non-significant (*ps* = .073–.405). Although residual normality was not supported for the three self-report outcomes, the HC3 robust analyses yielded the same inferential conclusions as the conventional ANCOVAs.

**Table 2:** Observed pre-intervention and post-intervention outcome scores by group.

Outcome	Group	n	Pre-intervention M (SD)	Post-intervention M (SD)
Positive affect	Eastern imagery	36	2.72 (0.92)	2.60 (1.11)
	Standard music therapy	38	2.89 (0.84)	2.92 (1.05)
	Music only	39	2.51 (0.75)	2.63 (0.83)
Negative affect	Eastern imagery	36	2.03 (0.61)	1.57 (0.66)
	Standard music therapy	38	2.42 (0.64)	1.99 (0.72)
	Music only	39	2.19 (0.84)	1.97 (0.58)
State anxiety	Eastern imagery	36	44.39 (5.80)	41.61 (4.85)
	Standard music therapy	38	44.89 (7.12)	43.34 (8.13)
	Music only	39	44.03 (7.37)	43.00 (6.58)
Heart rate (bpm)	Eastern imagery	36	82.81 (5.54)	78.61 (4.72)
	Standard music therapy	38	81.11 (6.01)	74.95 (4.39)
	Music only	39	81.92 (5.13)	79.03 (3.85)

### Primary ANCOVA results

Table 3 summarizes the ANCOVA results and baseline-adjusted post-intervention means. There was no statistically significant group effect on positive affect, *F*(2, 109) = 1.03, *p* = .360, *η*_*p*_*²* = .019. The adjusted means were 2.60 (95% CI [2.27, 2.93]) for the Eastern imagery group, 2.91 (95% CI [2.58, 3.23]) for the standard music therapy group, and 2.64 (95% CI [2.32, 2.96]) for the music-only group.

**Table 3:** Primary ANCOVA results and baseline-adjusted post-intervention means.

Outcome	Eastern imageryAdj. M [95% CI]	Standard therapyAdj. M [95% CI]	Music onlyAdj. M [95% CI]	*F* (df1, df2)	*p*	*η*_*p*_ *²*
Positive affect	2.60 [2.27, 2.93]	2.91 [2.58, 3.23]	2.64 [2.32, 2.96]	1.03 (2, 109)	.360	.019
Negative affect	1.57 [1.35, 1.79]	1.99 [1.78, 2.20]	1.97 [1.76, 2.18]	4.60 (2, 109)	.012	.078
State anxiety	41.62 [39.42, 43.81]	43.28 [41.14, 45.42]	43.06 [40.94, 45.17]	0.68 (2, 109)	.511	.012
Heart rate (bpm)	78.27 [77.02, 79.52]	75.27 [74.05, 76.48]	79.03 [77.83, 80.22]	10.56 (2, 109)	< .001	.162

*Note.* Adjusted means were estimated at the overall mean of the corresponding baseline outcome. All primary ANCOVA models included the complete analytic sample (*N* = 113). Adj. M = adjusted mean; bpm = beats per minute; CI = confidence interval; *ηₚ²* = partial eta squared.

A statistically significant group effect was observed for negative affect, *F*(2, 109) = 4.60, *p* = .012, *η*_*p*_*²* = .078. Holm-adjusted comparisons indicated that the Eastern imagery group had a lower adjusted post-intervention negative-affect score than both the standard music therapy group (mean difference = −0.42, 95% CI [−0.73, −0.11], *p* = .026) and the music-only group (mean difference = −0.40, 95% CI [−0.70, −0.10], *p* = .026), see Table 4. The standard music therapy and music-only groups did not differ (*p* = .893).

**Table 4:** Holm-adjusted pairwise comparisons for outcomes with a significant omnibus group effect.

Outcome	Contrast	Mean difference	95% CI	*t* (df)	Holm *p*
Negative affect	Eastern imagery − Standard therapy	−0.42	[−0.73, −0.11]	−2.68 (109)	.026
	Eastern imagery − Music only	−0.40	[−0.70, −0.10]	−2.62 (109)	.026
	Standard therapy − Music only	0.02	[−0.28, 0.32]	0.13 (109)	.893
Heart rate	Eastern imagery − Standard therapy	3.01	[1.26, 4.76]	3.41 (109)	.002
	Eastern imagery − Music only	−0.76	[−2.48, 0.97]	−0.87 (109)	.388
	Standard therapy − Music only	−3.76	[−5.47, −2.06]	−4.37 (109)	< .001

*Note.* Contrasts are based on baseline-adjusted means. Confidence intervals are model-based, unadjusted 95% intervals; *p* values were adjusted across the three pairwise comparisons for each outcome using Holm’s procedure.

In the complete-data state-anxiety analysis (N = 113), the adjusted group effect was not statistically significant, *F*(2, 109) = 0.68, *p* = .511, *ηₚ²* = .012. The adjusted means were 41.62 (95% CI [39.42, 43.81]) for the Eastern imagery group, 43.28 (95% CI [41.14, 45.42]) for the standard music therapy group, and 43.06 (95% CI [40.94, 45.17]) for the music-only group.

The baseline-adjusted group effect on post-intervention heart rate was statistically significant, *F*(2, 109) = 10.56, *p* < .001, *η*_*p*_*²* = .162. The standard music therapy group had a lower adjusted heart rate than both the Eastern imagery group (mean difference = −3.01 bpm, 95% CI [−4.76, −1.26], Holm-adjusted *p* = .002) and the music-only group (mean difference = −3.76 bpm, 95% CI [−5.47, −2.06], Holm-adjusted *p* < .001). The Eastern imagery and music-only groups did not differ (*p* = .388).

### Post-hoc exploratory assessment of perceived cultural resonance

Following the intervention, participants in Group 1 completed a brief exploratory assessment regarding their perceptions of the cultural relevance of the Eastern imagery verbal suggestions. Participants rated the suggestions as culturally relevant (M = 5.03, SD = 1.18) and personally resonant (M = 5.23, SD = 1.03). Both ratings were significantly higher than the neutral midpoint of the scale (both *p* < .001). These findings provided preliminary descriptive evidence that participants perceived the suggestions as culturally relevant and personally resonant.

### Sensitivity and Robustness Analyses

Two observations in the state-anxiety model met the criterion for potential model outliers. Excluding these observations in a sensitivity analysis (*N* = 111) did not change the substantive conclusion: the group effect remained non-significant, *F*(2, 107) = 1.40, *p* = .252, *ηₚ²* = .025. The corresponding HC3 robust test was also non-significant, *F*(2, 107) = 1.40, *p* = .251. However, the group × baseline interaction was statistically significant in the reduced dataset, *F*(2, 105) = 4.86, *p* = .010. Therefore, the common-slope estimates from this sensitivity analysis should be interpreted cautiously. No potential model outliers were identified for positive affect, negative affect, or heart rate; consequently, no outlier-excluded sensitivity analyses were applicable to these outcomes.

HC3 robust tests of the complete-data primary models yielded the same inferential conclusions as the conventional ANCOVAs. Group effects were non-significant for positive affect, *F*(2, 109) = 0.91, *p* = .406, and state anxiety, *F*(2, 109) = 0.83, *p* = .440, but remained significant for negative affect, *F*(2, 109) = 4.50, *p* = .013, and heart rate, *F*(2, 109) = 10.23*, p* < .001.

### Effect of the eastern imagery group by trait anxiety levels

To evaluate whether the intervention effect of the Eastern imagery group was influenced by the trait anxiety levels of the participants, the latter were categorized into high and low trait anxiety groups. In research contexts, a cutoff score of 40 or higher on the STAI-T is employed to signify clinically significant anxiety levels [13,43]. Given the unequal sample sizes between the high (n = 29) and low trait anxiety groups (n = 7), Welch’s t-tests were utilized to assess group differences in the changes in positive and negative affect, as well as the STAI scores. None of these comparisons achieved statistical significance (*p* > .05).

The Mann–Whitney U test was used to examine group differences in heart rate changes. The results indicated no significant difference between the two groups (U = 118.0, *p* = .52). The intervention effect of the Eastern imagery group did not vary significantly based on the participants’ anxiety levels. Given the relatively small sample size of the low trait anxiety group (n = 7), the results related to this subgroup should be interpreted with caution.

## Discussion

This pilot randomized exploratory study examined the integration of LLM-generated, culturally adapted verbal suggestions into a single-session music-based intervention. Four findings are central. First, no baseline-adjusted between-group differences were detected for state anxiety or positive affect. Second, negative affect differed across conditions after baseline adjustment, with the Eastern imagery group showing lower adjusted post-intervention scores than both comparison groups. Third, heart rate differed across conditions, but the lowest adjusted post-intervention values were observed in the standardized music therapy group rather than the Eastern imagery group. Fourth, participants exposed to the Eastern imagery suggestions rated them as culturally relevant and personally resonant. To the best of the authors’ knowledge, few empirical studies have examined how culturally adapted verbal suggestions generated by LLMs can be incorporated into music therapy. This study provides an initial quantitative investigation of this emerging approach and contributes to addressing this gap.

### Anxiety

Contrary to the original hypothesis, the baseline-adjusted state-anxiety scores did not differ significantly among the three groups. Although state anxiety decreased within the Eastern imagery group, the corresponding within-group result cannot establish a comparative intervention effect. The absence of a significant adjusted group effect therefore provides no evidence that the Eastern imagery condition reduced state anxiety more than the standardized therapy or music-only conditions. One possible explanation is that the three groups were exposed to substantially different musical stimuli. Specifically, the Eastern imagery group listened to Chinese film music, the standard verbal suggestion group received a clinical music therapy recording, and the music-only group listened to Japanese film music. Since each condition involved distinct musical materials, the unique contribution of the verbal suggestions perhaps cannot be fully separated from the music itself. Meanwhile, the observed outcome patterns may have been influenced by differences in musical characteristics, verbal content, the participants’ familiarity with the musical materials, or interactions among these factors [47]. Previous research also suggested that cultural relevance may shape individual psychological responses to music-based interventions [48]. Therefore, the heterogeneity of musical materials used in the present study should be considered as a potential factor that affected the interpretation of the observed intervention effects. This may also partially explain the absence of significant differences among groups.

### Negative affect

The baseline-adjusted analysis identified a statistically significant group effect for negative affect. The Eastern imagery group had lower adjusted post-intervention negative-affect scores than both the standardized music therapy and music-only groups. This pattern is consistent with the idea that culturally familiar, imagery-rich language may facilitate engagement [10,14,19,21,22]. Nevertheless, the result cannot be attributed specifically to cultural adaptation or LLM generation. The Eastern imagery condition combined a distinctive musical repertoire with culturally framed language, whereas the comparison conditions differed on several components. The finding should therefore be regarded as a possible signal warranting confirmatory testing.

### Positive affect

By contrast, neither the baseline-adjusted analysis nor the within-group analyses identified a significant effect on positive affect. The absence of significant changes in positive affect is unlikely to be explained by ceiling or floor effects, as the baseline scores were within a moderate range across all the groups. Instead, this finding is consistent with previous research indicating that music-based interventions are generally more effective in reducing negative affect than in enhancing positive affect [49]. Similarly, experimental evidence has shown that music listening can effectively regulate negative emotional states following stress induction [50]. This asymmetry may reflect the primary role of music as an emotion regulation tool, particularly in reducing stress and modulating physiological arousal, rather than directly enhancing positive emotional states. Furthermore, prior research suggests that increases in positive affect may require more active forms of engagement, such as music performance or social interaction [47], whereas passive listening primarily facilitates emotional stabilization [51,52].

### Heart rate

Heart rate decreased within all three conditions, and the baseline-adjusted ANCOVA identified a significant group effect. The standardized music therapy group showed lower adjusted post-intervention heart rate than both the Eastern imagery and music-only groups, whereas the latter two groups did not differ. All three groups exhibited heart rate reductions after the intervention, suggesting that music interventions can effectively modulate physiological arousal [53,54]. However, between-group comparisons indicated that the standard music therapy group achieved the most substantial heart rate reduction. This outcome may be attributed to the structured components of standard therapy protocols, including guided breathing and sequenced procedures, which are known to enhance parasympathetic activation and diminish autonomic tension [55,56].

### Trait anxiety

The exploratory subgroup analyses did not identify statistically significant differences between participants with higher and lower trait anxiety. This null finding should not be interpreted as evidence that the intervention is broadly applicable across trait-anxiety levels. Only seven participants were included in the lower-trait-anxiety subgroup, producing substantial imprecision and limited statistical power. The subgroup findings are therefore descriptive and require examination in a larger sample with prospectively defined strata or a continuous moderation analysis.

### Cultural resonance and the role of the LLM

Participants in the Eastern imagery condition rated the verbal suggestions as culturally relevant (*M* = 5.03, *SD* = 1.18) and personally resonant (*M* = 5.23, *SD* = 1.03) on seven-point scales. These ratings provide descriptive evidence that the generated material was perceived positively by the group that received it. The practical contribution of the LLM in this study was its capacity to generate an initial set of culturally adapted verbal suggestions, which was subsequently reviewed and refined by researchers with expertise in music therapy. This human review remains essential because cultural appropriateness, clinical safety, and therapeutic fit cannot be assumed from model output alone.

### Limitations and future research

This study has several limitations that should be considered for future research. First, although the final sample size was adequate to detect moderate effects, it may not be sufficient to identify small between-group differences. Moreover, the sample was predominantly female, which may limit the generalizability of the findings. Future studies should recruit larger and more gender-balanced samples to enhance the statistical power and improve the representativeness of the study population. Second, it is acknowledged that the use of different musical materials across experimental conditions represents a limitation of the study design. Future studies should employ identical musical materials across conditions while manipulating only the verbal suggestion component. In this way, it may reduce potential confounding effects and enabling a clearer evaluation of the unique contribution of culturally adapted verbal suggestions. Third, this study employed a stress induction task; however, the effectiveness of the manipulation was not directly verified. Future studies should include pre- and post-task assessments of state anxiety to evaluate whether a high-load cognitive task successfully induces immediate anxiety responses. Such assessments would enhance internal validity. Meanwhile, future studies should administer STAI-T prior to high-load cognitive tasks to minimize potential task-related confounding effects on trait anxiety measurement. Further research is needed to explore how to generate and optimize culturally customized prompts with rich aesthetic imagery via LLMs.

## Conclusion

This pilot randomized exploratory study indicates that LLM-generated culturally adapted verbal suggestions can be incorporated into a structured music-based intervention. Participants in the Eastern imagery condition rated the suggestions as culturally relevant and personally resonant. Baseline-adjusted analyses identified between-group differences in negative affect and heart rate, but not in state anxiety or positive affect. These findings should be interpreted with caution: the intervention conditions differed in musical material and duration, so the independent effects of the LLM-generated, culturally adapted suggestions could not be isolated. Future research should examine the effectiveness of LLM-assisted music therapy with larger samples and explore its potential applications in other areas, such as sleep disturbance and subclinical anxiety.

## Supporting information

S1 Appendix Full script of the *Hero* music therapy suggestion language (Eastern imagery version). (PDF)

S2 Appendix Study protocol. (PDF)

S3 Appendix CONSORT checklist. (PDF)
