# Supplementary material for: Integrating large language model-generated culturally adapted verbal suggestions into music therapy for university students: A pilot randomized exploratory study
Source: PLoS One. 2026 Oct 9;21(10):e0360478. doi: 10.1371/journal.pone.0360478 (PMC13652252; doi:10.1371/journal.pone.0360478)
Supplement: Supplementary file 1 [file pone.0360478.s001.pdf]

S1 Appendix A

"Hero" Music Therapy Suggestion Language (Eastern Imagery Version). This script is designed for use in a 25-minute live music therapy session. Each verbal suggestion corresponds to a specific musical track and should be delivered in sync with the music.The recommended speaking pace is approximately 80–100 words per minute, with a 2–3 second pause between sentences to allow for silent reflection.

🎵 1. ***For the World – Awakening of Light.*** *Theme: Awareness and Inner Awakening*

The wind rises slowly from the East. You hear the faint sound of a zither in the distance. Each breath you take echoes through heaven and earth. Imagine a gentle light softly descending, gathering at your chest, gradually warming you, making you feel accepted and protected. The mountains and rivers fall silent. You simply breathe—In this moment, all things are with you.

🪶 *Psychological mechanism*: Establishes a sense of safety → Induces alpha brain waves → Emotional stabilization🧠 *Eastern symbolism*: "Light" represents enlightenment and self-awareness; "Breath" symbolizes the origin of life.

🎵 2. ***Warriors – The Awakening of Strength and Heart.*** *Theme: Inner Strength and the Balance of Conflict*

The drums resound…You feel power flowing through your body. Each beat of the drumshatters a fixation within your heart. You begin to see—True strength lies not in conquest, but in unwavering perseverance. Between movement and stillness, you discover what is eternal.

🪶 *Psychological mechanism*: Emotional release → Stress reduction → Restoration of inner strength🧠 *Eastern symbolism*: Fire and sword represent the balance of "courage" and "wisdom"; embodying the philosophy of "ending conflict to achieve peace."

🎵 3. ***Swift Sword – Blade in the Water.*** *Theme: Letting Go and Flowing with Ease*

Water flows. It asks not about the height of the mountain, nor the depth of the valley. Imagine a clear stream cascading from your forehead, flowing past your shoulders and fingertips, carrying away all tension and fixation .In this moment, you are the water. When the wind rises, you move with it; When mountains appear, you flow around them.

🪶 *Psychological mechanism*: Release of negative emotions → Physical relaxation (synchronized breathing and music) 🧠 *Eastern symbolism*: Water reflects Daoist wisdom—"gentleness overcomes strength"; representing emotional dissolution and adaptability.

🎵 4. ***Yearning for the Peace – Harmony Within.*** *Theme: Tranquility and Integration*

The wind has ceased. The dust has settled. Between heaven and earth, only quiet breath remains. This breath may come from a distant mountain, or perhaps from deep within your heart—gentle, yet full of strength. In the distance, a river flows. The surface is still, like a mirror. You approach, and your reflection appears. No sorrow, no joy. You reach out your hand and gently touch the water. Ripples spread outward in soft circles. You watch as anxiety, regret, and attachment dissolve into the water, becoming transparent. Let the music continue to flow. When the final note fades away, slowly open your eyes. You remain where you are—but the world feels softer, and your heart, clearer.

🪶 *Psychological mechanism*: Integration → Self-acceptance → Meaning construction 🧠 *Eastern symbolism*: Wind and plains symbolize "harmony through non-action"; unity of heart and world.
