# Supplementary material for: Integrating large language model-generated culturally adapted verbal suggestions into music therapy for university students: A pilot randomized exploratory study
Source: PLoS One. 2026 Oct 9;21(10):e0360478. doi: 10.1371/journal.pone.0360478 (PMC13652252; doi:10.1371/journal.pone.0360478)
Supplement: Supplementary file 2 [file pone.0360478.s002.pdf]

STUDY PROTOCOL

Project Title: Effects of AI Large Language Model-Assisted Film Music Therapy on the Mental Health of University Students

1. Research Background

In recent years, mental health problems among university students have attracted extensive attention. University students commonly face multiple stressors, including academic pressure, employment concerns, and interpersonal difficulties. These factors may adversely affect mental health and subjective well-being and may further increase the risks of academic burnout and anxiety (Sheldon et al., 2021; Córdova Olivera et al., 2023). In particular, against the background of intensified employment competition and economic uncertainty, perceived academic stress among university students may further increase employment-related anxiety. Therefore, it is important to explore safe, effective, and easily implemented methods for emotion and stress regulation (Yang et al., 2025).

Current interventions for stress and anxiety commonly include cognitive behavioral therapy, mindfulness and meditation training, and pharmacological treatment. Although these approaches can alleviate anxiety symptoms to some extent, their application may still be constrained by accessibility, implementation conditions, or medication-related side effects (Demir & Ercan, 2022; Dawson et al., 2020; Strawn et al., 2018). In contrast, music therapy is a non-pharmacological psychological intervention characterized by relatively high safety, low implementation cost, and good feasibility. Existing systematic reviews and meta-analyses indicate that music interventions can reduce anxiety and stress and may also regulate physiological indicators such as heart rate, making them particularly suitable for non-clinical populations such as university students (de Witte et al., 2022; Harney et al., 2022).

During music therapy, verbal suggestions can serve as supportive, directive, or reflective tools to help participants enter the musical context, regulate attention, and strengthen emotional experience (Amir, 1999; Nolan, 2005). However, verbal suggestions have clear cultural attributes. Directly translating verbal expressions used in Western music therapy and applying them to Chinese participants may not fully align with the relatively implicit, indirect, and context-dependent ways of expressing emotion in Chinese culture (Guo et al., 2024). Existing studies suggest that culturally incongruent verbal cues may evoke psychological resistance, whereas intervention content incorporating local cultural elements may enhance participants' identification with and acceptance of the intervention (Liu et al., 2025; Wu et al., 2021).

In recent years, AI-based Large Language Models (LLMs) have demonstrated strong capabilities in natural language generation, creating new technical possibilities for generating personalized and culturally adapted therapeutic language (Liu et al., 2024). However, empirical research applying LLM-generated culturally adapted verbal suggestions in music therapy remains very limited. Therefore, this study attempts to integrate LLM-generated verbal suggestions based on Eastern cultural imagery into music therapy and compare this approach with standardized verbally guided music therapy and music-only intervention, in order to explore the effects of this novel intervention on psychological and physiological indicators such as anxiety, positive and negative affect, and heart rate among university students.

This study is a sub-study of the project "Film-Based Therapy for Mental Health among Young Adults in China," which began in 2024. The earlier phase of the project focused on exploring the effects of film-based therapy on the mental health of young adults in China. The present study focuses on the application of culturally adapted verbal suggestions generated by AI large language models in music therapy, with the aim

of exploring a mental health intervention model that integrates film music, cultural imagery, and verbal guidance.

2. Research Objectives

1. To investigate the effects of AI large language model-assisted film music therapy on reducing anxiety and negative affect among university students.
2. To examine the effectiveness of AI-assisted film music therapy in improving the mental health of university students.
3. To compare the intervention effects of AI-assisted film music therapy, standard music therapy, and music-only relaxation.
4. To provide a standardized AI-enabled music therapy approach for mental health interventions among university students.

3. Participants

3.1 Inclusion Criteria

- Full-time undergraduate students from a university in China, aged 18-25 years.
- Voluntary participation in the study and provision of written informed consent.
- No severe hearing impairment and able to participate normally in the music intervention and assessments.
- Planned recruitment period: December 2025 to January 2026.

3.2 Exclusion Criteria

- Individuals diagnosed with severe cardiovascular disease or psychiatric disorders.
- Individuals unable to complete the 40-minute stress task and the 30-minute intervention.

3.3 Sample Information

A total of 120 valid participants are planned to be recruited. Participants will be randomly assigned by computer, with 40 participants in each group. The three groups are expected to show no significant baseline differences in mental health, anxiety, affect, or heart rate, thereby ensuring comparability.

4. Research Design

This study adopts a randomized controlled trial (RCT) design and will strictly follow academic research and ethical standards.

4.1 Group Design

- Experimental group: AI large language model-assisted film music therapy group (n = 40).
- Standard group: standard music therapy group (n = 40).
- Control group: music-only relaxation group (n = 40).

4.2 Blinding Design

Participants will be blinded to the group allocation, the method used to generate the verbal guidance, and the study hypotheses in order to reduce experimental bias.

4.3 Assessment Design

The same psychological scales and physiological indicators will be used before and after the intervention to ensure that the data are objective and reproducible.

5. Research Intervention Procedure

5.1 Participant Recruitment and Informed Consent

University students who meet the inclusion criteria will be recruited. The researchers will explain the study purpose, study procedures, potential risks, data use, and the right to withdraw from the study. After confirming that participants fully understand the study, written informed consent will be obtained.

5.2 40-Minute Time-Constrained Cognitive Task

Before the formal intervention, all participants will complete a 40-minute time-constrained cognitive task to establish a relatively consistent pre-intervention psychological load and to induce a certain degree of mental fatigue, attentional resource depletion, and emotional stress, thereby providing a basis for observing the effects of the subsequent music intervention.

5.3 Pre-Intervention Assessment

Immediately after the cognitive task, a pre-intervention assessment will be conducted. The main measures will include mental health status, state anxiety, negative affect, and heart rate as psychological and physiological baseline indicators before the intervention.

5.4 30-Minute Group-Specific Intervention

After the pre-intervention assessment, participants will receive the corresponding 30-minute intervention according to random group allocation. The three groups will respectively receive: music combined with LLM-generated culturally adapted verbal suggestions; music combined with standardized verbal suggestions; or music-only intervention. Intervention duration, playback equipment, volume, and environmental conditions will be standardized across groups.

5.5 Post-Intervention Assessment

Immediately after the intervention, a post-intervention assessment will be conducted using the same mental health, anxiety, negative affect, and heart rate indicators as in the pre-intervention assessment. Immediate intervention effects will be evaluated by comparing pre-post changes within groups and differences among groups.

6. Assessment Indicators and Instruments

6.1 Core Indicators (Mental Health-Related)

- State-Trait Anxiety Inventory (STAI): used to assess state anxiety and trait anxiety. The scale was developed by Spielberger and colleagues, and previous research has confirmed its applicability among Chinese university students (Spielberger et al., 1983; Han et al., 2020).
- Positive and Negative Affect Schedule (PANAS): used to assess positive and negative affective states and to capture short-term emotional changes (Watson & Clark, 1994).

6.2 Physiological Indicator

- Heart rate: measured non-invasively using smartphone photoplethysmography to reflect physiological changes associated with psychological stress.

6.3 Manipulation Check

- Cultural resonance scale: used to verify the cultural adaptability of the AI-generated verbal guidance.

7. Data Collection and Analysis

7.1 Data Collection

Data collection in this study will include psychometric data and physiological indicators. All participants will complete the pre-intervention assessment after the 40-minute time-constrained cognitive task. The main measures will include mental health status, state anxiety, positive and negative affect, and heart rate. Participants will then receive a 30-minute music intervention according to random group allocation, including music combined with LLM-generated culturally adapted verbal suggestions, music combined with standardized verbal suggestions, or music-only intervention. Immediately after the intervention, the post-intervention assessment will be administered using the same psychological and physiological indicators as the pre-intervention assessment in order to record immediate pre-post changes.

7.2 Data Analysis

A two-way repeated measures analysis of variance (ANOVA) will be used to examine the effects of different intervention conditions and measurement time on the study outcomes. Group will be treated as the between-subjects factor, while time (pre-intervention vs. post-intervention) will be treated as the within-subjects factor. The analysis will focus on the main effect of group, the main effect of time, and the group-by-time interaction. Independent-samples t-tests will be used to compare baseline differences among groups before the intervention in order to assess baseline comparability (Ross & Willson, 2017).

In addition, to further analyze the relationships among changes in the study variables, a Linear Mixed Model (LMM) will be applied. Changes in relevant psychological indicators will be used as predictor variables to test whether they can predict changes in intervention outcome variables (Faul et al., 2009).

Before formal statistical analysis, tests of normality and homogeneity of variance will be conducted to determine whether the assumptions of repeated measures ANOVA are met. A significance level of $p < .05$ will be adopted. Partial eta-squared (ηp^2) will also be reported as an effect size measure to indicate the magnitude of effects associated with intervention condition and time. In addition to statistical significance, effect sizes and the practical significance of the findings will be considered to improve the completeness of interpretation (Cohen, 2013).

8. Researcher Qualifications

The applicant holds an Advanced Music Healing Practitioner Certificate issued by the Chinese human resources authority and is qualified to conduct music healing interventions. All intervention procedures will be independently administered by the applicant to ensure that the intervention process is standardized and consistent.

9. Ethical Risks and Risk-Control Measures

5. Risk assessment: This study involves a non-invasive, low-risk psychological relaxation intervention, with no physical trauma, no medication use, and no expected medication-related side effects.

6. Informed consent protection: Participants will be fully informed of the study purpose, procedures, and their rights. Participation will be voluntary, and participants may withdraw unconditionally at any time without affecting their studies, awards, or other rights and interests.
7. Privacy and confidentiality: All data will be anonymized. Personal identifiers such as names and student numbers will be removed. Data will be used only for academic analysis in this study and will be kept strictly confidential without disclosure.
8. Stress management: If a participant experiences discomfort, the intervention may be stopped immediately, and the researcher will provide immediate relaxation guidance to protect the participant's psychological safety.

10. Data Management and Use

9. Data collection, entry, and analysis will be standardized throughout the study, and outlier handling will follow statistical conventions.
10. Research data will be stored in encrypted form, accessible only to the research team, and will not be used for commercial purposes.
11. Research papers and reports will not disclose personally identifiable information, thereby protecting participant privacy.
12. After completion of the study, data will be retained and archived in accordance with the university's research management regulations.

11. Expected Outcomes

13. To demonstrate that AI large language model-assisted film music therapy can effectively improve the mental health of university students and alleviate anxiety.
14. To develop an AI-enabled, culturally adapted, and scalable music therapy intervention approach for university student mental health.
15. To provide empirical evidence for the ethical and standardized application of artificial intelligence in the field of mental health.

12. References

Amir, D. (1999). Musical and verbal interventions in music therapy: A qualitative study. Journal of Music Therapy, 36(2), 144-175. https://doi.org/10.1093/jmt/36.2.144

Cohen, J. (2013). Statistical power analysis for the behavioral sciences. New York: Academic Press.

Córdova Olivera, P., Gasser Gordillo, P., Naranjo Mejía, H., La Fuente Taborga, I., Grajeda Chacón, A., & Sanjinés Unzueta, A. (2023). Academic stress as a predictor of mental health in university students. Cogent Education, 10(2), 2232686. https://doi.org/10.1080/2331186X.2023.2232686

Dawson, A. F., Brown, W. W., Anderson, J., Datta, B., Donald, J. N., Hong, K., et al. (2020). Mindfulness-based interventions for university students: A systematic review and meta-analysis of randomised controlled trials. Applied Psychology: Health and Well-Being, 12(2), 384-410. https://doi.org/10.1111/aphw.12188

de Witte, M., da Silva Pinho, A., Stams, G.-J., Moonen, X., Bos, A. E. R., & van Hooren, S. (2022). Music therapy for stress reduction: A systematic review and meta-analysis. Health Psychology Review, 16(1), 134-159. https://doi.org/10.1080/17437199.2020.1846580

Demir, S., & Ercan, F. (2022). The effectiveness of cognitive behavioral therapy-based group counseling on depressive symptomatology, anxiety levels, automatic thoughts, and coping ways Turkish nursing students: A randomized controlled trial. Perspectives in Psychiatric Care, 58(4), 2394-2406. https://doi.org/10.1111/ppc.13073

Faul, F., Erdfelder, E., Buchner, A., & Lang, A.-G. (2009). Statistical power analyses using G*Power 3.1: Tests for correlation and regression analyses. Behavior Research Methods, 41(4), 1149-1160. https://doi.org/10.3758/BRM.41.4.1149

Guo, M., Jia, X., & Wang, W. (2024). How would you describe a mentally healthy college student based on Chinese culture? A qualitative research from the perspective of college students. BMC Psychology, 12(1), 207. https://doi.org/10.1186/s40359-024-01689-7

Han, Y., Fan, J., Wang, X., Xia, J., Liu, X., Zhou, H., et al. (2020). Factor structure and gender invariance of Chinese Version State-Trait Anxiety Inventory (Form Y) in university students. Frontiers in Psychology, 11, 2228. https://doi.org/10.3389/fpsyg.2020.02228

Harney, C., Johnson, J., Bailes, F., & Havelka, J. (2022). Is music listening an effective intervention for reducing anxiety? A systematic review and meta-analysis of controlled studies. Musicae Scientiae. https://doi.org/10.1177/10298649211046979

Liu, C., Tu, Y.-H., Lin, L.-J., Chen, H., Liu, T.-H., Lin, H.-L., et al. (2025). Doctor-patient communication models, patient decision-making participation, and patient emotional expression: A cross-cultural comparison of samples from the UK and China. Patient Preference and Adherence, 19, 2505-2524. https://doi.org/10.2147/PPA.S537829

Liu, M., Zhang, L. J., & Biebricher, C. (2024). Investigating students’ cognitive processes in generative AI-assisted digital multimodal composing and traditional writing. Computers & Education, 211, 104977. https://doi.org/10.1016/j.compedu.2023.104977

Nolan, P. (2005). Verbal processing within the music therapy relationship. Music Therapy Perspectives, 23(1), 18-28. https://doi.org/10.1093/mtp/23.1.18

Ross, A., & Willson, V. L. (2017). Basic and advanced statistical tests: Writing results sections and creating tables and figures. Rotterdam: SensePublishers. https://doi.org/10.1007/978-94-6351-086-8

Sheldon, E., Simmonds-Buckley, M., Bone, C., Mascarenhas, T., Chan, N., Wincott, M., et al. (2021). Prevalence and risk factors for mental health problems in university undergraduate students: A systematic review with meta-analysis. Journal of Affective Disorders, 287, 282-292. https://doi.org/10.1016/j.jad.2021.03.054

Spielberger, C. D., Gorsuch, R. L., Lushene, R., Vagg, P. R., & Jacobs, G. A. (1983). Manual for the State-Trait Anxiety Inventory. Palo Alto, CA: Consulting Psychologists Press.

Strawn, J. R., Geracioti, L., Rajdev, N., Clemenza, K., & Levine, A. (2018). Pharmacotherapy for generalized anxiety disorder in adult and pediatric patients: An evidence-based treatment review. Expert Opinion on Pharmacotherapy, 19(10), 1057-1070. https://doi.org/10.1080/14656566.2018.1491966

Watson, D., & Clark, L. A. (1994). The PANAS-X: Manual for the Positive and Negative Affect Schedule-Expanded Form. Ames: University of Iowa.

Wu, J., Zhang, L., Zhu, X., & Jiang, G. (2021). Mental health literacy from the perspective of multi-field experts in the context of Chinese culture. International Journal of Environmental Research and Public Health, 18(4), 1387. https://doi.org/10.3390/ijerph18041387

Yang, M., Li, X., Qin, X., Tian, X., Zhang, H., & Wen, H. (2025). The relationship between perceived academic stress and college students' employment anxiety: The mediating role of psychological resilience. Frontiers in Psychiatry, 16, 1602808. https://doi.org/10.3389/fpsyt.2025.1602808
