# Supplementary material for: Integrating large language model-generated culturally adapted verbal suggestions into music therapy for university students: A pilot randomized exploratory study
Source: PLoS One. 2026 Oct 9;21(10):e0360478. doi: 10.1371/journal.pone.0360478 (PMC13652252; doi:10.1371/journal.pone.0360478)
Supplement: Supplementary file 4 [file pone.0360478.s005.docx]

**Response to Reviewers**

**Manuscript Title:** *The Effectiveness of Music Therapy Incorporating Culturally Adapted Verbal Suggestions Generated by Large Language Models: A Randomized Controlled Trial*

Dear Editor and Reviewers,

Thank you for the opportunity to revise and resubmit our manuscript. We sincerely appreciate the time, effort, and constructive comments provided by the Editor and all five reviewers. Their insightful suggestions were highly valuable and helped improve the clarity, rigor, and overall quality of the manuscript.

We carefully considered every comment and revised the manuscript accordingly. Below, we provide a detailed point-by-point response to each reviewer’s comments. We hope that the revised manuscript meets the journal’s standards and we sincerely appreciate your further consideration.

Sincerely,

The authors

**Response to Reviewer #1**

We sincerely thank Reviewer #1 for the thoughtful and constructive comments. The suggestions were highly valuable and helped strengthen the manuscript.

**Comment 1**

Authors should clarify how the 30 minute soundtrack from Hero was segmented and whether the verbal suggestions were timed to align with specific musical passages.Please see and add [1] Generation of Voice Signal Tone Sandhi and Melody Based on Convolutional Neural Network.

**Response**

We have reviewed and cited the references [34] you recommended, and we sincerely appreciate your suggestions.

In the present study, the 30-minute soundtrack was segmented into seven relatively independent musical sections (each approximately 3–5 minutes), based on the original compositional structure of the film score. These sections are titled: Hero Overture, For the World, Warriors, Longing, Swift Sword, Yearning for the Peace, and Farewell Hero.

The verbal suggestions were not fully aligned with all musical sections; instead, they were selectively embedded within four sections. Specifically, these sections correspond to key emotional phases of the intervention, including emotional engagement (For the World), tension and release (Warriors and Swift Sword), and emotional integration and relaxation (Yearning for the Peace). In addition, these segments exhibit clear musical structures and smooth emotional transitions. Following multiple rounds of discussion between the researchers and certified music therapists, it was determined that embedding verbal suggestions within these sections would not disrupt the listener’s immersive experience.

**Revisions made in manuscript**

Page 5, Lines 99–106，Update the list of references at the same time.

**Comment 2**. How does the study control for the confounding variable of therapist presence, given that only the two verbal suggestion groups involved a live therapist reading scripts?

**Response**

Thank you for this important comment. We acknowledge that therapist presence may represent a potential confounding variable. To minimize this effect, several standardization procedures were implemented. First, the same therapist conducted all intervention sessions across all groups, thereby eliminating variability arising from individual differences between therapists. Second, procedural interactions (e.g., instructions, monitoring, and session management) were kept consistent across all experimental conditions to control for non-specific interpersonal effects.

**Revision in Manuscript**

Methods section	Procedure, Page 6, Lines 133–139

**Comment 3.** Please explain why heart rate data violated normality assumptions and whether non parametric tests were appropriately applied to change scores rather than raw values.

**Response**

Thank you for this important comment.

Regarding the violation of normality assumptions, heart rate data were assessed using the Shapiro–Wilk test prior to analysis and were found to deviate from a normal distribution. This pattern is consistent with established findings in psychophysiological research, which indicate that heart rate, as a physiological measure regulated by the autonomic nervous system, often exhibits non-linear dynamics and skewed distributions (Berntson et al., 1997).

Accordingly, group differences in heart rate changes were analyzed using the Kruskal–Wallis test based on change scores rather than raw heart rate values. Where applicable, post hoc pairwise comparisons were conducted using Dunn’s test with adjusted p-values. This approach ensured that the analyses specifically reflected intervention-related changes while minimizing potential bias from baseline differences.

These clarifications have been added to the revised Methods section to improve methodological rigor.

**Revision in Manuscript**

Data Analysis , Page 9, Lines 199–205

**Comment 4.** Authors should justify the use of a 40 minute examination task for stress induction without reporting manipulation checks or post induction baseline measures.

**Response**

Thank you for this important comment. Thank you for this valuable comment. A post-induction baseline assessment was conducted immediately after the 40-minute examination task and before the intervention. However, this procedure was not clearly described in the original manuscript. We have now clarified this point in the revised Methods section. The examination task was selected based on prior studies showing that cognitively demanding tasks under time pressure can effectively induce acute stress responses in student populations [37]. In addition, we acknowledge that the present study lacked formal manipulation checks for the stress induction procedure. We have now added a corresponding statement in the Limitations section of the manuscript to address this issue. We agree that this aspect can be further improved in future research.

**Revision in Manuscript**

Methods Page 7, Lines 141–147

Limitations Page 16, Lines 358–360

**Comment 5.** How does the study ensure that the "standard verbal suggestions" represent actual clinical practice rather than an artificially constructed comparator?

**Response**

Thank you for this insightful comment.

The “standard verbal suggestions” used in this study were not artificially constructed but were adapted from commonly used scripts in clinical music therapy and relaxation training, such as breathing guidance and imagery-based facilitation. Specifically, the content of the standard verbal suggestions was derived from Introduction to Music Therapy by Professor Tian Gao, a well-established music therapy scholar who has played a pioneering role in the development of music therapy education and clinical practice in China.

According to the reviewer’s suggestion, we have clarified this point in the revised Methods section to further improve the transparency of the study design.

**Revision in Manuscript**

Intervention Group 2: Standard music therapy group Page 6, Lines 116–121

**Comment 6.** Please provide inter rater reliability data for the therapist's delivery of verbal suggestions to ensure consistency across participants within groups.

**Response**

Thank you for this valuable comment.

In the present study, all intervention sessions were conducted by the same trained music therapist. Therefore, inter-rater reliability was not applicable, as there were no multiple raters or therapists involved in delivering the intervention. In addition, procedural interactions were structured and systematically implemented.

We have clarified this point in the revised Methods section (Procedure).

**Revision in Manuscript**

Methods section, Page 7, Lines 138–139

**Comment 7.** Authors should report a priori power analysis and explain whether the sample size of 113 participants was sufficient to detect expected effect sizes.

**Response**

Thank you for this important comment.

We acknowledge that a formal a priori power analysis was not conducted before data collection, which is a limitation of the present study. To address the reviewer’s concern, we conducted a sensitivity power analysis using G*Power for a one-way ANOVA with three groups, assuming an alpha level of 0.05 and a desired power of 0.80. The analysis indicated that a total sample size of 113 participants would be detect an effect size of approximately f = 0.30.

We have added this clarification to the revised manuscript and acknowledged the limitation related to statistical power in the Discussion section.

**Revision in Manuscript**

Data Analysis and Limitations

Page 10 , Lines 208–210 . Page 16 , Lines 353–356.

**Comment 8.** How does the study address potential demand characteristics, given that participants in the Eastern imagery group might guess the cultural tailoring hypothesis?

**Response**

Thank you for this insightful comment.

We acknowledge that demand characteristics may have influenced participants’ responses, particularly in the culturally tailored condition. To mitigate this risk, participants were not informed of the study hypotheses, all conditions were presented as equally valid interventions, and procedures were standardized across groups.

Thank you for your comment. We have added a detailed description of this point in the Procedure section of the revised manuscript.

**Revision in Manuscript**

Procedure, Page 7, Lines 133–135.

**Comment 9.** Please clarify why the PANAS positive affect showed no changes whatsoever, and whether ceiling or floor effects might explain this null finding.

**Response**

Thank you for this important comment.

The absence of significant changes in positive affect (PA) was examined in relation to potential ceiling or floor effects. Baseline PA scores across the three groups were moderate (ranging from 2.51 to 2.89 on a 5-point scale), indicating that neither ceiling nor floor effects were present. Therefore, the null findings are unlikely to be explained by restricted variance at baseline.

Instead, this pattern is consistent with previous research. Prior studies have shown that music-based interventions are generally more effective in reducing negative affect than in enhancing positive affect (Blasco-Magraner et al., 2023). In addition, music listening has been demonstrated to effectively regulate negative emotional states following stress induction (Groarke and Hogan, 2019).

We have added further explanation and discussion of this issue in the revised Discussion section.

**Revision in Manuscript**

Discussion, Page 15, Lines 321–325.

**Comment 10.** Authors should explain how the eight musical pieces from Kikujiro were selected and whether their joyful character might confound comparisons with the other groups.

**Response**

We sincerely thank the reviewer for this thoughtful and important comment. We have revised the manuscript to clarify the rationale for selecting the eight tracks from Kikujiro.

The music was selected based on several criteria: (1) instrumental compositions without lyrics, to avoid verbal interference; (2) moderate tempo and emotionally accessible melodic structure; (3) a progressive emotional flow from mild activation to relaxation. The selected tracks were arranged to create a coherent transition from light positive arousal toward calmness and emotional stabilization.

Regarding the cheerful character of some tracks, we agree that musical valence may influence participants’ responses. However, this condition was intentionally designed as a passive music-listening relaxation control that reflected a positive but non-directive emotional environment, without verbal guidance or therapist-led intervention.

We have also acknowledged in the revised Discussion

**Revision in Manuscript**

Methods	Page 6, Lines 122–127.

Limitations Page 16, Lines 356–358.

**Comment 11.** How does the study account for participants' prior familiarity with or emotional associations to the film soundtracks used as stimuli?

**Response**

Thank you for this insightful comment.

We acknowledge that participants’ prior familiarity with the musical materials may have influenced their emotional responses to some extent. As the primary focus of this study was on the role of verbal guidance in music-based interventions. Random assignment and standardized intervention procedures were implemented to help control for this potential confounding factor. We have also acknowledged in the revised Limitations that familiarity across conditions may have introduced potential confounding effects.

**Revision in Manuscript**

Methods Procedure Page 7, Lines 133–139. Limitations Page 16, Lines 356–358.

**Comment 12.** Please provide the full ChatGPT prompt in the methods section, including any temperature settings or other parameters that might affect output reproducibility.

**Response**

Thank you for this important comment.

To enhance transparency and reproducibility, we have now provided the full prompt structure used for generating the verbal suggestions in the Methods section. The prompts were originally written in Chinese, as the study was conducted in a Chinese-language context. For clarity and accessibility, both the original Chinese prompts and their English translations have been included in the revised manuscript.

**Revision in Manuscript**

Methods, Materials

Page 5, Lines 92–98.

**Comment 13.** Authors should justify the exclusion of outliers from STAI and PANAS data while retaining all heart rate data, and report sensitivity analyses with outliers included.

**Response**

Thank you for this important suggestion.

In the original manuscript, outliers were identified and excluded using the standard ±1.5 × interquartile range (IQR) criterion to enhance the robustness of the data. This approach was applied to the self-report measures (STAI and PANAS), as such measures are more susceptible to response biases, particularly when completed within a short time frame.

In contrast, no extreme outliers exceeding the IQR thresholds were detected in the heart rate (HR) data. Moreover, HR values in young adults typically fall within a relatively stable physiological range (approximately 40–120 bpm). Therefore, all HR data were retained for analysis.

Following the reviewer’s recommendation, we conducted additional sensitivity analyses by re-running the primary statistical tests with all previously excluded outliers included. The results indicated that the main findings remained consistent. Specifically, the within-group reduction in state anxiety in the Eastern imagery group remained significant (original p = .030; sensitivity analysis p = .046). The significant reductions in negative affect in both the Eastern imagery and standard groups were also preserved. Furthermore, all between-group comparisons using ANOVA and Kruskal–Wallis tests remained non-significant.

The results of these sensitivity analyses have been added to the revised manuscript in the Results section (under a new subsection, “Sensitivity Analyses”), and the corresponding procedures have been clarified in the Data Analysis section of the Methods. These revisions improve the transparency and robustness of the study.

**Revision in Manuscript**

Results and Data Analysis	Page 13, Lines 283–289. Page 10, Lines 214–219.

**Comment 14.** How does the study ensure that the Eastern imagery verbal suggestions actually reflect culturally adapted content rather than generic poetic language?

**Response**

Thank you for this insightful comment, which highlights a critical aspect of cultural adaptation in the present study.

To ensure that the verbal suggestions in the Eastern imagery group genuinely reflected culturally tailored content rather than generic poetic language, several steps were implemented. First, all prompts input into ChatGPT 5.2 were written in Chinese and explicitly instructed the model to incorporate “Eastern imagery and culturally symbolic elements.” The second prompt further required the model to generate suggestive language “based on the characteristics of the Chinese film Hero.”

In addition, Table 1 has been added to the revised Methods section to provide a comparative illustration between generic relaxation scripts and the culturally adapted versions used in this study.

The generated scripts were independently reviewed and iteratively refined by the research team. The team included one author with expertise in Chinese traditional literature and a certified music therapist. Through multiple rounds of revision, culturally specific imagery (e.g., qin music, eastern wind, mountains and rivers, breath) was strengthened, while generalized relaxation expressions were minimized.

**Revision in Manuscript**

We have expanded the description of this procedure in the Methods section (Intervention Group 1) to improve the transparency of the study. Page 5, Lines 107–114.

**Comment 15.** Authors should discuss why the future scope should contain explanability and newer transformer models, please see and add [2] Tri-STANet: An advanced framework integrating dual axis transformer and morphological encoding for enhanced AS detection; [3] XAI-RACapsNet: Relevance aware capsule network-based breast cancer detection using mammography images via explainability O-net ROI segmentation

**Response**

Thank you for this valuable suggestion. We agree that both explainability and the use of newer transformer-based models represent important directions for future research.

We have reviewed and cited the references you recommended [55,56], and we sincerely appreciate your suggestions, which have greatly enriched our understanding of this topic. Specifically, the primary aim of the present study was to provide an initial empirical test of the feasibility of using large language models to generate culturally adapted verbal suggestions in music-based interventions. Therefore, future research should incorporate explainability analyses to clarify which linguistic features, cultural symbols, and modes of expression contribute most to therapeutic effectiveness and cultural resonance.

**Revision in Manuscript**

Discussion, future research Page 16, Lines 362–363.

**Comment 16.** Please explain why trait anxiety moderation analyses were underpowered, with only seven participants in the low trait anxiety subgroup of the Eastern imagery condition.

**Response**

Thank you for the reviewer’s careful evaluation. We agree that the analysis of the moderating effect of trait anxiety within the Eastern imagery group may have been underpowered due to sample imbalance, which in turn limits the statistical power of subgroup analyses.

In the revised manuscript, we have explicitly acknowledged this limitation and emphasized that the non-significant findings should be interpreted with caution. Furthermore, we suggest that future research should increase the sample size and strive to balance the proportion of participants with high and low trait anxiety in order to ensure sufficient statistical power.

**Revision in Manuscript**

Results Page 14, Lines 297–300.

Limitations Page 16, Lines 353–361.

**Comment 17.** Authors should clarify whether heart rate measurements were taken immediately post intervention or after a delay, and whether room conditions were standardized.

**Response**

Thank you for this important comment.

Heart rate measurements were obtained immediately after the completion of the intervention, without delay, in order to capture the immediate physiological response to the music-based intervention. In addition, all heart rate measurements were conducted under standardized environmental conditions, including controlled room temperature, lighting, and noise levels, to minimize potential external influences on physiological responses.

**Revision in Manuscript**

These details have now been clarified in the revised manuscript (Methods section).

Page 8, Lines 181–184.

**Comment 18**. How does the study address the multiple comparison problem given the numerous t tests and ANOVAs performed without correction for family wise error rate?

**Response**

Thank you for raising the issue of multiple comparisons. In response, we have conducted post-hoc LSD tests on the significant ANOVA results. The key findings remain as follows:

The Eastern imagery group showed significantly lower Negative Affect scores post-intervention compared to both the standard and music-only groups. The standard music therapy group exhibited a significantly greater reduction in heart rate compared to the relaxation music group (*p* = .006). However, the difference between the standard and Eastern imagery groups was not significant (*p* = .09), nor was the difference between the Eastern imagery and relaxation music groups (*p* = .48)

**Revision in Manuscript**

These post-hoc results have been added to the revised Results section Page 13, Lines 275–279.

**Comment 19**. Please provide the exact duration of each intervention and confirm that exposure time was precisely equivalent across all three experimental conditions.

**Response**

Thank you for this important question regarding intervention duration.

Upon re-checking the actual audio files used in the study, we confirm the precise durations as follows:

- Eastern imagery group (Group 1): 26 minutes 19 seconds
- Standard verbal suggestion group (Group 2): 29 minutes 56 seconds
- Music-only group (Group 3): 25 minutes 35 seconds

We acknowledge that the intervention lengths were not perfectly equivalent across the three conditions This variation occurred because we used the original commercial recordings without artificial editing to preserve the ecological validity and natural flow of the music. In the revised manuscript, we have now clearly reported the exact durations for each condition and discussed this as a minor limitation. Although the durations are not identical, all interventions fell within a narrow 25–30minute range, which is typical in music therapy research.

**Revision in Manuscript**

Methods

Page 5, Lines 89. Page 6, Lines 118. Page 6, Lines 128.

**Response to Reviewer #2**

Thank you very much for your insightful and constructive comments. These suggestions have significantly improved the rigor, clarity, and methodological transparency of the manuscript. We have carefully revised the manuscript accordingly.

**Comment 1.**Introduction should be shorted to maximun 1 page and a half

**Response**

Thank you for this valuable suggestion. We have substantially revised and condensed the Introduction section to improve clarity and conciseness. Specifically:

- Redundant descriptions of general psychological interventions (e.g., CBT, pharmacological treatments) have been reduced.
- Overlapping literature statements have been merged.
- Greater focus has been placed on the core themes of this study, including music therapy mechanisms, cultural adaptation of verbal suggestions, and the role of large language models (LLMs).

As a result, the Introduction has been reduced to approximately 1.5 pages.

**Revision in manuscript:**

The Introduction section (Pages 2 Lines 24–69.) has been shortened and streamlined accordingly.

**Comment 2** methods: it should be added who adjudicated the quality of life

**Response**

Thank you for this valuable comment. Quality of life was not included in the present study. We acknowledge its importance in comprehensively evaluating intervention outcomes and will consider incorporating it in future research.

**Comment 3** methods: it is not clear if authors used block sizes for the rct

**Response**

Thank you for this important comment. We acknowledge that block randomization may improve group balance, particularly in smaller samples. In the present study, participants were assigned using a computer-generated simple randomization procedure, and no block randomization or fixed block sizes were applied. We will consider adopting this approach in future research. Thank you again for this valuable suggestion.

**Revision in Manuscript**

The Procedure section (Page 7 Lines 133–139.) has been revised.

**Comment 4** methods: sample size calculation should be improved

**Response**

We appreciate this important suggestion. We acknowledge that a formal a priori sample size calculation was not conducted prior to recruitment. Instead, we performed a sensitivity power analysis using G*Power based on the final sample size. The analysis indicated that: With N = 113, α = 0.05, and power = 0.80. The study was adequately powered to detect a medium effect size (f = 0.30).

We have clarified this in the Methods section and acknowledged it as a limitation in the Discussion section.

**Revision in Manuscript**

Methods (Page 9 Lines 208–210.)

Discussion (Page 16 Lines 354–356.)

**Comment 5** methods/results: do authors think that embedding multivariate analysis may increase the power of the analysis?

**Response**

Thank you for this insightful and constructive suggestion. We agree that multivariate analytical approaches offer several advantages, including the ability to simultaneously analyze multiple dependent variables, account for correlations among variables, and improve statistical power. However, in the present study, some variables (i.e., anxiety and PANA) met the assumption of normality, whereas heart rate data did not. Therefore, a multivariate model may not applied. Thank you again for this valuable suggestion.

**Response to Reviewer #3**

Thank you very much for your insightful and constructive comments. These suggestions have significantly improved the rigor, clarity, and methodological transparency of the manuscript. We have carefully revised the manuscript accordingly. Below, we provide a point-by-point response.

**Comment 1**

The sample size appears underpowered to detect between-group differences, especially given the small effect sizes reported and the use of three groups without a clear a priori power calculation.

**Response**

Thank you for this important comment. We acknowledge that an a priori power analysis was not conducted prior to the study, which may have limited the statistical power to detect between-group differences. To address this limitation, a sensitivity analysis has been added to the Methods section, indicating that the current sample size (N = 113) is sufficient to detect a medium effect size (f = 0.30).

In addition, the overall effect sizes reported in the study were relatively small, suggesting that between-group differences may be limited. This limitation has been further emphasized in the Discussion section, and future research is recommended to employ larger sample sizes to improve statistical power.

**Revision in Manuscript**

Power analysis has been add in the Methods section Page 10 Lines 208–210

A statement regarding sample size and effect size limitations has been added to the Discussion section Page 16 Lines 354–356

**Comment (2)**

It’s unclear why different film soundtracks were used across the three conditions.

**Response**

Thank you for this important question. We apologize for not clearly explaining the rationale for using different film soundtracks across the three conditions in the original manuscript.

The selection of different music was intentional and directly aligned with the theoretical purpose of each experimental arm:

The Eastern imagery group (Group 1) used the soundtrack from the Chinese film *Hero* (composed by Tan Dun) because its strong Eastern aesthetic elements (e.g., guqin, traditional orchestration, and philosophical themes) complemented the LLM-generated culturally adapted verbal suggestions.

The standard verbal suggestion group (Group 2) used therapeutic relaxation music recommended in the textbook *Introduction to Music Therapy* by Tian Gao. This represents the conventional, culturally neutral approach commonly used in clinical receptive music therapy, serving as an active control for general verbal guidance.

The music-only group (Group 3) used the soundtrack from the Japanese film *Kikujiro* (composed by Joe Hisaishi), chosen for its gentle, joyful, and childlike qualities that facilitate passive emotional regulation without any verbal suggestions. This group served as a passive control.

We acknowledge that using different musical materials may introduce some confounding (e.g., differences in emotional tone or familiarity), and we have added this as a limitation in the Discussion section.

**Revision in Manuscript**

Methods section Page 4 Lines 85–131

Discussion section Page 16 Lines 356–359

**Comment (3)** The stress induction task lasted 40 minutes but is described only vaguely; without details on cognitive load or validation of its effectiveness, its role as a standardized stressor remains questionable.

**Response**

Thank you for this valuable comment. We have clarified and refined the description of the stress induction task in the revised manuscript.

Specifically, after obtaining written informed consent, participants completed a **40-minute time-constrained cognitive task**, in which they were required to organize course-related content and construct a structured mind map. This task was designed to simulate a cognitively demanding academic activity. Prior research has demonstrated that **cognitive load, defined as the mental effort involved in processing and organizing information, when combined with time constraints, can effectively induce stress responses in experimental settings** [37].

The manuscript has been revised to provide a clearer description of the task, thank you.

**Revision in Manuscript**

Methods, Procedure Page 7 Lines 133–152

**Comment (4)** Heart rate was measured using a smartphone app, yet no information is provided about calibration, participant positioning, or consistency of measurement technique across individuals.

**Response**

Thank you for this important comment. We have clarified the heart rate measurement procedure in the revised manuscript.

In the present study, heart rate was measured using a smartphone application based on **photoplethysmography (PPG).** Previous studies have demonstrated that smartphone-based PPG measurements show **good agreement with validated methods such as electrocardiography (ECG)** under resting conditions.

To enhance measurement consistency, participants were instructed to remain seated in a relaxed position, minimize movement, and maintain consistent finger placement during measurement. Measurements were conducted under controlled environmental conditions (e.g., stable lighting and minimal noise) immediately after the intervention.

**Revision in Manuscript**

Heart Rate, Page 8 Lines 177–188

**Comment (5)** The Eastern imagery script uses poetic metaphors, but there’s no evidence that participants actually perceived these as culturally resonant or more acceptable than standard suggestions.

**Response**

Thank you for this insightful comment. We agree that it is important to provide evidence that participants actually perceived the Eastern imagery verbal suggestions as culturally resonant.

To address this concern, we have added a post-hoc manipulation check in the revised manuscript. Specifically, after the intervention, participants in the Eastern imagery group rated the verbal suggestions on two 7-point Likert scales: (1) the extent to which the suggestions felt culturally relevant to Chinese culture, and (2) the extent to which the suggestions felt personally resonant and meaningful to them (1 = not at all, 7 = extremely).

The results showed that participants perceived the Eastern imagery verbal suggestions as highly culturally relevant (M = 5.03, SD = 1.18) and personally resonant (M = 5.23, SD = 1.03). Both ratings were significantly higher than the scale midpoint of 4.

**Revision in Manuscript**

We have incorporated these manipulation check results into the Methods and Results sections. Page 9 Lines 189–193, Page 12 Lines 262–1266

**Comment (6)** Trait anxiety was used to stratify a subgroup analysis, but the low-anxiety group had only seven participants, making any comparison statistically unreliable and possibly misleading.

**Response**

Thank you for this insightful comment. The authors agree that the small sample size of the low trait anxiety group (n = 7) may limit the stability of the statistical analysis.

In addition, we would like to clarify a potential procedural factor that may have contributed to this issue. In the present study, participants completed a **40-minute cognitively demanding task prior to the administration of the STAI-T**, which may have influenced the assessment of trait anxiety.

This issue has now been acknowledged as a limitation in the revised manuscript. Future research should administer trait anxiety measures prior to experimental tasks to ensure more accurate assessment.

**Revision in Manuscript**

To minimize the risk of overinterpretation, the results have been **reported with caution**, Page 14 Lines 297–300. This limitation has been explicitly acknowledged in the revised Discussion section. Page 16 Lines 360–362

**Comment (7)** Although ChatGPT 5.2 is named, there’s no description of prompt engineering, temperature settings, or how output consistency was ensured across potential multiple generations.

**Response**

Thank you for this important comment.

To enhance transparency and reproducibility, we have now provided the full prompt structure used for generating the verbal suggestions in the Methods section. The prompts were originally written in Chinese, as the study was conducted in a Chinese-language context. For clarity and accessibility, both the original Chinese prompts and their English translations have been included in the revised manuscript.

**Revision in Manuscript**

Methods Page 5 Lines 92–98

**Comment (8)** The claim that LLM-generated content is “culturally adaptive” rests solely on researcher interpretation rather than empirical validation from cultural experts or target-user feedback.

**Response**

Thank you for your valuable suggestion. We fully agree that cultural adaptation is the core mechanism underlying the effects of this study.

In the revised manuscript, we have added a post-hoc manipulation check. Specifically, after the intervention, participants in the Eastern imagery group rated the cultural relevance and acceptability of the verbal suggestions using a 7-point Likert scale (1 = not at all culturally relevant/acceptable, 7 = highly culturally relevant/acceptable).

The results showed that participants perceived the Eastern imagery verbal suggestions as highly culturally relevant (M = 5.03, SD = 1.18) and acceptable (M = 5.23, SD = 1.03), with both ratings being significantly higher than the midpoint score of 4. We have added the results of this manipulation check to the Methods and Results sections of the revised manuscript.

We believe that these additions strengthen the evidence for cultural adaptation as an important mechanism and enhance the mechanistic interpretation of this study.

**Revision in Manuscript**

Methods Page 9 Lines 189–193; Results Page 12 Lines 262–266

**Comment (9)** Passive music listening used Joe Hisaishi’s compositions, which are emotionally evocative—this may not serve as an inert control, potentially diluting observed differences between active and passive conditions.

**Response**

Thank you for this important comment. The authors acknowledge that the music of Joe Hisaishi is emotionally evocative, which may have influenced participants’ affective responses. This potential effect has now been addressed in the Discussion section of the revised manuscript. Future studies are recommended to use more neutral stimuli as control conditions to minimize such confounding influences.

**Revision in Manuscript**

Methods Page 6 Lines 122–131; Discussion Page 16 Lines 356–358

**Comment (10)** Outlier removal followed IQR rules, but with small group sizes (e.g., n=36), excluding even two outliers could disproportionately influence mean estimates and p-values.

**Response**

Thank you for this important suggestion.

In the original manuscript, outliers were identified and excluded using the standard ±1.5 × interquartile range (IQR) criterion to enhance the robustness of the data. This approach was applied to the self-report measures (STAI and PANAS), as such measures are more susceptible to response biases, particularly when completed within a short time frame.

We conducted additional sensitivity analyses by re-running the primary statistical tests with all previously excluded outliers included. The results indicated that the main findings remained consistent. Specifically, the within-group reduction in state anxiety in the Eastern imagery group remained significant (original p = .030; sensitivity analysis p = .046). The significant reductions in negative affect in both the Eastern imagery and standard groups were also preserved. Furthermore, all between-group comparisons using ANOVA and Kruskal–Wallis tests remained non-significant.

The results of these sensitivity analyses have been added to the revised manuscript in the Results section (under a new subsection, “Sensitivity Analyses”), and the corresponding procedures have been clarified in the Data Analysis section of the Methods. These revisions improve the transparency and robustness of the study.

**Revision in Manuscript**

Methods and the Data Analysis Page 10 Lines 214–219; Page 13 Lines 283–289

**Comment (11)** The correlation between heart rate and state anxiety change is modest yet it’s presented as meaningful support for physiological–subjective alignment without considering alternative explanations like regression to the mean.

**Response**

Thank you for this insightful comment. The authors agree that the observed correlation between changes in heart rate and state anxiety was modest and should be interpreted with caution.

In response, we have revised the manuscript to **temper the interpretation of this finding** and avoid overstating the alignment between physiological and subjective measures. This limitation has also been explicitly acknowledged in the Discussion section.

**Revision in Manuscript**

Results Page 13 Lines 268–271; Discussion Page 9 Lines 338–342

**Response to Reviewer #4**

Thank you for the reviewer’s positive and constructive evaluation of our work. We appreciate the recognition of the study’s innovation and methodological transparency. We have carefully revised the manuscript to address all comments and suggestions in detail.

**Comment 1‎** Abstract: The section needs to be re-drafted to be self-contained means it has to clearly ‎show the hypothesis, methodology, techniques, and tools used, and the results obtained.‎

**Response：**

Thank you for this valuable suggestion. The abstract has been fully revised to ensure it is self-contained. It now clearly presents the research hypothesis, study design (randomized controlled trial), techniques employed (LLM-generated verbal suggestions), key measurement instruments, and the main results. These revisions improve both the completeness and readability of the abstract.

**Revisions made in manuscript**

Abstract Page 2, Lines 10–21，

‎**Comment 2** Keywords: The Authors suggested updating the keywords by selecting more relevant terms. ‎Keywords play an important role in the appearance of the manuscript in scholars' searches, ‎which will give it more hits and more citations.‎

**Response：**Thank you for this suggestion. The keywords have been revised to include more specific and relevant terms (e.g., “large language model,” “music therapy,” “cultural adaptation,” “anxiety,” and “emotion regulation”) to enhance the manuscript’s visibility and indexing in academic databases.

**Comment 3**

The manuscript would benefit from more detailed reporting of how the AI-generated scripts were produced and standardized. What specific prompts and parameters were used, and how was consistency ensured across participants?
**Response：**

Thank you for this important comment.

To enhance transparency and reproducibility, we have now provided the full prompt structure used for generating the verbal suggestions in the Methods section. The prompts were originally written in Chinese, as the study was conducted in a Chinese-language context. For clarity and accessibility, both the original Chinese prompts and their English translations have been included in the revised manuscript.

**Revisions made in manuscript**

Page 5, Lines 92–98，

**Comment 4**

Could the authors clarify the procedures for randomization, allocation, and blinding? Additionally, were participants aware that some scripts were AI-generated, and if so, might this have influenced outcomes?
‎ **Response：** Thank you for this important comment. We have revised the Methods section to clarify the procedures for randomization, allocation, and blinding.

Participants were assigned to the three conditions using a computer-generated simple randomization procedure. Allocation was conducted prior to the intervention, and group assignments were implemented by the researcher following the randomization sequence. Regarding blinding, participants were not informed of the specific study hypotheses or the origin of the verbal suggestions. In particular, they were not told whether the scripts were generated by AI or developed using conventional methods. All intervention conditions were presented as equally valid forms of music-based relaxation. This approach was adopted to minimize expectancy effects and demand characteristics.

**Revisions made in manuscript**

Procedure Page 7, Lines 132–1139，

**Comment 5**

Introduction: The authors advised adding one more paragraph at the end of the section to ‎show the organization of the rest of the paper.‎
‎ **Response：**Thank you for this helpful suggestion. We have added a paragraph at the end of the Introduction to clearly outline the structure of the manuscript. This addition improves the organization and readability of the paper.

**Revisions made in manuscript**

Introduction	Page 4, Lines 66–69，

**Comment 6**

The study suggests that culturally tailored, AI-generated imagery may help reduce state anxiety. However, is the observed improvement truly due to cultural resonance, or could it reflect expectancy effects, novelty of the AI component, or therapist influence during delivery?
**Response：**

Thank you for this insightful and important comment. We agree that the observed effects may not be solely attributable to cultural resonance and could be influenced by alternative factors such as expectancy effects, or therapist-related factors.

In this study, several steps were taken to reduce potential bias. Participants were not informed of the specific research hypotheses. They were also not told whether the intervention content was generated by artificial intelligence. In addition, all interventions were delivered by the same therapist using a standardized procedure.

In addition, to further clarify the influence of cultural resonance on our study outcomes, we supplemented manipulation checks to verify the cultural compatibility and perceived credibility of the Eastern imagery verbal suggestions. Relevant details can be found in our response to Comment 9.

The corresponding revisions have been incorporated into the main text: Methods, Page 9, Lines 189–193, Results, Page 12, Lines 262–266

**Comment 7**

Should AI-generated verbal suggestions be viewed as an active therapeutic mechanism in their own right, or are they simply tools that depend heavily on how clinicians implement and contextualize them?
‎ **Response：**

Thank you for raising this insightful and important question. It touches on the fundamental role that AI-generated verbal suggestions may play within therapeutic contexts.

In this study, we tend to conceptualize AI-generated verbal suggestions as a potential active therapeutic mechanism in their own right. In fact, emerging digital mental health applications provide support for this perspective. For example, AI-based systems such as *Therabot* have demonstrated the feasibility of delivering therapeutic interventions through dialogue.

At the same time, we acknowledge that, in the present study, the effectiveness of AI-generated verbal suggestions still depends to some extent on clinician guidance and contextualization. It is also worth noting that this line of research is being further extended in our ongoing work. Our research team has recently secured external funding and is currently collaborating with AI specialists to develop an intervention application that integrates AI-driven dialogue, verbal suggestion generation, and personalized music. Thank you very much.

**Comment 8**

Conclusion: The conclusion should be abstracted, so the authors should consider re-‎drafting it.‎

**Response：**

Thank you for this important comment. The conclusion section has been **revised and rewritten** to be more **generalized** and to avoid **overgeneralization / excessive inference.**

**Revisions made in manuscript**

Conclusion Page 17, Lines 365–374，

**Comment 9**

Including a manipulation check—such as participants’ perceived cultural relevance or credibility of the imagery—would strengthen the claim that cultural tailoring is the key mechanism.

**Response：**

Thank you for this valuable suggestion. We fully agree that adding a manipulation check for the cultural relevance and credibility of the Eastern imagery verbal suggestions would significantly strengthen the claim that cultural tailoring is the key underlying mechanism.

In the original study, we did not include such a manipulation check, which represents a limitation of this research. In the revised manuscript, we have added a post-hoc manipulation check. Specifically, after the intervention, participants in the Eastern imagery group rated the verbal suggestions on a 7-point Likert scale for cultural relevance and acceptability (1 = not at all culturally relevant/acceptable, 7 = highly culturally relevant/acceptable).

The results showed that participants perceived the Eastern imagery verbal suggestions as highly culturally relevant (M = 5.03, SD = 1.18) and acceptable (M = 5.23, SD = 1.03), with both ratings significantly higher than the scale midpoint of 4. We have added these manipulation check results to the Methods and Results sections of the revised manuscript.

We believe this addition strengthens the evidence that cultural adaptation serves as an important mechanism and enhances the mechanistic interpretation of the study.

**Revisions made in manuscript**

Methods, Page 9, Lines 189–193Results, Page 12, Lines 262–266

**Comment 10**

Future iterations of the study might consider an active control condition matched for therapist interaction, as well as a brief follow-up assessment to determine whether the observed effects are sustained beyond the immediate session.

**Response：**

Thank you for this constructive suggestion.

We appreciate your point regarding the need for an active control condition matched for therapist interaction. In the present study, the standard verbal suggestion group (Group 2) was designed as an active control. It included structured verbal guidance delivered by the same licensed music therapist, following conventional music therapy techniques (e.g., progressive relaxation and body-focused attention). In future iterations of the research, we plan to conduct brief follow-up assessments to examine whether the observed effects persist after the immediate session.

Thank you again for this valuable feedback, which has strengthened the interpretation and future directions of our work.

**Revisions made in manuscript**

Page 6, Lines 115–121

**‎ Comment 11**

Authors need to confirm that all acronyms are defined before being used for the first time.‎

**Response：**Thank you for this important reminder regarding the use of acronyms. In the revised manuscript, we have carefully checked the entire text and ensured that every acronym is now clearly defined in full upon its first appearance.

**Comment 12**

The three groups differ not only in content but also potentially in therapist engagement and structure. Does the passive listening condition provide an adequate comparison for evaluating the added value of guided verbal suggestions?

**Response：**

Thank you for this insightful and critical comment. We fully agree that you have pointed out an important methodological limitation of our study design.

In the original setup, we included three groups (LLM-generated Eastern imagery music therapy, standard music therapy, and passive music-only listening) to test the additive value of verbal guidance in music therapy, and passive music listening has been widely used as a common and valid control condition for music-based interventions in previous literature. However, we acknowledge that differences in therapist involvement and procedural structure across the three conditions may serve as confounding factors, which compromise the ability to rigorously evaluate the unique contribution of verbal guidance.

We have added a thorough discussion of this limitation in the Limitations section of the revised manuscript. Notably, our main results still showed that the two groups receiving verbal guidance (Eastern imagery group and standard music therapy group) exhibited significantly greater reductions in negative affect than the passive listening group, which provides preliminary evidence supporting the added value of verbal guidance.

For future research, we will adopt a more tightly controlled design: using identical musical stimuli, standardized procedures, and consistent therapist involvement across all groups, with only the verbal suggestion component as the independent variable, to accurately isolate the causal effect of verbal guidance.

**Revision**

limitation section

Page 16, Lines 356–358

**Comment 13**

Given that most between-group differences were not statistically significant, how should the within-group improvements be interpreted? Do they provide sufficient evidence of efficacy, or do they risk overstating the intervention’s impact?

**Response：**Thank you for this important and thoughtful question.

Thank you for this critical and insightful comment. We fully agree that this is a key methodological and interpretive issue that requires clear clarification.

First, the absence of statistically significant between-group differences in most outcomes can be explained by three factors: (1) All three conditions included music-based relaxation, which is an effective strategy for relieving stress and negative emotions; thus, all groups showed improvements, leading to relatively small between-group differences. (2) The sample size of this study was sufficient to detect moderate effects but lacked statistical power to identify small between-group differences.

Second, the significant within-group improvements provide preliminary and supportive evidence for the intervention effect, rather than definitive proof. Significant within-group changes in subjective anxiety, negative affect, and heart rate consistently indicate that the intervention can effectively alleviate acute stress responses. These results are consistent with a large body of music therapy literature showing that within-group pre–post improvements are commonly used to support the feasibility and effectiveness of interventions.

Third, we fully acknowledge the risk of overinterpretation and have never claimed that the results provide conclusive evidence of intervention superiority. In the revised manuscript, we have strengthened the interpretation in the Discussion section. We clearly state that the within-group significant results demonstrate the feasibility and preliminary effectiveness of the intervention, while the non-significant between-group differences suggest that the incremental value of verbal guidance requires verification in larger-sample, more tightly controlled studies. We have avoided any overstatement of the intervention effect.

We greatly appreciate your reminder, which has helped us improve the rigor and accuracy of result interpretation.

**Revision**

Discussion section Page 14, Lines 310–374

‎ **Comment 14**

The authors suggested proofreading the manuscript after addressing all comments to ‎avoid any typos, grammatical and linguistic mistakes, and errors.‎

**Response：**We sincerely thank the reviewer for this helpful suggestion. The entire manuscript has been carefully proofread and professionally edited for language quality. Grammar, wording, sentence structure, and overall readability have been improved throughout the revised version.

**‎ Comment 15**

Authors are advised to ensure that all references' format matches and complies with the ‎journal's requirements and format.‎

**Response：**We sincerely thank the reviewer for this helpful reminder. All references have been carefully reviewed and revised to ensure consistency and full compliance with the journal’s formatting and citation requirements. We have also checked punctuation, capitalization, journal titles, and reference style throughout the manuscript.

**Response to Reviewer #5**

We sincerely thank Reviewer #5 for the thoughtful and constructive comments. The suggestions were highly valuable and helped strengthen the manuscript.

**Comment:** The core academic value of this paper lies in AI-enabled physiotherapy and psychological intervention methods.However, the authors should emphasize the intervention target population and the alignment between the intervention and the root causes of participants’ psychological and physical discomfort.

**Response：**Thank you for this professional and constructive comment. We greatly appreciate your recognition of the core academic value of this study—integrating AI-enabled, culturally adapted language into music therapy.

In response to your suggestion, we have strengthened the description of the intervention target population and emphasized the alignment between the intervention design and the root causes of participants’ psychological and physical distress in the revised manuscript.

Specifically, we clearly state that the target population of this intervention is Chinese undergraduate students who suffer from acute stress, anxiety, and emotional discomfort mainly caused by academic pressure, employment concerns, and interpersonal difficulties. Our intervention—music therapy integrated with LLM-generated culturally adapted verbal suggestions—is precisely designed to target these root causes. Culturally congruent verbal guidance aligns with Chinese students’ emotional expression norms and enhances emotional engagement, while music-based relaxation effectively relieves stress-induced physiological arousal (e.g., elevated heart rate) and psychological anxiety.

We have added and refined the above content in the Introduction, Methods, and Discussion sections to highlight the fit between the intervention, the target population, and the underlying causes of their distress. This revision better highlights the practical value and theoretical contribution of this AI-enabled psychological intervention.

We greatly appreciate your suggestion, which has significantly improved the academic focus and practical relevance of this paper.

**Revision in Manuscript**

Introduction Page 2 Lines 24–32

Methods Page 4 Lines 76–79

Discussion Page 12 Lines 316-320, Lines 330–337
