# Supplementary material for: Integrating large language model-generated culturally adapted verbal suggestions into music therapy for university students: A pilot randomized exploratory study
Source: PLoS One. 2026 Oct 9;21(10):e0360478. doi: 10.1371/journal.pone.0360478 (PMC13652252; doi:10.1371/journal.pone.0360478)
Supplement: Supplementary file 5 [file pone.0360478.s006.docx]

**Response to Reviewers**

Dear Editor and Reviewers,

Thank you for the opportunity to revise and resubmit our manuscript. We sincerely appreciate the time, effort, and constructive comments provided by the Editor and all reviewers. Their insightful suggestions were highly valuable and helped improve the clarity, rigor, and overall quality of the manuscript.

We carefully considered every comment and revised the manuscript accordingly. Below, we provide a detailed point-by-point response to each reviewer’s comments. We hope that the revised manuscript meets the journal’s standards and we sincerely appreciate your further consideration.

Sincerely,

The authors

**Response to Reviewer #1**

**Comment：**

The authors have diligently incorporated all feedback provided into the revised version of the manuscript, ensuring that each comment has been adequately addressed and resolved to satisfaction.

**Response**

Thank you very much. We sincerely appreciate your time, constructive comments, and valuable support throughout the review process.

**Response to Reviewer #2**

**Comment：**

All comments have been addressed and authors should be complimented for this. I think it should be accepted.

**Response**

Thank you very much for your positive evaluation of our manuscript and for recommending its publication. We sincerely appreciate your time, constructive comments, and valuable support throughout the review process.

**Response to Reviewer #4**

**Comment：**

The authors have diligently revised and improved the manuscript based on the feedback provided by reviewers during the previous review cycle. As a result of these revisions, the quality of the manuscript now aligns with the standards set by the journal. However, upon thorough examination of the manuscript, it becomes evident that there are certain linguistic and grammatical issues present throughout. To address these concerns, the authors are strongly advised to have the manuscript proofread by a native English speaker. This step will help rectify any lingering linguistic or grammatical errors and ensure that the manuscript reads fluently and coherently. Additionally, it is imperative for the authors to meticulously review and confirm that all references cited in the manuscript adhere to the journal's prescribed referencing style and format. Ensuring consistency and accuracy in reference formatting is crucial for maintaining the integrity and professionalism of the manuscript. By taking these measures to address language and referencing issues, the authors can further enhance the clarity, readability, and overall quality of their manuscript, thereby facilitating a smoother publication process and better dissemination of their research findings.

**Response**

Thank you for your valuable suggestion. To further improve the linguistic quality and readability of the manuscript, we invited two individuals with relevant expertise to carefully proofread and revise the manuscript. One is a native English speaker, and the other is a PhD in English linguistics. The manuscript has been thoroughly revised based on their feedback. In addition, we carefully rechecked all references to ensure that they comply with the journal's requirements.

**Response to Reviewer #6**

This paper tests whether verbal suggestions written by ChatGPT and framed around Chinese cultural imagery can make music therapy more effective at reducing anxity in university students. The idea is genuinely novel and practically relevant, and the study uses a proper randomized controlled trial design with three groups, measuring anxiety and heart rate before and after the intervention. At first glance, it reads as a competent piece of applied research. However, the paper has a fundamental problem that must be addressed before this work can move forward.

**Comment 1.** The central claim that "LLM-generated, culturally adapted verbal suggestions produced better outcomes" is simply not supported by the data. The main statistical test, the between-group comparison, shows no significant differences across any primary outcome. The authors pivot to within-group improvements as their main finding, but that is not what an RCT is designed to demonstrate. All three groups got better, and the study cannot tell us why. The title, abstract, and conclusion must be rewritten to reflect this honestly. The paper can and should be reframed as a pilot feasibility study, which is a legitimate and publishable contribution - but the current framing constitutes overclaiming and does not meet the standard that conclusions be drawn appropriately from the data presented.

**Response**

Thank you for this important and constructive comment. We agree with your assessment that, given the absence of statistically significant between-group differences and the methodological limitations of the current design, the study is more appropriately framed as a pilot feasibility study rather than as a definitive evaluation of intervention effectiveness.

In response to your observation, we have revised the title to more accurately reflect the exploratory nature and methodological scope of the study. The manuscript is now explicitly framed as a pilot randomized feasibility study rather than a confirmatory effectiveness trial. Corresponding revisions have also been made throughout the Abstract, research aims, Discussion, and Conclusion to avoid causal, confirmatory, or efficacy-related claims that are not fully supported by the data.

You also noted that “All three groups got better, and the study cannot tell us why.” We agree with this concern and have addressed it explicitly in the revised Discussion. One possible explanation is that the musical materials themselves, including their melodic, stylistic, and emotional characteristics, may have contributed to the observed reductions in anxiety. We sincerely appreciate your suggestion, which has helped us substantially improve the framing and interpretation of the study.

**Revisions made in manuscript**

The Title, Abstract, Methods, Discussion, and Conclusion sections have been revised.

**Comment 2.** Beyond the null result, the study design has a confound that runs through every conclusion the authors draw. Each group listened to completely different music - Chinese film music, a clinical therapy recording, and Japanes film music, composed by different people, in different styles, with different emotional characters, and with run times that vary by over four minutes. Trying to isolate the effect of verbal suggestion content when the music itself is this different is not possible. Any outcome difference could be due to the music, the words, familiarity with the soundtrack, the emotional tone of the piece, or any combination of these. This is acknowledged as a limitation in the manuscript, but it is actually a design flaw that prevents the key comparison from being made. The authors should explicitly state this in the discussion, rather than treating it as a minor caveat.

**Response**

Thank you very much for identifying this important design issue. We agree that the use of different musical materials across the three experimental conditions represents an important methodological limitation of the study design.

In response to this concern, we first expanded the *Discussion* to explain one possible reason why no significant between-group differences in anxiety reduction were observed. Second, in the Limitations, we explicitly acknowledge that the use of different musical materials across conditions constitutes an important confounding factor. We also suggest that future studies should use identical musical materials across all conditions to allow a more accurate evaluation of the effects of different verbal suggestions.

At the same time, we would like to further clarify the rationale behind our original design. Music itself carries cultural characteristics, and music from different cultural backgrounds may convey different aesthetic imagery and cultural meanings. Therefore, we intentionally paired culturally similar musical materials with the corresponding verbal suggestions to enhance the cultural consistency of the intervention content. Nevertheless, we fully recognise that, although this design helped preserve the cultural integrity of each intervention condition, it also limited our ability to isolate the independent effect of the verbal suggestions.

Although no significant between-group differences were observed, we sincerely appreciate your recognition of the value of this study as a pilot and exploratory investigation. We are currently continuing this line of research and have already adopted a revised design in a follow-up study, in which all groups receive identical musical materials while only the verbal suggestions differ. This will allow us to evaluate the unique contribution of culturally adapted verbal suggestions more rigorously. Once again, we sincerely thank you for your valuable comments and suggestions.

**Revisions made in manuscript**

Discussion: p. 15, lines 315–325;
Study Limitations: p. 17, lines 366–370.

**Comment 3.** There are also concerns about the two references added in this revision, References 55 and 56. These papers are about aortic stenosis detection and breast cancer imaging using deep learning. They have no relevance to music therapy, cultural adaptation, or anxiety research. Are these references genuinely necessary?

**Response**

Thank you for your suggestion. We have removed the two references (formerly References 55 and 56) identified in your comment, together with the corresponding citations in the manuscript. Please note that the current References 55 and 56 are newly added sources that support our discussion of the potential confounding effects arising from differences in the musical materials used across the intervention groups.

**Comment 4.** Several other issues compound the concern. The Cultural Resonance Manipulation Check was added after data collection in response to reviewer comments and was only administered to one group - without comparative data from the other two groups, it cannot establish that the Eastern imagery sugestions were perceived as more culturally resonant than standard suggestions, only that participants approved of them. It must be labeled post-hoc and exploratory, and the causal language around it must be removed.

**Response**

Thank you for raising this important point. We agree that the assessment of perceived cultural resonance was conducted after the completion of the primary data collection in response to the reviewers' comments.

Following your suggestion, we have removed the term **"manipulation check"** and replaced it with **"Post-hoc Exploratory Assessment of Perceived Cultural Resonance."** We have removed all causal language and revised the description to indicate that the findings **provide preliminary descriptive evidence** rather than supporting causal inferences.

Once again, we sincerely thank you for this valuable suggestion.

**Revisions made in manuscript**

Methods: p. 9, lines 195–201;
Results: p. 12, lines 268–273.

**Comment 5.** The sample is 94% women, with no sex-stratified analysis and no discussion of what this means for generalizability.

**Response**

Thank you for pointing out this important limitation. We acknowledge that the sample was predominantly female, with 94 female and 19 male participants among the total sample of 113 participants. Accordingly, we have added this limitation to the manuscript and recommended that future studies recruit more gender-balanced samples. You also suggested conducting gender-stratified analyses. However, because the number of male participants was relatively small (n = 19), further stratification by experimental condition would have resulted in very small subgroup sizes and limited statistical power. Therefore, gender-stratified analyses were not performed in the present study.

Once again, we sincerely thank you for your valuable comments and suggestions.

**Revisions made in manuscript**

Limitations: p. 17, lines 363–366.

**Comment 6.** The correlation analysis reports n = 115 when only 113 participants were enrolled - this discrepancy must be identified and corrected.

**Response**

Thank you for pointing out this important data consistency issue.

We carefully rechecked the original dataset and the statistical analysis outputs and confirmed that all analyses were conducted using data from **113 participants**. We have corrected this error throughout the manuscript. Thank you very much for bringing it to our attention.

**Revisions made in manuscript**

Results (Objective Anxiety Measurement *[Heart Rate]*): p. 13, line 278.

**Comment 7.** The stress induction task has no validated manipulation check confirming it actually elevated anxiety.

**Response**

Thank you for raising this important point.

We agree that the present study could not directly verify whether the 40-minute cognitive task successfully increased participants' anxiety levels. We have acknowledged this as a limitation in the Discussion and noted that future studies should assess participants' state anxiety both before and after the stress induction task to verify the effectiveness of the anxiety induction procedure.

Once again, we sincerely thank you for this valuable suggestion.

**Revisions made in manuscript**

Procedure: p. 7, lines 146–149;
Limitations: p. 17, lines 370–374.

**Comment 8.** So, the paper addresses an interesting question, and with honest reframing and thorough revision it can make a real contribution to this emerging area. But in its current form, the evidence does not support the conclusions being drawn, and the design cannot adequately test the core hypothesis. Major structural revision is required before this can be considered for publication.

**Response**

We sincerely thank you for your comprehensive and constructive evaluation of our manuscript. In response to your comments, we have undertaken substantial structural revisions. The revisions include:

1. Reframing the study as a **pilot randomized feasibility study**, rather than a definitive evaluation of intervention effectiveness.
2. Revising the Title (p. 1, lines 1–4), Abstract (p. 2, lines 13–25), Research Aims (p. 4, lines 63–71), Discussion (p. 15, lines 311–329; p. 17, lines 363–376), and Conclusion (p. 17, lines 378–386) to ensure that the claims are appropriately aligned with the level of evidence provided by the data.
3. Explicitly acknowledging the limitation arising from the use of different musical materials across the three intervention conditions and recommending that future studies use identical musical materials across conditions.
4. Avoiding overstatement of the effectiveness of LLM-assisted music-based interventions.
5. Removing irrelevant references.
6. Correcting the reported sample size.
7. Acknowledging the limitation of the unbalanced gender distribution.

We sincerely thank you again for your thoughtful and constructive comments, which have helped us substantially improve the quality and clarity of our manuscript.

**Response to Reviewer #7**

**Comment：**

The revisions and the Authors’ Response are quite good and generally acceptable, especially since the authors have substantively addressed most of the key methodological issues. The manuscript has provided clarifications regarding music segmentation, randomization and blinding procedures, LLM prompts, cultural manipulation, heart rate measurements, sensitivity analysis for outliers, and acknowledged limitations related to statistical power, non-identical intervention durations, differences in musical stimuli, and potential confounding by therapist or demand characteristics. However, this acceptance should be viewed with caution. The article’s contribution remains stronger as a preliminary/exploratory study rather than conclusive evidence of efficacy, as many between-group results were not significant, the power analysis was conducted post hoc/as a sensitivity analysis, and some methodological controls are still not ideal. Therefore, the authors’ response is “acceptable” for proceeding with publication, provided that the claims in the abstract, discussion, and conclusions are toned down to avoid overstating the effectiveness of the LLM-assisted music therapy intervention.

**Response**

Thank you for this important comment. Accordingly, we have substantially revised the manuscript by reframing the study as a **pilot randomized feasibility study** rather than a definitive evaluation of intervention effectiveness.

We have also made corresponding revisions to the Title (p. 1, lines 1–4), Abstract (p. 2, lines 13–25), Research Aims (p. 4, lines 63–71), Discussion (p. 15, lines 311–329; p. 17, lines 363–376), and Conclusion (p. 17, lines 378–386) to clearly position the study as a preliminary and exploratory investigation. Throughout the revised manuscript, we have avoided overstating the effectiveness of the LLM-assisted music-based intervention and have ensured that the conclusions remain consistent with the evidence provided by the data.

Once again, we sincerely thank you for this valuable suggestion, which has helped us improve the scientific rigor and overall presentation of the manuscript.
