# Supplementary material for: Integrating large language model-generated culturally adapted verbal suggestions into music therapy for university students: A pilot randomized exploratory study
Source: PLoS One. 2026 Oct 9;21(10):e0360478. doi: 10.1371/journal.pone.0360478 (PMC13652252; doi:10.1371/journal.pone.0360478)
Supplement: Supplementary file 6 [file pone.0360478.s007.docx]

**Response to Editor and Reviewers**

Dear Editor and Reviewers,

Thank you for the opportunity to revise and resubmit our manuscript. We sincerely appreciate the time, effort, and constructive comments provided by the Editor and all reviewers. Their insightful suggestions were highly valuable and helped improve the clarity, rigor, and overall quality of the manuscript.

We carefully considered every comment and revised the manuscript accordingly. Below, we provide a detailed point-by-point response to each reviewer’s comments. We hope that the revised manuscript meets the journal’s standards and we sincerely appreciate your further consideration.

Sincerely,

The authors

**Comment 1.**

The manuscript presents several statistical inconsistencies that would benefit from careful reconciliation. The Methods state that heart rate data were not normally distributed and would be analysed using non-parametric methods, yet the Results present paired t-tests for heart rate. Additionally, line 280 refers to the between-group analysis as a "one-way ANOVA," whilst the Methods, the Dunn's post-hoc test, and Table 3 all indicate that a Kruskal-Wallis test was employed. More concerning is the discrepancy between the main analysis, which reports a significant between-group difference in heart rate change (p = .02), and the sensitivity analysis, which states that all comparisons, including heart rate, were non-significant. Given that no heart rate outliers were removed, these analyses should logically operate on the same dataset;

- Furthermore, the same t-value (t = 2.07) is reported with p = .03 in the main analysis and p = .046 in the sensitivity analysis, which appears mathematically inconsistent. Finally, there are mismatches between the narrative text and tables. The text reports p = .07 for negative affect at baseline whilst Table 2 shows p = .10. Similarly, for heart rate, the text reports p = .43 whilst Table 3 presents p = .48. The authors might consider verifying these values against the raw data and clarifying which figures are correct, or providing an explanation for any genuine differences between analyses;

**Response**

We thank the reviewer for identifying these inconsistencies. In response to this comment and Comment 3 below, we revised the primary analytical approach by replacing the between-group comparisons of change scores with baseline-adjusted analyses of covariance (ANCOVAs). Accordingly, all relevant statistical results, tables, and narrative descriptions were recalculated and revised. The revised manuscript now reports adjusted means with 95% confidence intervals, omnibus F tests, partial eta squared (η*_p_*²), and Holm-adjusted pairwise comparisons. We appreciate the reviewer’s helpful recommendation.

Regarding heart rate, although the raw scores were not normally distributed, the ANCOVA residuals were approximately normally distributed (Shapiro–Wilk test, p = .719), the residual variances did not differ significantly across groups (Levene’s test, p = .073), and the homogeneity-of-regression-slopes assumption was satisfied (group × baseline interaction, p = .412). We therefore used ANCOVA consistently as the primary analysis for heart rate.

The reviewer was also correct regarding the paired-samples test for state anxiety. The correct result was t(35) = 2.07, p = .046 (two-sided). Re-examination of the previous analyses showed that the baseline ANOVA result for negative affect should have been p = .07. For heart rate, the ANOVA result should have been p = .43, whereas the Kruskal–Wallis result should have been p = .48. However, because the revised manuscript now uses baseline-adjusted ANCOVA as the primary between-group analysis, these superseded analyses and values have been removed.

Finally, the corrected primary and sensitivity analyses are now reported consistently. No heart-rate observation met the prespecified model-outlier criterion; consequently, the primary and outlier-based sensitivity datasets were identical, and the numerical results remained unchanged.

**Revisions made in manuscript**

Methods—Data Analysis, p. 9, lines 206–222;

Results—p. 10, lines 224–260; Tables 2–4.

Sensitivity Analyses, p. 13, lines 271–281, and all relevant statements in the Abstract and Discussion.

**Comment 2.**

This study is described as a "feasibility study," yet no feasibility outcomes have been formally defined or reported. Standard feasibility metrics such as recruitment rate, retention, intervention completion, adherence, protocol fidelity, acceptability, and adverse events are absent. Additionally, any pre-specified progression criteria that would support a transition to a definitive trial have not been provided. The post-hoc cultural evaluation, conducted in a single group, is insufficient to substantiate a claim of feasibility on its own;

- The authors might consider two options moving forward. One possibility would be to strengthen the feasibility framework by explicitly defining and reporting these outcomes in the Results section, along with clear progression criteria for a future trial. Alternatively, the study could be reframed more modestly as a "pilot randomised exploratory study" if a comprehensive feasibility assessment is not feasible given the data already collected. In either case, statements asserting that feasibility was "confirmed" or "demonstrated" would benefit from softer language that better reflects what the data actually show;

**Response**

We agree with the reviewer. We have reframed the study as a “pilot randomized exploratory study” and revised the title accordingly: “Integrating Large Language Model-Generated Culturally Adapted Verbal Suggestions into Music Therapy for University Students: A Pilot Randomized Exploratory Study.” Statements claiming that feasibility was “confirmed” or “demonstrated” have been removed.

**Revisions made in manuscript**

Title; p.1

Abstract, p. 2, line 14;

Methods—p. 4, lines 79–80;

Discussion, p. 14, lines 295–296;

Conclusion, p. 17, lines 370–375.

**Comment 3.**

Although the Title and abstract have been revised, the body of the manuscript contains several claims that exceed what the data support. These include "indicating the effectiveness of this intervention," "supports the functional role of verbal guidance," "potential advantage of culturally adapted language," "broadly applicable regulatory effect," and "feasible and promising." These phrases might be reconsidered in favour of more measured language such as "suggesting a possible effect that warrants further investigation" or "an effect that may warrant testing in broader populations," which better reflect the exploratory nature of the work;

- More importantly, the change in negative affect did not differ significantly between groups (p = .52), yet the discussion implies a between-group advantage based on a post-test difference alone. This is problematic given that the groups differed at baseline, meaning any post-test difference may reflect pre-existing disparities rather than a treatment effect. The authors might explore whether reanalysing this outcome using an ANCOVA or a group by time interaction model that accounts for baseline differences would alter the interpretation, or alternatively, consider whether the interpretive claim of a between-group advantage should be reconsidered in light of these baseline differences.

**Response**

We agree with the reviewer and have moderated the relevant claims throughout the Results, Discussion, and Conclusion. Specifically, we removed or revised the following expressions: “indicating the effectiveness of this intervention,” “supports the functional role of verbal guidance,” “potential advantage of culturally adapted language,” “broadly applicable regulatory effect,” and “feasible and promising.” The revised manuscript describes the findings as possible differences between intervention conditions that require confirmation in a larger-scale trial.

Following the reviewer’s recommendation, we reanalysed all available data using ANCOVA. The post-intervention negative-affect score as the dependent variable and the baseline negative-affect score as the covariate. The baseline-adjusted group effect was statistically significant, F(2, 109) = 4.60, p = .012, η_p_² = .078. Pairwise comparisons were conducted with Holm correction. This analysis replaces the previous interpretation based on unadjusted comparisons.

**Revisions made in manuscript**

**Page 2, lines 24–27:**
“The findings identify potentially meaningful response patterns but do not establish efficacy or the independent effect of culturally adapted verbal suggestions because the conditions differed in musical material and duration. Larger, controlled trials are required.”

**Page 14, lines 306–308:**
“The absence of a significant adjusted group effect therefore provides no evidence that the Eastern imagery condition reduced state anxiety more than the standardized therapy or music-only conditions.”

**Page 15, lines 321–324:**
“Nevertheless, the result cannot be attributed specifically to cultural adaptation or LLM generation. The Eastern imagery condition combined a distinctive musical repertoire with culturally framed language, whereas the comparison conditions differed on several components. The finding should therefore be regarded as a possible signal warranting confirmatory testing.”

**Page 16, lines 344–345:**
“This null finding should not be interpreted as evidence that the intervention is broadly applicable across trait-anxiety levels.”

**Page 16, lines 346–347:**
“The subgroup findings are therefore descriptive and require examination in a larger sample with prospectively defined strata or a continuous moderation analysis.”

**Page 17, lines 373–375:**
“These findings should be interpreted with caution: the intervention conditions differed in musical material and duration, so the independent effects of the LLM-generated, culturally adapted suggestions could not be isolated.”

**Comment 4.**

Overall, the manuscript addresses a timely and interesting question, and the effort to integrate LLM-generated culturally adapted verbal suggestions into music therapy is commendable. Addressing the statistical and conceptual points raised above would meaningfully strengthen the rigour and credibility of the study, and the authors are encouraged to revise the manuscript with these considerations in mind.

**Response**

Finally, we would like to express our sincere gratitude to the editor and reviewer for their careful and patient identification of the study’s limitations and for their valuable recommendations. We particularly appreciate your recognition of our efforts to integrate LLM-generated, culturally adapted verbal suggestions into music therapy. After addressing the limitations of the present study, we intend to continue developing this line of research.
