# Supplementary material for: Integrating large language model-generated culturally adapted verbal suggestions into music therapy for university students: A pilot randomized exploratory study
Source: PLoS One. 2026 Oct 9;21(10):e0360478. doi: 10.1371/journal.pone.0360478 (PMC13652252; doi:10.1371/journal.pone.0360478)
Supplement: Peer review history

# Peer review: Decision Letter 0

**DOI:** 10.1371/journal.pone.0360478.r001

**Authors:** Thiago P. Fernandes

13 Mar 2026

Dear Dr. Li,

Please submit your revised manuscript by Apr 27 2026 11:59PM. If you will need more time than this to complete your revisions, please reply to this message or contact the journal office at plosone@plos.org. When you're ready to submit your revision, log on to https://www.editorialmanager.com/pone/ and select the 'Submissions Needing Revision' folder to locate your manuscript file.

- A letter that responds to each point raised by the academic editor and reviewer(s). You should upload this letter as a separate file labeled 'Response to Reviewers'.
- A marked-up copy of your manuscript that highlights changes made to the original version. You should upload this as a separate file labeled 'Revised Manuscript with Track Changes'.
- An unmarked version of your revised paper without tracked changes. You should upload this as a separate file labeled 'Manuscript'.

If you would like to make changes to your financial disclosure, please include your updated statement in your cover letter. Guidelines for resubmitting your figure files are available below the reviewer comments at the end of this letter.

If applicable, we recommend that you deposit your laboratory protocols in protocols.io to enhance the reproducibility of your results. Protocols.io assigns your protocol its own identifier (DOI) so that it can be cited independently in the future. For instructions see: https://journals.plos.org/plosone/s/submission-guidelines#loc-laboratory-protocols. Additionally, PLOS ONE offers an option for publishing peer-reviewed Lab Protocol articles, which describe protocols hosted on protocols.io. Read more information on sharing protocols at https://plos.org/protocols?utm_medium=editorial-email&utm_source=authorletters&utm_campaign=protocols.

We look forward to receiving your revised manuscript.

Kind regards,

Thiago P. Fernandes, PhD

Academic Editor

PLOS One

Journal Requirements:

When submitting your revision, we need you to address these additional requirements.

1. Please ensure that your manuscript meets PLOS ONE's style requirements, including those for file naming. The PLOS ONE style templates can be found at

https://journals.plos.org/plosone/s/file?id=wjVg/PLOSOne_formatting_sample_main_body.pdf and

https://journals.plos.org/plosone/s/file?id=ba62/PLOSOne_formatting_sample_title_authors_affiliations.pdf

2. We note that the grant information you provided in the ‘Funding Information’ and ‘Financial Disclosure’ sections do not match.

When you resubmit, please ensure that you provide the correct grant numbers for the awards you received for your study in the ‘Funding Information’ section.

3. Thank you for stating the following financial disclosure:

“This work was supported by the 2026 Aba Teachers College Scientific Research Innovation Team Fund (Grant No. AS-KCTD2026-18), Research on Teachers’ Development and Mental Health in Ethnic Areas of Sichuan, and by the Aba Teachers College Talent Development Special Project, Research on Artificial Intelligence–Empowered Lifelong Learning Mechanisms for Teachers in Ethnic Regions (Grant No. AS-RCZX2026-45).”

Please state what role the funders took in the study.  If the funders had no role, please state: "The funders had no role in study design, data collection and analysis, decision to publish, or preparation of the manuscript." If this statement is not correct you must amend it as needed.

Please include this amended Role of Funder statement in your cover letter; we will change the online submission form on your behalf.

4. Please include captions for your Supporting Information files at the end of your manuscript, and update any in-text citations to match accordingly. Please see our Supporting Information guidelines for more information: http://journals.plos.org/plosone/s/supporting-information.

5. If the reviewer comments include a recommendation to cite specific previously published works, please review and evaluate these publications to determine whether they are relevant and should be cited. There is no requirement to cite these works unless the editor has indicated otherwise.

**Additional Editor Comments:**

Please respond to all comments and highlight the changes in the revised manuscript.

[Note: HTML markup is below. Please do not edit.]

Reviewers' comments:

Reviewer's Responses to Questions

**Comments to the Author**

1. Is the manuscript technically sound, and do the data support the conclusions?

Reviewer #1: Yes

Reviewer #2: Yes

Reviewer #3: Yes

Reviewer #4: Yes

Reviewer #5: Yes

**********

2. Has the statistical analysis been performed appropriately and rigorously?

Reviewer #1: No

Reviewer #2: Yes

Reviewer #3: Yes

Reviewer #4: Yes

Reviewer #5: Yes

**********

3. Have the authors made all data underlying the findings in their manuscript fully available?

The PLOS Data policy

Reviewer #1: Yes

Reviewer #2: Yes

Reviewer #3: Yes

Reviewer #4: Yes

Reviewer #5: Yes

**********

4. Is the manuscript presented in an intelligible fashion and written in standard English?

Reviewer #1: No

Reviewer #2: Yes

Reviewer #3: Yes

Reviewer #4: Yes

Reviewer #5: Yes

**********

Reviewer #1: Dear Authors

The manuscript titled "The Effectiveness of Music Therapy Incorporating Culturally Adapted Verbal Suggestions Generated by Large Language Models: A Randomized Controlled Trial" addresses important issues regarding culturally adapted interventions and AI integration in music therapy; however, it needs major improvements based on the given comments.

1. Authors should clarify how the 30 minute soundtrack from Hero was segmented and whether the verbal suggestions were timed to align with specific musical passages.Please see and add [1] Generation of Voice Signal Tone Sandhi and Melody Based on Convolutional Neural Network.

2. How does the study control for the confounding variable of therapist presence, given that only the two verbal suggestion groups involved a live therapist reading scripts?

3. Please explain why heart rate data violated normality assumptions and whether non parametric tests were appropriately applied to change scores rather than raw values.

4. Authors should justify the use of a 40 minute examination task for stress induction without reporting manipulation checks or post induction baseline measures.

5. How does the study ensure that the "standard verbal suggestions" represent actual clinical practice rather than an artificially constructed comparator?

6. Please provide inter rater reliability data for the therapist's delivery of verbal suggestions to ensure consistency across participants within groups.

7. Authors should report a priori power analysis and explain whether the sample size of 113 participants was sufficient to detect expected effect sizes.

8. How does the study address potential demand characteristics, given that participants in the Eastern imagery group might guess the cultural tailoring hypothesis?

9. Please clarify why the PANAS positive affect showed no changes whatsoever, and whether ceiling or floor effects might explain this null finding.

10. Authors should explain how the eight musical pieces from Kikujiro were selected and whether their joyful character might confound comparisons with the other groups.

11. How does the study account for participants' prior familiarity with or emotional associations to the film soundtracks used as stimuli?

12. Please provide the full ChatGPT prompt in the methods section, including any temperature settings or other parameters that might affect output reproducibility.

13. Authors should justify the exclusion of outliers from STAI and PANAS data while retaining all heart rate data, and report sensitivity analyses with outliers included.

14. How does the study ensure that the Eastern imagery verbal suggestions actually reflect culturally adapted content rather than generic poetic language?

15. Authors should discuss why the future scope should contain explanability and newer transformer models, please see and add [2] Tri-STANet: An advanced framework integrating dual axis transformer and morphological encoding for enhanced AS detection; [3] XAI-RACapsNet: Relevance aware capsule network-based breast cancer detection using mammography images via explainability O-net ROI segmentation

16. Please explain why trait anxiety moderation analyses were underpowered, with only seven participants in the low trait anxiety subgroup of the Eastern imagery condition.

17. Authors should clarify whether heart rate measurements were taken immediately post intervention or after a delay, and whether room conditions were standardized.

18. How does the study address the multiple comparison problem given the numerous t tests and ANOVAs performed without correction for family wise error rate?

19. Please provide the exact duration of each intervention and confirm that exposure time was precisely equivalent across all three experimental conditions.

Reviewer #2: Interesiting paper Some issues should be added

Introduction should be shorted to maximun 1 page and a half

methods: it should be added who adjudicated the quality of life

methods: it is not clear if authors used block sizes for the rct

methods: sample size calculation should be improved

methods/results: do authors think that embedding multivariate analysis may increase the power of the analysis?

Reviewer #3: This study investigates the integration of large language model–generated, culturally adapted verbal suggestions into music therapy for anxiety reduction among Chinese undergraduates, comparing it against standard verbal suggestions and passive music listening in a randomized controlled trial.

(1) The sample size appears underpowered to detect between-group differences, especially given the small effect sizes reported and the use of three groups without a clear a priori power calculation.

(2) It’s unclear why different film soundtracks were used across the three conditions.

(3) The stress induction task lasted 40 minutes but is described only vaguely; without details on cognitive load or validation of its effectiveness, its role as a standardized stressor remains questionable.

(4) Heart rate was measured using a smartphone app, yet no information is provided about calibration, participant positioning, or consistency of measurement technique across individuals.

(5) The Eastern imagery script uses poetic metaphors, but there’s no evidence that participants actually perceived these as culturally resonant or more acceptable than standard suggestions.

(6) Trait anxiety was used to stratify a subgroup analysis, but the low-anxiety group had only seven participants, making any comparison statistically unreliable and possibly misleading.

(7) Although ChatGPT 5.2 is named, there’s no description of prompt engineering, temperature settings, or how output consistency was ensured across potential multiple generations.

(8) The claim that LLM-generated content is “culturally adaptive” rests solely on researcher interpretation rather than empirical validation from cultural experts or target-user feedback.

(9) Passive music listening used Joe Hisaishi’s compositions, which are emotionally evocative—this may not serve as an inert control, potentially diluting observed differences between active and passive conditions.

(10) Outlier removal followed IQR rules, but with small group sizes (e.g., n=36), excluding even two outliers could disproportionately influence mean estimates and p-values.

(11) The correlation between heart rate and state anxiety change is modest yet it’s presented as meaningful support for physiological–subjective alignment without considering alternative explanations like regression to the mean.

Reviewer #4: This manuscript explores whether culturally tailored language generated by a large language model can enhance the verbal suggestion component of music therapy. In a randomized controlled trial involving 113 Chinese university students, participants were assigned to one of three conditions: music therapy with AI-generated culturally relevant imagery, standard music therapy with neutral suggestions, or passive music listening. The study assessed changes in anxiety, affect, and heart rate before and after a stress task and intervention session. The authors report that participants receiving the AI-supported intervention showed reductions in state anxiety and negative affect within the group, and changes in anxiety were associated with physiological measures. Although differences between groups were limited, the study provides an innovative and methodologically transparent attempt to integrate AI-generated content into established therapeutic practice, offering a useful foundation for further research in culturally sensitive, technology-assisted interventions. The authors suggested addressing the following comments and suggestions when preparing ‎the revised version:‎

‎= Abstract: The section needs to be re-drafted to be self-contained means it has to clearly ‎show the hypothesis, methodology, techniques, and tools used, and the results obtained.‎

‎= Keywords: The Authors suggested updating the keywords by selecting more relevant terms. ‎Keywords play an important role in the appearance of the manuscript in scholars' searches, ‎which will give it more hits and more citations.‎

= The manuscript would benefit from more detailed reporting of how the AI-generated scripts were produced and standardized. What specific prompts and parameters were used, and how was consistency ensured across participants?

= Could the authors clarify the procedures for randomization, allocation, and blinding? Additionally, were participants aware that some scripts were AI-generated, and if so, might this have influenced outcomes?

‎= Introduction: The authors advised adding one more paragraph at the end of the section to ‎show the organization of the rest of the paper.‎

‎= The study suggests that culturally tailored, AI-generated imagery may help reduce state anxiety. However, is the observed improvement truly due to cultural resonance, or could it reflect expectancy effects, novelty of the AI component, or therapist influence during delivery?

= Should AI-generated verbal suggestions be viewed as an active therapeutic mechanism in their own right, or are they simply tools that depend heavily on how clinicians implement and contextualize them?

‎= Conclusion: The conclusion should be abstracted, so the authors should consider re-‎drafting it.‎

= Including a manipulation check—such as participants’ perceived cultural relevance or credibility of the imagery—would strengthen the claim that cultural tailoring is the key mechanism.

= Future iterations of the study might consider an active control condition matched for therapist interaction, as well as a brief follow-up assessment to determine whether the observed effects are sustained beyond the immediate session.

‎= Authors need to confirm that all acronyms are defined before being used for the first time.‎

= The three groups differ not only in content but also potentially in therapist engagement and structure. Does the passive listening condition provide an adequate comparison for evaluating the added value of guided verbal suggestions?

= Given that most between-group differences were not statistically significant, how should the within-group improvements be interpreted? Do they provide sufficient evidence of efficacy, or do they risk overstating the intervention’s impact?

‎= The authors suggested proofreading the manuscript after addressing all comments to ‎avoid any typos, grammatical and linguistic mistakes, and errors.‎

‎= Authors are advised to ensure that all references' format matches and complies with the ‎journal's requirements and format.‎

Reviewer #5: The core academic value of this paper lies in AI-enabled physiotherapy and psychological intervention methods. However, the authors should emphasize the intervention target population and the alignment between the intervention and the root causes of participants’ psychological and physical discomfort.

**********

what does this mean?). If published, this will include your full peer review and any attached files.

If you choose “no”, your identity will remain anonymous but your review may still be made public.

**Do you want your identity to be public for this peer review?**  For information about this choice, including consent withdrawal, please see our Privacy Policy

Reviewer #1: **Yes:**  Mohd Anul Haq

Reviewer #2: **Yes:**  Fabrizio D'Ascenzo

Reviewer #3: No

Reviewer #4: **Yes:**  Jafar A. Alzubi

Reviewer #5: No

**********

[NOTE: If reviewer comments were submitted as an attachment file, they will be attached to this email and accessible via the submission site. Please log into your account, locate the manuscript record, and check for the action link "View Attachments". If this link does not appear, there are no attachment files.]

To ensure your figures meet our technical requirements, please review our figure guidelines: https://journals.plos.org/plosone/s/figures

You may also use PLOS’s free figure tool, NAAS, to help you prepare publication quality figures: https://journals.plos.org/plosone/s/figures#loc-tools-for-figure-preparation.

NAAS will assess whether your figures meet our technical requirements by comparing each figure against our figure specifications.

# Author comment: Author response to Decision Letter 1

**DOI:** 10.1371/journal.pone.0360478.r002

27 Apr 2026

Response to Reviewers

Manuscript Title: The Effectiveness of Music Therapy Incorporating Culturally Adapted Verbal Suggestions Generated by Large Language Models: A Randomized Controlled Trial

Dear Editor and Reviewers,

Thank you for the opportunity to revise and resubmit our manuscript. We sincerely appreciate the time, effort, and constructive comments provided by the Editor and all five reviewers. Their insightful suggestions were highly valuable and helped improve the clarity, rigor, and overall quality of the manuscript.

We carefully considered every comment and revised the manuscript accordingly. Below, we provide a detailed point-by-point response to each reviewer’s comments. We hope that the revised manuscript meets the journal’s standards and we sincerely appreciate your further consideration.

Sincerely,

The authors

Response to Reviewer #1

We sincerely thank Reviewer #1 for the thoughtful and constructive comments. The suggestions were highly valuable and helped strengthen the manuscript.

Comment 1

Authors should clarify how the 30 minute soundtrack from Hero was segmented and whether the verbal suggestions were timed to align with specific musical passages.Please see and add [1] Generation of Voice Signal Tone Sandhi and Melody Based on Convolutional Neural Network.

Response

We have reviewed and cited the references [34] you recommended, and we sincerely appreciate your suggestions.

In the present study, the 30-minute soundtrack was segmented into seven relatively independent musical sections (each approximately 3–5 minutes), based on the original compositional structure of the film score. These sections are titled: Hero Overture, For the World, Warriors, Longing, Swift Sword, Yearning for the Peace, and Farewell Hero.

The verbal suggestions were not fully aligned with all musical sections; instead, they were selectively embedded within four sections. Specifically, these sections correspond to key emotional phases of the intervention, including emotional engagement (For the World), tension and release (Warriors and Swift Sword), and emotional integration and relaxation (Yearning for the Peace). In addition, these segments exhibit clear musical structures and smooth emotional transitions. Following multiple rounds of discussion between the researchers and certified music therapists, it was determined that embedding verbal suggestions within these sections would not disrupt the listener’s immersive experience.

Revisions made in manuscript

Page 5, Lines 99–106，Update the list of references at the same time.

Comment 2. How does the study control for the confounding variable of therapist presence, given that only the two verbal suggestion groups involved a live therapist reading scripts?

Response

Thank you for this important comment. We acknowledge that therapist presence may represent a potential confounding variable. To minimize this effect, several standardization procedures were implemented. First, the same therapist conducted all intervention sessions across all groups, thereby eliminating variability arising from individual differences between therapists. Second, procedural interactions (e.g., instructions, monitoring, and session management) were kept consistent across all experimental conditions to control for non-specific interpersonal effects.

Revision in Manuscript

Methods section Procedure, Page 6, Lines 133–139

Comment 3. Please explain why heart rate data violated normality assumptions and whether non parametric tests were appropriately applied to change scores rather than raw values.

Response

Thank you for this important comment.

Regarding the violation of normality assumptions, heart rate data were assessed using the Shapiro–Wilk test prior to analysis and were found to deviate from a normal distribution. This pattern is consistent with established findings in psychophysiological research, which indicate that heart rate, as a physiological measure regulated by the autonomic nervous system, often exhibits non-linear dynamics and skewed distributions (Berntson et al., 1997).

Accordingly, group differences in heart rate changes were analyzed using the Kruskal–Wallis test based on change scores rather than raw heart rate values. Where applicable, post hoc pairwise comparisons were conducted using Dunn’s test with adjusted p-values. This approach ensured that the analyses specifically reflected intervention-related changes while minimizing potential bias from baseline differences.

These clarifications have been added to the revised Methods section to improve methodological rigor.

Revision in Manuscript

Data Analysis , Page 9, Lines 199–205

Comment 4. Authors should justify the use of a 40 minute examination task for stress induction without reporting manipulation checks or post induction baseline measures.

Response

Thank you for this important comment. Thank you for this valuable comment. A post-induction baseline assessment was conducted immediately after the 40-minute examination task and before the intervention. However, this procedure was not clearly described in the original manuscript. We have now clarified this point in the revised Methods section. The examination task was selected based on prior studies showing that cognitively demanding tasks under time pressure can effectively induce acute stress responses in student populations [37]. In addition, we acknowledge that the present study lacked formal manipulation checks for the stress induction procedure. We have now added a corresponding statement in the Limitations section of the manuscript to address this issue. We agree that this aspect can be further improved in future research.

Revision in Manuscript

Methods Page 7, Lines 141–147

Limitations Page 16, Lines 358–360

Comment 5. How does the study ensure that the "standard verbal suggestions" represent actual clinical practice rather than an artificially constructed comparator?

Response

Thank you for this insightful comment.

The “standard verbal suggestions” used in this study were not artificially constructed but were adapted from commonly used scripts in clinical music therapy and relaxation training, such as breathing guidance and imagery-based facilitation. Specifically, the content of the standard verbal suggestions was derived from Introduction to Music Therapy by Professor Tian Gao, a well-established music therapy scholar who has played a pioneering role in the development of music therapy education and clinical practice in China.

According to the reviewer’s suggestion, we have clarified this point in the revised Methods section to further improve the transparency of the study design.

Revision in Manuscript

Intervention Group 2: Standard music therapy group Page 6, Lines 116–121

Comment 6. Please provide inter rater reliability data for the therapist's delivery of verbal suggestions to ensure consistency across participants within groups.

Response

Thank you for this valuable comment.

In the present study, all intervention sessions were conducted by the same trained music therapist. Therefore, inter-rater reliability was not applicable, as there were no multiple raters or therapists involved in delivering the intervention. In addition, procedural interactions were structured and systematically implemented.

We have clarified this point in the revised Methods section (Procedure).

Revision in Manuscript

Methods section, Page 7, Lines 138–139

Comment 7. Authors should report a priori power analysis and explain whether the sample size of 113 participants was sufficient to detect expected effect sizes.

Response

Thank you for this important comment.

We acknowledge that a formal a priori power analysis was not conducted before data collection, which is a limitation of the present study. To address the reviewer’s concern, we conducted a sensitivity power analysis using G*Power for a one-way ANOVA with three groups, assuming an alpha level of 0.05 and a desired power of 0.80. The analysis indicated that a total sample size of 113 participants would be detect an effect size of approximately f = 0.30.

We have added this clarification to the revised manuscript and acknowledged the limitation related to statistical power in the Discussion section.

Revision in Manuscript

Data Analysis and Limitations

Page 10 , Lines 208–210 . Page 16 , Lines 353–356.

Comment 8. How does the study address potential demand characteristics, given that participants in the Eastern imagery group might guess the cultural tailoring hypothesis?

Response

Thank you for this insightful comment.

We acknowledge that demand characteristics may have influenced participants’ responses, particularly in the culturally tailored condition. To mitigate this risk, participants were not informed of the study hypotheses, all conditions were presented as equally valid interventions, and procedures were standardized across groups.

Thank you for your comment. We have added a detailed description of this point in the Procedure section of the revised manuscript.

Revision in Manuscript

Procedure, Page 7, Lines 133–135.

Comment 9. Please clarify why the PANAS positive affect showed no changes whatsoever, and whether ceiling or floor effects might explain this null finding.

Response

Thank you for this important comment.

The absence of significant changes in positive affect (PA) was examined in relation to potential ceiling or floor effects. Baseline PA scores across the three groups were moderate (ranging from 2.51 to 2.89 on a 5-point scale), indicating that neither ceiling nor floor effects were present. Therefore, the null findings are unlikely to be explained by restricted variance at baseline.

Instead, this pattern is consistent with previous research. Prior studies have shown that music-based interventions are generally more effective in reducing negative affect than in enhancing positive affect (Blasco-Magraner et al., 2023). In addition, music listening has been demonstrated to effectively regulate negative emotional states following stress induction (Groarke and Hogan, 2019).

We have added further explanation and discussion of this issue in the revised Discussion section.

Revision in Manuscript

Discussion, Page 15, Lines 321–325.

Comment 10. Authors should explain how the eight musical pieces from Kikujiro were selected and whether their joyful character might confound comparisons with the other groups.

Response

We sincerely thank the reviewer for this thoughtful and important comment. We have revised the manuscript to clarify the rationale for selecting the eight tracks from Kikujiro.

The music was selected based on several criteria: (1) instrumental compositions without lyrics, to avoid verbal interference; (2) moderate tempo and emotionally accessible melodic structure; (3) a progressive emotional flow from mild activation to relaxation. The selected tracks were arranged to create a coherent transition from light positive arousal toward calmness and emotional stabilization.

Regarding the cheerful character of some tracks, we agree that musical valence may influence participants’ responses. However, this condition was intentionally designed as a passive music-listening relaxation control that reflected a positive but non-directive emotional environment, without verbal guidance or therapist-led intervention.

We have also acknowledged in the revised Discussion

Revision in Manuscript

Methods Page 6, Lines 122–127.

Limitations Page 16, Lines 356–358.

Comment 11. How does the study account for participants' prior familiarity with or emotional associations to the film soundtracks used as stimuli?

Response

Thank you for this insightful comment.

We acknowledge that participants’ prior familiarity with the musical materials may have influenced their emotional responses to some extent. As the primary focus of this study was on the role of verbal guidance in music-based interventions. Random assignment and standardized intervention procedures were implemented to help control for this potential confounding factor. We have also acknowledged in the revised Limitations that familiarity across conditions may have introduced potential confounding effects.

Revision in Manuscript

Methods Procedure Page 7, Lines 133–139. Limitations Page 16, Lines 356–358.

Comment 12. Please provide the full ChatGPT prompt in the methods section, including any temperature settings or other parameters that might affect output reproducibility.

Response

Thank you for this important comment.

To enhance transparency and reproducibility, we have now provided the full prompt structure used for generating the verbal suggestions in the Methods section. The prompts were originally written in Chinese, as the study was conducted in a Chinese-language context. For clarity and accessibility, both the original Chinese prompts and their English translations have been included in the revised manuscript.

Revision in Manuscript

Methods, Materials

Page 5, Lines 92–98.

Comment 13. Authors should justify the exclusion of outliers from STAI and PANAS data while retaining all heart rate data, and report sensitivity analyses with outliers included.

Response

Thank you for this important suggestion.

In the original manuscript, outliers were identified and excluded using the standard ±1.5 × interquartile range (IQR) criterion to enhance the robustness of the data. This approach was applied to the self-report measures (STAI and PANAS), as such measures are more susceptible to response biases, particularly when completed within a short time frame.

In contrast, no extreme outliers exceeding the IQR thresholds were detected in the heart rate (HR) data. Moreover, HR values in young adults typically fall within a relatively stable physiological range (approximately 40–120 bpm). Therefore, all HR data were retained for analysis.

Following the reviewer’s recommendation, we conducted additional sensitivity analyses by re-running the primary statistical tests with all previously excluded outliers included. The results indicated that the main findings remained consistent. Specifically, the within-group reduction in state anxiety in the Eastern imagery group remained significant (original p = .030; sensitivity analysis p = .046). The significant reductions in negative affect in both the Eastern imagery and standard groups were also preserved. Furthermore, all between-group comparisons using ANOVA and Kruskal–Wallis tests remained non-significant.

The results of these sensitivity analyses have been added to the revised manuscript in the Results section (under a new subsection, “Sensitivity Analyses”), and the corresponding procedures have been clarified in the Data Analysis section of the Methods. These revisions improve the transparency and robustness of the study.

Revision in Manuscript

Results and Data Analysis Page 13, Lines 283–289. Page 10, Lines 214–219.

Comment 14. How does the study ensure that the Eastern imagery verbal suggestions actually reflect culturally adapted content rather than generic poetic language?

Response

Thank you for this insightful comment, which highlights a critical aspect of cultural adaptation in the present study.

To ensure that the verbal suggestions in the Eastern imagery group genuinely reflected culturally tailored content rather than generic poetic language, several steps were implemented. First, all prompts input into ChatGPT 5.2 were written in Chinese and explicitly instructed the model to incorporate “Eastern imagery and culturally symbolic elements.” The second prompt further required the model to generate suggestive language “based on the characteristics of the Chinese film Hero.”

In addition, Table 1 has been added to the revised Methods section to provide a comparative illustration between generic relaxation scripts and the culturally adapted versions used in this study.

The generated scripts were independently reviewed and iteratively refined by the research team. The team included one author with expertise in Chinese traditional literature and a certified music therapist. Through multiple rounds of revision, culturally specific imagery (e.g., qin music, eastern wind, mountains and rivers, breath) was strengthened, while generalized relaxation expressions were minimized.

Revision in Manuscript

We have expanded the description of this procedure in the Methods section (Intervention Group 1) to improve the transparency of the study. Page 5, Lines 107–114.

Comment 15. Authors should discuss why the future scope should contain explanability and newer transformer models, please see and add [2] Tri-STANet: An advanced framework integrating dual axis transformer and morphological encoding for enhanced AS detection; [3] XAI-RACapsNet: Relevance aware capsule network-based breast cancer detection using mammography images via explainability O-net ROI segmentation

Response

Thank you for this valuable suggestion. We agree that both explainability and the use of newer transformer-based models represent important directions for future research.

We have reviewed and cited the references you recommended [55,56], and we sincerely appreciate your suggestions, which have greatly enriched our understanding of this topic. Specifically, the primary aim of the present study was to provide an initial empirical test of the feasibility of using large language models to generate culturally adapted verbal suggestions in music-based interventions. Therefore, future research should incorporate explainability analyses to clarify which linguistic features, cultural symbols, and modes of expression contribute most to therapeutic effectiveness and cultural resonance.

Revision in Manuscript

Discussion, future research Page 16, Lines 362–363.

Comment 16. Please explain why trait anxiety moderation analyses were underpowered, with only seven participants in the low trait anxiety subgroup of the Eastern imagery condition.

Response

Thank you for the reviewer’s careful evaluation. We agree that the analysis of the moderating effect of trait anxiety within the Eastern imagery group may have been underpowered due to sample imbalance, which in turn limits the statistical power of subgroup analyses.

In the revised manuscript, we have explicitly acknowledged this limitation and emphasized that the non-significant findings should be interpreted with caution. Furthermore, we suggest that future research should increase the sample size and strive to balance the proportion of participants with high and low trait anxiety in order to ensure sufficient statistical power.

Revision in Manuscript

Results Page 14, Lines 297–300.

Limitations Page 16, Lines 353–361.

Comment 17. Authors should clarify whether heart rate measurements were taken immediately post intervention or after a delay, and whether room conditions were standardized.

Response

Thank you for this important comment.

Heart rate measurements were obtained immediately after the completion of the intervention, without delay, in order to capture the immediate physiological response to the music-based intervention. In addition, all heart rate measurements were conducted under standardized environmental conditions, including controlled room temperature, lighting, and noise levels, to minimize potential external influences on physiological responses.

Revision in Manuscript

These details have now been clarified in the revised manuscript (Methods section).

Page 8, Lines 181–184.

Comment 18. How does the study address the multiple comparison problem given the numerous t tests and ANOVAs performed without correction for family wise error rate?

Response

Thank you for raising the issue of multiple comparisons. In response, we have conducted post-hoc LSD tests on the significant ANOVA results. The key findings remain as follows:

The Eastern imagery group showed significantly lower Negative Affect scores post-intervention compared to both the standard and music-only groups. The standard music therapy group exhibited a significantly greater reduction in heart rate compared to the relaxation music group (p = .006). However, the difference between the standard and Eastern imagery groups was not significant (p = .09), nor was the difference between the Eastern imagery and relaxation music groups (p = .48)

Revision in Manuscript

These post-hoc results have been added to the revised Results section Page 13, Lines 275–279.

Comment 19. Please provide the exact duration of each intervention and confirm that exposure time was precisely equivalent across all three experimental conditions.

Response

Thank you for this important question regarding intervention duration.

Upon re-checking the actual audio files used in the study, we confirm the precise durations as follows:

• Eastern imagery group (Group 1): 26 minutes 19 seconds

• Standard verbal suggestion group (Group 2): 29 minutes 56 seconds

• Music-only group (Group 3): 25 minutes 35 seconds

We acknowledge that the intervention lengths were not perfectly equivalent across the three conditions This variation occurred because we used the original commercial recordings without artificial editing to preserve the ecological validity and natural flow of the music. In the revised manuscript, we have now clearly reported the exact durations for each condition and discussed this as a minor limitation. Although the durations are not identical, all interventions fell within a narrow 25–30minute range, which is typical in music therapy research.

Revision in Manuscript

Methods

Page 5, Lines 89. Page 6, Lines 118. Page 6, Lines 128.

Response to Reviewer #2

Thank you very much for your insightful and constructive comments. These suggestions have significantly improved the rigor, clarity, and methodological transparency of the manuscript. We have carefully revised the manuscript accordingly.

Comment 1.Introduction should be shorted to maximun 1 page and a half

Response

Thank you for this valuable suggestion. We have substantially revised and condensed the Introduction section to improve clarity and conciseness. Specifically:

• Redundant descriptions of general psychological interventions (e.g., CBT, pharmacological treatments) have been reduced.

• Overlapping literature statements have been merged.

• Greater focus has been placed on the core themes of this study, including music therapy mechanisms, cultural adaptation of verbal suggestions, and the role of large language models (LLMs).

As a result, the Introduction has been reduced to approximately 1.5 pages.

Revision in manuscript:

The Introduction section (Pages 2 Lines 24–69.) has been shortened and streamlined accordingly.

Comment 2 methods: it should be added who adjudicated the quality of life

Response

Thank you for this valuable comment. Quality of life was not included in the present study. We acknowledge its importance in comprehensively evaluating intervention outcomes and will consider incorporating it in future research.

Comment 3 methods: it is not clear if authors used block sizes for the rct

Response

Thank you for this important comment. We acknowledge that block randomization may improve group balance, particularly in smaller samples. In the present study, participants were assigned using a computer-generated simple randomization procedure, and no block randomization or fixed block sizes were applied. We will consider adopting this approach in future research. Thank you again for this valuable suggestion.

Revision in Manuscript

The Procedure section (Page 7 Lines 133–139.) has been revised.

Comment 4 methods: sample size calculation should be improved

Response

We appreciate this important suggestion. We acknowledge that a formal a priori sample size calculation was not conducted prior to recruitment. Instead, we performed a sensitivity power analysis using G*Power based on the final sample size. The analysis indicated that: With N = 113, α = 0.05, and power = 0.80. The study was adequately powered to detect a medium effect size (f = 0.30).

We have clarified this in the Methods section and acknowledged it as a limitation in the Discussion section.

Revision in Manuscript

Methods (Page 9 Lines 208–210.)

Discussion (Page 16 Lines 354–356.)

Comment 5 methods/results: do authors think that embedding multivariate analysis may increase the power of the analysis?

Response

Thank you for this insightful and constructive suggestion. We agree that multivariate analytical approaches offer several advantages, including the ability to simultaneously analyze multiple dependent variables, account for correlations among variables, and improve statistical power. However, in the present study, some variables (i.e., anxiety and PANA) met the assumption of normality, whereas heart rate data did not. Therefore, a multivariate model may not applied. Thank you again for this valuable suggestion.

Response to Reviewer #3

Thank you very much for your insightful and constructive comments. These suggestions have significantly improved the rigor, clarity, and methodological transparency of the manuscript. We have carefully revised the manuscript accordingly. Below, we provide a point-by-point response.

Comment 1

The sample size appears underpowered to detect between-group differences, especially given the small effect sizes reported and the use of three groups without a clear a priori power calculation.

Response

Thank you for this important comment. We acknowledge that an a priori power analysis was not conducted prior to the study, which may have limited the statistical power to detect between-group differences. To address this limitation, a sensitivity analysis has been added to the Methods section, indicating that the current sample size (N = 113) is sufficient to detect a medium effect size (f = 0.30).

In addition, the overall effect sizes reported in the study were relatively small, suggesting that between-group differences may be limited. This limitation has been further emphasized in the Discussion section, and future research is recommended to employ larger sample sizes to improve statistical power.

Revision in Manuscript

Power analysis has been add in the Methods section Page 10 Lines 208–210

A statement regarding sample size and effect size limitations has been added to the Discussion section Page 16 Lines 354–356

Comment (2)

It’s unclear why different film soundtracks were used across the three conditions.

Response

Thank you for this important question. We apologize for not clearly explaining the rationale for using different film soundtracks across the three conditions in the original manuscript.

The selection of different music was intentional and directly aligned with the theoretical purpose of each experimental arm:

The Eastern imagery group (Group 1) used the soundtrack from the Chinese film Hero (composed by Tan Dun) because its strong Eastern aesthetic elements (e.g., guqin, traditional orchestration, and philosophical themes) complemented the LLM-generated culturally adapted verbal suggestions.

The standard verbal suggestion group (Group 2) used therapeutic relaxation music recommended in the textbook Introduction to Music Therapy by Tian Gao. This represents the conventional, culturally neutral approach commonly used in clinical receptive music therapy, serving as an active control for general verbal guidance.

The music-only group (Group 3) used the soundtrack from the Japanese film Kikujiro (composed by Joe Hisaishi), chosen for its gentle, joyful, and childlike qualities that facilitate passive emotional regulation without any verbal suggestions. This group served as a passive control.

We acknowledge that using different musical materials may introduce some confounding (e.g., differences in emotional tone or familiarity), and we have added this as a limitation in the Discussion section.

Revision in Manuscript

Methods section Page 4 Lines 85–131

Discussion section Page 16 Lines 356–359

Comment (3) The stress induction task lasted 40 minutes but is described only vaguely; without details on cognitive load or validation of its effectiveness, its role as a standardized stressor remains questionable.

Response

Thank you for this valuable comment. We have clarified and refined the description of the stress induction task in the revised manuscript.

Specifically, after obtaining written informed consent, participants completed a 40-minute time-constrained cognitive task, in which they were required to organize course-related content and construct a structured mind map. This task was designed to simulate a cognitively demanding academic activity. Prior research has demonstrated that cognitive load, defined as the mental effort involved in processing and organizing information, when combined with time constraints, can effectively induce stress responses in experimental settings [37].

The manuscript has been revised to provide a clearer description of the task, thank you.

Revision in Manuscript

Methods, Procedure Page 7 Lines 133–152

Comment (4) Heart rate was measured using a smartphone app, yet no information is provided about calibration, participant positioning, or consistency of measurement technique across individuals.

Response

Thank you for this important comment. We have clarified the heart rate measurement procedure in the revised manuscript.

In the present study, heart rate was measured using a smartphone application based on photoplethysmography (PPG). Previous studies have demonstrated that smartphone-based PPG measurements show good agreement with validated methods such as electrocardiography (ECG) under resting conditions.

To enhance measurement consistency, participants were instructed to remain seated in a relaxed position, minimize movement, and maintain consistent finger placement during measurement. Measurements were conducted under controlled environmental conditions (e.g., stable lighting and minimal noise) immediately after the intervention.

Revision in Manuscript

Heart Rate, Page 8 Lines 177–188

Comment (5) The Eastern imagery script uses poetic metaphors, but there’s no evidence that participants actually perceived these as culturally resonant or more acceptable than standard suggestions.

Response

Thank you for this insightful comment. We agree that it is important to provide evidence that participants actually perceived the Eastern imagery verbal suggestions as culturally resonant.

To address this concern, we have added a post-hoc manipulation check in the revised manuscript. Specifically, after the intervention, participants in the Eastern imagery group rated the verbal suggestions on two 7-point Likert scales: (1) the extent to which the suggestions felt culturally relevant to Chinese culture, and (2) the extent to which the suggestions felt personally resonant and meaningful to them (1 = not at all, 7 = extremely).

The results showed that participants perceived the Eastern imagery verbal suggestions as highly culturally relevant (M = 5.03, SD = 1.18) and personally resonant (M = 5.23, SD = 1.03). Both ratings were significantly higher than the scale midpoint of 4.

Revision in Manuscript

We have incorporated these manipulation check results into the Methods and Results sections. Page 9 Lines 189–193, Page 12 Lines 262–1266

Comment (6) Trait anxiety was used to stratify a subgroup analysis, but the low-anxiety group had only seven participants, making any comparison statistically unreliable and possibly misleading.

Response

Thank you for this insightful comment. The authors agree that the small sample size of the low trait anxiety group (n = 7) may limit the stability of the statistical analysis.

In addition, we would like to clarify a potential procedural factor that may have contributed to this issue. In the present study, participants completed a 40-minute cognitively demanding task prior to the administration of the STAI-T, which may have influenced the assessment of trait anxiety.

This issue has now been acknowledged as a limitation in the revised manuscript. Future research should administer trait anxiety measures prior to experimental tasks to ensure more accurate assessment.

Revision in Manuscript

To minimize the risk of overinterpretation, the results have been reported with caution, Page 14 Lines 297–300. This limitation has been explicitly acknowledged in the revised Discussion section. Page 16 Lines 360–362

Comment (7) Although ChatGPT 5.2 is named, there’s no description of prompt engineering, temperature settings, or how output consistency was ensured across potential multiple generations.

Response

Thank you for this important comment.

To enhance transparency and reproducibility, we have now provided the full prompt structure used for generating the verbal suggestions in the Methods section. The prompts were originally written in Chinese, as the study was conducted in a Chinese-language context. For clarity and accessibility, both the original Chinese prompts and their English translations have been included in the revised manuscript.

Revision in Manuscript

Methods Page 5 Lines 92–98

Comment (8) The claim that LLM-generated content is “culturally adaptive” rests solely on researcher interpretation rather than empirical validation from cultural experts or target-user feedback.

Response

Thank you for your valuable suggestion. We fully agree that cultural adaptation is the core mechanism underlying the effects of this study.

In the revised manuscript, we have added a post-hoc manipulation check. Specifically, after the intervention, participants in the Eastern imagery group rated the cultural relevance and acceptability of the verbal suggestions using a 7-point Likert scale (1 = not at all culturally relevant/acceptable, 7 = highly culturally relevant/acceptable).

The results showed that participants perceived the Eastern imagery verbal suggestions as highly culturally relevant (M = 5.03, SD = 1.18) and acceptable (M = 5.23, SD = 1.03), with both ratings being significantly higher than the midpoint score of 4. We have added the results of this manipulation check to the Methods and Results sections of the revised manuscript.

We believe that these additions strengthen the evidence for cultural adaptation as an important mechanism and enhance the mechanistic interpretation of this study.

Revision in Manuscript

Methods Page 9 Lines 189–193; Results Page 12 Lines 262–266

Comment (9) Passive music listening used Joe Hisaishi’s compositions, which are emotionally evocative—this may not serve as an inert control, potentially diluting observed differences between active and passive conditions.

Response

Thank you for this important comment. The authors acknowledge that the music of Joe Hisaishi is emotionally evocative, which may have influenced participants’ affective responses. This potential effect has now been addressed in the Discussion section of the revised manuscript. Future studies are recommended to use more neutral stimuli as control conditions to minimize such confounding influences.

Revision in Manuscript

Methods Page 6 Lines 122–131; Discussion Page 16 Lines 356–358

Comment (10) Outlier removal followed IQR rules, but with small group sizes (e.g., n=36), excluding even two outliers could disproportionately influence mean estimates and p-values.

Response

Thank you for this important suggestion.

In the original manuscript, outliers were identified and excluded using the standard ±1.5 × interquartile range (IQR) criterion to enhance the robustness of the data. This approach was applied to the self-report measures (STAI and PANAS), as such measures are more susceptible to response biases, particularly when completed within a short time frame.

We conducted additional sensitivity analyses by re-running the primary statistical tests with all previously excluded outliers included. The results indicated that the main findings remained consistent. Specifically, the within-group reduction in state anxiety in the Eastern imagery group remained significant (original p = .030; sensitivity analysis p = .046). The significant reductions in negative affect in both the Eastern imagery and standard groups were also preserved. Furthermore, all between-group comparisons using ANOVA and Kruskal–Wallis tests remained non-significant.

The results of these sensitivity analyses have been added to the revised manuscript in the Results section (under a new subsection, “Sensitivity Analyses”), and the corresponding procedures have been clarified in the Data Analysis section of the Methods. These revisions improve the transparency and robustness of the study.

Revision in Manuscript

Methods and the Data Analysis Page 10 Lines 214–219; Page 13 Lines 283–289

Comment (11) The correlation between heart rate and state anxiety change is modest yet it’s presented as meaningful support for physiological–subjective alignment without considering alternative explanations like regression to the mean.

Response

Thank you for this insightful comment. The authors agree that the observed correlation between changes in heart rate and state anxiety was modest and should be interpreted with caution.

In response, we have revised the manuscript to temper the interpretation of this finding and avoid overstating the alignment between physiological and subjective measures. This limitation has also been explicitly acknowledged in the Discussion section.

Revision in Manuscript

Results Page 13 Lines 268–271; Discussion Page 9 Lines 338–342

Response to Reviewer #4

Thank you for the reviewer’s positive and constructive evaluation of our work. We appreciate the recognition of the study’s innovation and methodological transparency. We have carefully revised the manuscript to address all comments and suggestions in detail.

Comment 1‎ Abstract: The section needs to be re-drafted to be self-contained means it has to clearly ‎show the hypothesis, methodology, techniques, and tools used, and the results obtained.‎

Response：

Thank you for this valuable suggestion. The abstract has been fully revised to ensure it is self-contained. It now clearly presents the research hypothesis, study design (randomized controlled trial), techniques employed (LLM-generated verbal suggestions), key measurement instruments, and the main results. These revisions improve both the completeness and readability of the abstract.

Revisions made in manuscript

Abstract Page 2, Lines 10–21，

‎Comment 2 Keywords: The Authors suggested updating the keywords by selecting more relevant terms. ‎Keywords play an important role in the appearance of the manuscript in scholars' searches, ‎which will give it more hits and more citations.‎

Response：Thank you for this suggestion. The keywords have been revised to include more specific and relevant terms (e.g., “large language model,” “music therapy,” “cultural adaptation,” “anxiety,” and “emotion regulation”) to enhance the manuscript’s visibility and indexing in academic databases.

Comment 3

The manuscript would benefit from more detailed reporting of how the AI-generated scripts were produced and standardized. What specific prompts and parameters were used, and how was consistency ensured across participants?

Response：

Thank you for this important comment.

To enhance transparency and reproducibility, we have now provided the full prompt structure used for generating the verbal suggestions in the Methods section. The prompts were originally written in Chinese, as the study was conducted in a Chinese-language context. For clarity and accessibility, both the original Chinese prompts and their English translations have been included in the revised manuscript.

Revisions made in manuscript

Page 5, Lines 92–98，

Comment 4

Could the authors clarify the procedures for randomization, allocation, and blinding? Additionally, were participants aware that some scripts were AI-generated, and if so, might this have influenced outcomes?

‎ Response： Thank you for this important comment. We have revised the Methods section to clarify the procedures for randomization, allocation, and blinding.

Participants were assigned to the three conditions using a computer-generated simple randomization procedure. Allocation was conducted prior to the intervention, and group assignments were implemented by the researcher following the randomization sequence. Regarding blinding, participants were not informed of the specific study hypotheses or the origin of the verbal suggestions. In particular, they were not told whether the scripts were generated by AI or developed using conventional methods. All intervention conditions were presented as equally valid forms of music-based relaxation. This approach was adopted to minimize expectancy effects and demand characteristics.

Revisions made in manuscript

Procedure Page 7, Lines 132–1139，

Comment 5

Introduction: The authors advised adding one more paragraph at the end of the section to ‎show the organization of the rest of the paper.‎

‎ Response：Thank you for this helpful suggestion. We have added a paragraph at the end of the Introduction to clearly outline the structure of the manuscript. This addition improves the organization and readability of the paper.

Revisions made in manuscript

Introduction Page 4, Lines 66–69，

Comment 6

The study suggests that culturally tailored, AI-generated imagery may help reduce state anxiety. However, is the observed improvement truly due to cultural resonance, or could it reflect expectancy effects, novelty of the AI component, or therapist influence during delivery?

Response：

Thank you for this insightful and important comment. We agree that the observed effects may not be solely attributable to cultural resonance and could be influenced by alternative factors such as expectancy effects, or therapist-related factors.

In this study, several steps were taken to reduce potential bias. Participants were not informed of the specific research hypotheses. They were also not told whether the intervention content was generated by artificial intelligence. In addition, all interventions were delivered by the same therapist using a standardized procedure.

In addition, to further clarify the influence of cultural resonance on our study outcomes, we supplemented manipulation checks to verify the cultural compatibility and perceived credibility of the Eastern imagery verbal suggestions. Relevant details can be found in our response to Comment 9.

The corresponding revisions have been incorporated into the main text: Methods, Page 9, Lines 189–193, Results, Page 12, Lines 262–266

Comment 7

Should AI-generated verbal suggestions be viewed as an active therapeutic mechanism in their own right, or are they simply tools that depend heavily on how clinicians implement and contextualize them?

‎ Response：

Thank you for raising this insightful and important question. It touches on the fundamental role that AI-generated verbal suggestions may play within therapeutic contexts.

In this study, we tend to conceptualize AI-generated verbal suggestions as a potential active therapeutic mechanism in their own right. In fact, emerging digital mental health applications provide support for this perspective. For example, AI-based systems such as Therabot have demonstrated the feasibility of delivering therapeutic interventions through dialogue.

At the same time, we acknowledge that, in the present study, the effectiveness of AI-generated verbal suggestions still depends to some extent on clinician guidance and contextualization. It is also worth noting that this line of research is being further extended in our ongoing work. Our research team has recently secured external funding and is currently collaborating with AI specialists to develop an intervention application that integrates AI-driven dialogue, verbal suggestion generation, and personalized music. Thank you very much.

Comment 8

Conclusion: The conclusion should be abstracted, so the authors should consider re-‎drafting it.‎

Response：

Thank you for this important comment. The conclusion section has been revised and rewritten to be more generalized and to avoid overgeneralization / excessive inference.

Revisions made in manuscript

Conclusion Page 17, Lines 365–374，

Comment 9

Including a manipulation check—such as participants’ perceived cultural relevance or credibility of the imagery—would strengthen the claim that cultural tailoring is the key mechanism.

Response：

Thank you for this valuable suggestion. We fully agree that adding a manipulation check for the cultural relevance and credibility of the Eastern imagery verbal suggestions would significantly strengthen the claim that cultural tailoring is the key underlying mechanism.

In the original study, we did not include such a manipulation check, which represents a limitation of this research. In the revised manuscript, we have added a post-hoc manipulation check. Specifically, after the intervention, participants in the Eastern imagery group rated the verbal suggestions on a 7-point Likert scale for cultural relevance and acceptability (1 = not at all culturally relevant/acceptable, 7 = highly culturally relevant/acceptable).

The results showed that participants perceived the Eastern imagery verbal suggestions as highly culturally relevant (M = 5.03, SD = 1.18) and acceptable (M = 5.23, SD = 1.03), with both ratings significantly higher than the scale midpoint of 4. We have added these manipulation check results to the Methods and Results sections of the revised manuscript.

We believe this addition strengthens the evidence that cultural adaptation serves as an important mechanism and enhances the mechanistic interpretation of the study.

Revisions made in manuscript

Methods, Page 9, Lines 189–193Results, Page 12, Lines 262–266

Comment 10

Future iterations of the study might consider an active control condition matched for therapist interaction, as well as a brief follow-up assessment to determine whether the observed effects are sustained beyond the immediate session.

Response：

Thank you for this constructive suggestion.

We appreciate your point regarding the need for an active control condition matched for therapist interaction. In the present study, the standard verbal suggestion group (Group 2) was designed as an active control. It included structured verbal guidance delivered by the same licensed music therapist, following conventional music therapy techniques (e.g., progressive relaxation and body-focused attention). In future iterations of the research, we plan to conduct brief follow-up assessments to examine whether the observed effects persist after the immediate session.

Thank you again for this valuable feedback, which has strengthened the interpretation and future directions of our work.

Revisions made in manuscript

Page 6, Lines 115–121

‎ Comment 11

Authors need to confirm that all acronyms are defined before being used for the first time.‎

Response：Thank you for this important reminder regarding the use of acronyms. In the revised manuscript, we have carefully checked the entire text and ensured that every acronym is now clearly defined in full upon its first appearance.

Comment 12

The three groups differ not only in content but also potentially in therapist engagement and structure. Does the passive listening condition provide an adequate comparison for evaluating the added value of guided verbal suggestions?

Response：

Thank you for this insightful and critical comment. We fully agree that you have pointed out an important methodological limitation of our study design.

In the original setup, we included three groups (LLM-generated Eastern imagery music therapy, standard music therapy, and passive music-only listening) to test the additive value of verbal guidance in music therapy, and passive music listening has been widely used as a common and valid control condition for music-based interventions in previous literature. However, we acknowledge that differences in therapist involvement and procedural structure across the three conditions may serve as confounding factors, which compromise the ability to rigorously evaluate the unique contribution of verbal guidance.

We have added a thorough discussion of this limitation in the Limitations section of the revised manuscript. Notably, our main results still showed that the two groups receiving verbal guidance (Eastern imagery group and standard music therapy group) exhibited significantly greater reductions in negative affect than the passive listening group, which provides preliminary evidence supporting the added value of verbal guidance.

For future research, we will adopt a more tightly controlled design: using identical musical stimuli, standardized procedures, and consistent therapist involvement across all groups, with only the verbal suggestion component as the independent variable, to accurately isolate the causal effect of verbal guidance.

Revision

limitation section

Page 16, Lines 356–358

Comment 13

Given that most between-group differences were not statistically significant, how should the within-group improvements be interpreted? Do they provide sufficient evidence of efficacy, or do they risk overstating the intervention’s impact?

Response：Thank you for this important and thoughtful question.

Thank you for this critical and insightful comment. We fully agree that this is a key methodological and interpretive issue that requires clear clarification.

First, the absence of statistically significant between-group differences in most outcomes can be explained by three factors: (1) All three conditions included music-based relaxation, which is an effective strategy for relieving stress and negative emotions; thus, all groups showed improvements, leading to relatively small between-group differences. (2) The sample size of this study was sufficient to detect moderate effects but lacked statistical power to identify small between-group differences.

Second, the significant within-group improvements provide preliminary and supportive evidence for the intervention effect, rather than definitive proof. Significant within-group changes in subjective anxiety, negative affect, and heart rate consistently indicate that the intervention can effectively alleviate acute stress responses. These results are consistent with a large body of music therapy literature showing that within-group pre–post improvements are commonly used to support the feasibility and effectiveness of interventions.

Third, we fully acknowledge the risk of overinterpretation and have never claimed that the results provide conclusive evidence of intervention superiority. In the revised manuscript, we have strengthened the interpretation in the Discussion section. We clearly state that the within-group significant results demonstrate the feasibility and preliminary effectiveness of the intervention, while the non-significant between-group differences suggest that the incremental value of verbal guidance requires verification in larger-sample, more tightly controlled studies. We have avoided any overstatement of the intervention effect.

We greatly appreciate your reminder, which has helped us improve the rigor and accuracy of result interpretation.

Revision

Discussion section Page 14, Lines 310–374

‎ Comment 14

The authors suggested proofreading the manuscript after addressing all comments to ‎avoid any typos, grammatical and linguistic mistakes, and errors.‎

Response：We sincerely thank the reviewer for this helpful suggestion. The entire manuscript has been carefully proofread and professionally edited for language quality. Grammar, wording, sentence structure, and overall readability have been improved throughout the revised version.

‎ Comment 15

Authors are advised to ensure that all references' format matches and complies with the ‎journal's requirements and format.‎

Response：We sincerely thank the reviewer for this helpful reminder. All references have been carefully reviewed and revised to ensure consistency and full compliance with the journal’s formatting and citation requirements. We have also checked punctuation, capitalization, journal titles, and reference style throughout the manuscript.

Response to Reviewer #5

We sincerely thank Reviewer #5 for the thoughtful and constructive comments. The suggestions were highly valuable and helped strengthen the manuscript.

Comment: The core academic value of this paper lies in AI-enabled physiotherapy and psychological intervention methods.However, the authors should emphasize the intervention target population and the alignment between the intervention and the root causes of participants’ psychological and physical discomfort.

Response：Thank you for this professional and constructive comment. We greatly appreciate your recognition of the core academic value of this study—integrating AI-enabled, culturally adapted language into music therapy.

In response to your suggestion, we have strengthened the description of the intervention target population and emphasized the alignment between the intervention design and the root causes of participants’ psychological and physical distress in the revised manuscript.

Specifically, we clearly state that the target population of this intervention is Chinese undergraduate students who suffer from acute stress, anxiety, and emotional discomfort mainly caused by academic pressure, employment concerns, and interpersonal difficulties. Our intervention—music therapy integrated with LLM-generated culturally adapted verbal suggestions—is precisely designed to target these root causes. Culturally congruent verbal guidance aligns with Chinese students’ emotional expression norms and enhances emotional engagement, while music-based relaxation effectively relieves stress-induced physiological arousal (e.g., elevated heart rate) and psychological anxiety.

We have added and refined the above content in the Introduction, Methods, and Discussion sections to highlight the fit between the intervention, the target population, and the underlying causes of their distress. This revision better highlights the practical value and theoretical contribution of this AI-enabled psychological intervention.

We greatly appreciate your suggestion, which has significantly improved the academic focus and practical relevance of this paper.

Revision in Manuscript

Introduction Page 2 Lines 24–32

Methods Page 4 Lines 76–79

Discussion Page 12 Lines 316-320, Lines 330–337

Attachment Submitted filename: Response to Reviewers.docx

# Peer review: Decision Letter 1

**DOI:** 10.1371/journal.pone.0360478.r003

**Authors:** Thiago P. Fernandes

12 Jul 2026

Dear Dr. Li,

Thank you for submitting your manuscript to PLOS ONE. After careful consideration, we feel that it has merit but does not fully meet PLOS ONE’s publication criteria as it currently stands. Therefore, we invite you to submit a revised version of the manuscript that addresses the points raised during the review process.

==============================

Please respond to all comments and highlight the changes in the revised ms.

==============================

Please submit your revised manuscript by Aug 26 2026 11:59PM. If you will need more time than this to complete your revisions, please reply to this message or contact the journal office at plosone@plos.org. When you're ready to submit your revision, log on to https://www.editorialmanager.com/pone/ and select the 'Submissions Needing Revision' folder to locate your manuscript file.

- A letter that responds to each point raised by the academic editor and reviewer(s). You should upload this letter as a separate file labeled 'Response to Reviewers'.
- A marked-up copy of your manuscript that highlights changes made to the original version. You should upload this as a separate file labeled 'Revised Manuscript with Track Changes'.
- An unmarked version of your revised paper without tracked changes. You should upload this as a separate file labeled 'Manuscript'.

If you would like to make changes to your financial disclosure, please include your updated statement in your cover letter. Guidelines for resubmitting your figure files are available below the reviewer comments at the end of this letter.

If applicable, we recommend that you deposit your laboratory protocols in protocols.io to enhance the reproducibility of your results. Protocols.io assigns your protocol its own identifier (DOI) so that it can be cited independently in the future. For instructions see: https://journals.plos.org/plosone/s/submission-guidelines#loc-laboratory-protocols. Additionally, PLOS ONE offers an option for publishing peer-reviewed Lab Protocol articles, which describe protocols hosted on protocols.io. Read more information on sharing protocols at https://plos.org/protocols?utm_medium=editorial-email&utm_source=authorletters&utm_campaign=protocols.

As the corresponding author, your ORCID iD is verified in the submission system and will appear in the published article. PLOS supports the use of ORCID, and we encourage all coauthors to register for an ORCID iD and use it as well. Please encourage your coauthors to verify their ORCID iD within the submission system before final acceptance, as unverified ORCID iDs will not appear in the published article. *Only*  the individual author can complete the verification step; PLOS staff *cannot*  verify ORCID iDs on behalf of authors.

We look forward to receiving your revised manuscript.

Kind regards,

Thiago P. Fernandes, PhD

Academic Editor

PLOS One

Journal Requirements:

If the reviewer comments include a recommendation to cite specific previously published works, please review and evaluate these publications to determine whether they are relevant and should be cited. There is no requirement to cite these works unless the editor has indicated otherwise.

[Note: HTML markup is below. Please do not edit.]

Reviewers' comments:

Reviewer's Responses to Questions

**Comments to the Author**

Reviewer #1: All comments have been addressed

Reviewer #2: All comments have been addressed

Reviewer #4: All comments have been addressed

Reviewer #6: (No Response)

Reviewer #7: All comments have been addressed

**********

2. Is the manuscript technically sound, and do the data support the conclusions?

Reviewer #1: Yes

Reviewer #2: Yes

Reviewer #4: Yes

Reviewer #6: No

Reviewer #7: Yes

**********

3. Has the statistical analysis been performed appropriately and rigorously?

Reviewer #1: Yes

Reviewer #2: Yes

Reviewer #4: Yes

Reviewer #6: No

Reviewer #7: Yes

**********

4. Have the authors made all data underlying the findings in their manuscript fully available?

The PLOS Data policy

Reviewer #1: Yes

Reviewer #2: Yes

Reviewer #4: Yes

Reviewer #6: Yes

Reviewer #7: Yes

**********

5. Is the manuscript presented in an intelligible fashion and written in standard English?

Reviewer #1: Yes

Reviewer #2: Yes

Reviewer #4: No

Reviewer #6: No

Reviewer #7: Yes

**********

Reviewer #1: The authors have diligently incorporated all feedback provided into the revised version of the manuscript, ensuring that each comment has been adequately addressed and resolved to satisfaction.

Reviewer #2: All comments have been addressed and authors should be complimented for this. I think it should be accepted

Reviewer #4: The authors have diligently revised and improved the manuscript based on the feedback provided by reviewers during the previous review cycle. As a result of these revisions, the quality of the manuscript now aligns with the standards set by the journal. However, upon thorough examination of the manuscript, it becomes evident that there are certain linguistic and grammatical issues present throughout. To address these concerns, the authors are strongly advised to have the manuscript proofread by a native English speaker. This step will help rectify any lingering linguistic or grammatical errors and ensure that the manuscript reads fluently and coherently. Additionally, it is imperative for the authors to meticulously review and confirm that all references cited in the manuscript adhere to the journal's prescribed referencing style and format. Ensuring consistency and accuracy in reference formatting is crucial for maintaining the integrity and professionalism of the manuscript. By taking these measures to address language and referencing issues, the authors can further enhance the clarity, readability, and overall quality of their manuscript, thereby facilitating a smoother publication process and better dissemination of their research findings.

Reviewer #6: This paper tests whether verbal suggestions written by ChatGPT and framed around Chinese cultural imagery can make music therapy more effective at reducing anxity in university students. The idea is genuinely novel and practically relevant, and the study uses a proper randomized controlled trial design with three groups, measuring anxiety and heart rate before and after the intervention. At first glance, it reads as a competent piece of applied research. However, the paper has a fundamental problem that must be addressed before this work can move forward.

The central claim that "LLM-generated, culturally adapted verbal suggestions produced better outcomes" is simply not supported by the data. The main statistical test, the between-group comparison, shows no significant differences across any primary outcome. The authors pivot to within-group improvements as their main finding, but that is not what an RCT is designed to demonstrate. All three groups got better, and the study cannot tell us why. The title, abstract, and conclusion must be rewritten to reflect this honestly. The paper can and should be reframed as a pilot feasibility study, which is a legitimate and publishable contribution - but the current framing constitutes overclaiming and does not meet the standard that conclusions be drawn appropriately from the data presented.

Beyond the null result, the study design has a confound that runs through every conclusion the authors draw. Each group listened to completely different music - Chinese film music, a clinical therapy recording, and Japanes film music, composed by different people, in different styles, with different emotional characters, and with run times that vary by over four minutes. Trying to isolate the effect of verbal suggestion content when the music itself is this different is not possible. Any outcome difference could be due to the music, the words, familiarity with the soundtrack, the emotional tone of the piece, or any combination of these. This is acknowledged as a limitation in the manuscript, but it is actually a design flaw that prevents the key comparison from being made. The authors should explicitly state this in the discussion, rather than treating it as a minor caveat.

There are also concerns about the two references added in this revision, References 55 and 56. These papers are about aortic stenosis detection and breast cancer imaging using deep learning. They have no relevance to music therapy, cultural adaptation, or anxiety research. Are these references genuinely necessary?

Several other issues compound the concern. The Cultural Resonance Manipulation Check was added after data collection in response to reviewer comments and was only administered to one group - without comparative data from the other two groups, it cannot establish that the Eastern imagery sugestions were perceived as more culturally resonant than standard suggestions, only that participants approved of them. It must be labeled post-hoc and exploratory, and the causal language around it must be removed. The sample is 94% women, with no sex-stratified analysis and no discussion of what this means for generalizability. The correlation analysis reports n = 115 when only 113 participants were enrolled - this discrepancy must be identified and corrected. The stress induction task has no validated manipulation check confirming it actually elevated anxiety.

So, the paper addresses an interesting question, and with honest reframing and thorough revision it can make a real contribution to this emerging area. But in its current form, the evidence does not support the conclusions being drawn, and the design cannot adequately test the core hypothesis. Major structural revision is required before this can be considered for publication.

Reviewer #7: The revisions and the Authors’ Response are quite good and generally acceptable, especially since the authors have substantively addressed most of the key methodological issues. The manuscript has provided clarifications regarding music segmentation, randomization and blinding procedures, LLM prompts, cultural manipulation, heart rate measurements, sensitivity analysis for outliers, and acknowledged limitations related to statistical power, non-identical intervention durations, differences in musical stimuli, and potential confounding by therapist or demand characteristics. However, this acceptance should be viewed with caution. The article’s contribution remains stronger as a preliminary/exploratory study rather than conclusive evidence of efficacy, as many between-group results were not significant, the power analysis was conducted post hoc/as a sensitivity analysis, and some methodological controls are still not ideal. Therefore, the authors’ response is “acceptable” for proceeding with publication, provided that the claims in the abstract, discussion, and conclusions are toned down to avoid overstating the effectiveness of the LLM-assisted music therapy intervention.

**********

what does this mean?). If published, this will include your full peer review and any attached files.

If you choose “no”, your identity will remain anonymous but your review may still be made public.

**Do you want your identity to be public for this peer review?**  For information about this choice, including consent withdrawal, please see our Privacy Policy

Reviewer #1: **Yes:**  Mohd Anul Haq

Reviewer #2: **Yes:**  Fabrizio D'Ascenzo

Reviewer #4: No

Reviewer #6: No

Reviewer #7: No

**********

[NOTE: If reviewer comments were submitted as an attachment file, they will be attached to this email and accessible via the submission site. Please log into your account, locate the manuscript record, and check for the action link "View Attachments". If this link does not appear, there are no attachment files.]

To ensure your figures meet our technical requirements, please review our figure guidelines: https://journals.plos.org/plosone/s/figures

You may also use PLOS’s free figure tool, NAAS, to help you prepare publication quality figures: https://journals.plos.org/plosone/s/figures#loc-tools-for-figure-preparation.

NAAS will assess whether your figures meet our technical requirements by comparing each figure against our figure specifications.

# Author comment: Author response to Decision Letter 2

**DOI:** 10.1371/journal.pone.0360478.r004

11 Aug 2026

Response to Reviewers

Dear Editor and Reviewers,

Thank you for the opportunity to revise and resubmit our manuscript. We sincerely appreciate the time, effort, and constructive comments provided by the Editor and all reviewers. Their insightful suggestions were highly valuable and helped improve the clarity, rigor, and overall quality of the manuscript.

We carefully considered every comment and revised the manuscript accordingly. Below, we provide a detailed point-by-point response to each reviewer’s comments. We hope that the revised manuscript meets the journal’s standards and we sincerely appreciate your further consideration.

Sincerely,

The authors

Response to Reviewer #1

Comment：

The authors have diligently incorporated all feedback provided into the revised version of the manuscript, ensuring that each comment has been adequately addressed and resolved to satisfaction.

Response

Thank you very much. We sincerely appreciate your time, constructive comments, and valuable support throughout the review process.

Response to Reviewer #2

Comment：

All comments have been addressed and authors should be complimented for this. I think it should be accepted.

Response

Thank you very much for your positive evaluation of our manuscript and for recommending its publication. We sincerely appreciate your time, constructive comments, and valuable support throughout the review process.

Response to Reviewer #4

Comment：

The authors have diligently revised and improved the manuscript based on the feedback provided by reviewers during the previous review cycle. As a result of these revisions, the quality of the manuscript now aligns with the standards set by the journal. However, upon thorough examination of the manuscript, it becomes evident that there are certain linguistic and grammatical issues present throughout. To address these concerns, the authors are strongly advised to have the manuscript proofread by a native English speaker. This step will help rectify any lingering linguistic or grammatical errors and ensure that the manuscript reads fluently and coherently. Additionally, it is imperative for the authors to meticulously review and confirm that all references cited in the manuscript adhere to the journal's prescribed referencing style and format. Ensuring consistency and accuracy in reference formatting is crucial for maintaining the integrity and professionalism of the manuscript. By taking these measures to address language and referencing issues, the authors can further enhance the clarity, readability, and overall quality of their manuscript, thereby facilitating a smoother publication process and better dissemination of their research findings.

Response

Thank you for your valuable suggestion. To further improve the linguistic quality and readability of the manuscript, we invited two individuals with relevant expertise to carefully proofread and revise the manuscript. One is a native English speaker, and the other is a PhD in English linguistics. The manuscript has been thoroughly revised based on their feedback. In addition, we carefully rechecked all references to ensure that they comply with the journal's requirements.

Response to Reviewer #6

This paper tests whether verbal suggestions written by ChatGPT and framed around Chinese cultural imagery can make music therapy more effective at reducing anxity in university students. The idea is genuinely novel and practically relevant, and the study uses a proper randomized controlled trial design with three groups, measuring anxiety and heart rate before and after the intervention. At first glance, it reads as a competent piece of applied research. However, the paper has a fundamental problem that must be addressed before this work can move forward.

Comment 1. The central claim that "LLM-generated, culturally adapted verbal suggestions produced better outcomes" is simply not supported by the data. The main statistical test, the between-group comparison, shows no significant differences across any primary outcome. The authors pivot to within-group improvements as their main finding, but that is not what an RCT is designed to demonstrate. All three groups got better, and the study cannot tell us why. The title, abstract, and conclusion must be rewritten to reflect this honestly. The paper can and should be reframed as a pilot feasibility study, which is a legitimate and publishable contribution - but the current framing constitutes overclaiming and does not meet the standard that conclusions be drawn appropriately from the data presented.

Response

Thank you for this important and constructive comment. We agree with your assessment that, given the absence of statistically significant between-group differences and the methodological limitations of the current design, the study is more appropriately framed as a pilot feasibility study rather than as a definitive evaluation of intervention effectiveness.

In response to your observation, we have revised the title to more accurately reflect the exploratory nature and methodological scope of the study. The manuscript is now explicitly framed as a pilot randomized feasibility study rather than a confirmatory effectiveness trial. Corresponding revisions have also been made throughout the Abstract, research aims, Discussion, and Conclusion to avoid causal, confirmatory, or efficacy-related claims that are not fully supported by the data.

You also noted that “All three groups got better, and the study cannot tell us why.” We agree with this concern and have addressed it explicitly in the revised Discussion. One possible explanation is that the musical materials themselves, including their melodic, stylistic, and emotional characteristics, may have contributed to the observed reductions in anxiety. We sincerely appreciate your suggestion, which has helped us substantially improve the framing and interpretation of the study.

Revisions made in manuscript

The Title, Abstract, Methods, Discussion, and Conclusion sections have been revised.

Comment 2. Beyond the null result, the study design has a confound that runs through every conclusion the authors draw. Each group listened to completely different music - Chinese film music, a clinical therapy recording, and Japanes film music, composed by different people, in different styles, with different emotional characters, and with run times that vary by over four minutes. Trying to isolate the effect of verbal suggestion content when the music itself is this different is not possible. Any outcome difference could be due to the music, the words, familiarity with the soundtrack, the emotional tone of the piece, or any combination of these. This is acknowledged as a limitation in the manuscript, but it is actually a design flaw that prevents the key comparison from being made. The authors should explicitly state this in the discussion, rather than treating it as a minor caveat.

Response

Thank you very much for identifying this important design issue. We agree that the use of different musical materials across the three experimental conditions represents an important methodological limitation of the study design.

In response to this concern, we first expanded the Discussion to explain one possible reason why no significant between-group differences in anxiety reduction were observed. Second, in the Limitations, we explicitly acknowledge that the use of different musical materials across conditions constitutes an important confounding factor. We also suggest that future studies should use identical musical materials across all conditions to allow a more accurate evaluation of the effects of different verbal suggestions.

At the same time, we would like to further clarify the rationale behind our original design. Music itself carries cultural characteristics, and music from different cultural backgrounds may convey different aesthetic imagery and cultural meanings. Therefore, we intentionally paired culturally similar musical materials with the corresponding verbal suggestions to enhance the cultural consistency of the intervention content. Nevertheless, we fully recognise that, although this design helped preserve the cultural integrity of each intervention condition, it also limited our ability to isolate the independent effect of the verbal suggestions.

Although no significant between-group differences were observed, we sincerely appreciate your recognition of the value of this study as a pilot and exploratory investigation. We are currently continuing this line of research and have already adopted a revised design in a follow-up study, in which all groups receive identical musical materials while only the verbal suggestions differ. This will allow us to evaluate the unique contribution of culturally adapted verbal suggestions more rigorously. Once again, we sincerely thank you for your valuable comments and suggestions.

Revisions made in manuscript

Discussion: p. 15, lines 315–325;

Study Limitations: p. 17, lines 366–370.

Comment 3. There are also concerns about the two references added in this revision, References 55 and 56. These papers are about aortic stenosis detection and breast cancer imaging using deep learning. They have no relevance to music therapy, cultural adaptation, or anxiety research. Are these references genuinely necessary?

Response

Thank you for your suggestion. We have removed the two references (formerly References 55 and 56) identified in your comment, together with the corresponding citations in the manuscript. Please note that the current References 55 and 56 are newly added sources that support our discussion of the potential confounding effects arising from differences in the musical materials used across the intervention groups.

Comment 4. Several other issues compound the concern. The Cultural Resonance Manipulation Check was added after data collection in response to reviewer comments and was only administered to one group - without comparative data from the other two groups, it cannot establish that the Eastern imagery sugestions were perceived as more culturally resonant than standard suggestions, only that participants approved of them. It must be labeled post-hoc and exploratory, and the causal language around it must be removed.

Response

Thank you for raising this important point. We agree that the assessment of perceived cultural resonance was conducted after the completion of the primary data collection in response to the reviewers' comments.

Following your suggestion, we have removed the term "manipulation check" and replaced it with "Post-hoc Exploratory Assessment of Perceived Cultural Resonance." We have removed all causal language and revised the description to indicate that the findings provide preliminary descriptive evidence rather than supporting causal inferences.

Once again, we sincerely thank you for this valuable suggestion.

Revisions made in manuscript

Methods: p. 9, lines 195–201;

Results: p. 12, lines 268–273.

Comment 5. The sample is 94% women, with no sex-stratified analysis and no discussion of what this means for generalizability.

Response

Thank you for pointing out this important limitation. We acknowledge that the sample was predominantly female, with 94 female and 19 male participants among the total sample of 113 participants. Accordingly, we have added this limitation to the manuscript and recommended that future studies recruit more gender-balanced samples. You also suggested conducting gender-stratified analyses. However, because the number of male participants was relatively small (n = 19), further stratification by experimental condition would have resulted in very small subgroup sizes and limited statistical power. Therefore, gender-stratified analyses were not performed in the present study.

Once again, we sincerely thank you for your valuable comments and suggestions.

Revisions made in manuscript

Limitations: p. 17, lines 363–366.

Comment 6. The correlation analysis reports n = 115 when only 113 participants were enrolled - this discrepancy must be identified and corrected.

Response

Thank you for pointing out this important data consistency issue.

We carefully rechecked the original dataset and the statistical analysis outputs and confirmed that all analyses were conducted using data from 113 participants. We have corrected this error throughout the manuscript. Thank you very much for bringing it to our attention.

Revisions made in manuscript

Results (Objective Anxiety Measurement [Heart Rate]): p. 13, line 278.

Comment 7. The stress induction task has no validated manipulation check confirming it actually elevated anxiety.

Response

Thank you for raising this important point.

We agree that the present study could not directly verify whether the 40-minute cognitive task successfully increased participants' anxiety levels. We have acknowledged this as a limitation in the Discussion and noted that future studies should assess participants' state anxiety both before and after the stress induction task to verify the effectiveness of the anxiety induction procedure.

Once again, we sincerely thank you for this valuable suggestion.

Revisions made in manuscript

Procedure: p. 7, lines 146–149;

Limitations: p. 17, lines 370–374.

Comment 8. So, the paper addresses an interesting question, and with honest reframing and thorough revision it can make a real contribution to this emerging area. But in its current form, the evidence does not support the conclusions being drawn, and the design cannot adequately test the core hypothesis. Major structural revision is required before this can be considered for publication.

Response

We sincerely thank you for your comprehensive and constructive evaluation of our manuscript. In response to your comments, we have undertaken substantial structural revisions. The revisions include:

1. Reframing the study as a pilot randomized feasibility study, rather than a definitive evaluation of intervention effectiveness.

2. Revising the Title (p. 1, lines 1–4), Abstract (p. 2, lines 13–25), Research Aims (p. 4, lines 63–71), Discussion (p. 15, lines 311–329; p. 17, lines 363–376), and Conclusion (p. 17, lines 378–386) to ensure that the claims are appropriately aligned with the level of evidence provided by the data.

3. Explicitly acknowledging the limitation arising from the use of different musical materials across the three intervention conditions and recommending that future studies use identical musical materials across conditions.

4. Avoiding overstatement of the effectiveness of LLM-assisted music-based interventions.

5. Removing irrelevant references.

6. Correcting the reported sample size.

7. Acknowledging the limitation of the unbalanced gender distribution.

We sincerely thank you again for your thoughtful and constructive comments, which have helped us substantially improve the quality and clarity of our manuscript.

Response to Reviewer #7

Comment：

The revisions and the Authors’ Response are quite good and generally acceptable, especially since the authors have substantively addressed most of the key methodological issues. The manuscript has provided clarifications regarding music segmentation, randomization and blinding procedures, LLM prompts, cultural manipulation, heart rate measurements, sensitivity analysis for outliers, and acknowledged limitations related to statistical power, non-identical intervention durations, differences in musical stimuli, and potential confounding by therapist or demand characteristics. However, this acceptance should be viewed with caution. The article’s contribution remains stronger as a preliminary/exploratory study rather than conclusive evidence of efficacy, as many between-group results were not significant, the power analysis was conducted post hoc/as a sensitivity analysis, and some methodological controls are still not ideal. Therefore, the authors’ response is “acceptable” for proceeding with publication, provided that the claims in the abstract, discussion, and conclusions are toned down to avoid overstating the effectiveness of the LLM-assisted music therapy intervention.

Response

Thank you for this important comment. Accordingly, we have substantially revised the manuscript by reframing the study as a pilot randomized feasibility study rather than a definitive evaluation of intervention effectiveness.

We have also made corresponding revisions to the Title (p. 1, lines 1–4), Abstract (p. 2, lines 13–25), Research Aims (p. 4, lines 63–71), Discussion (p. 15, lines 311–329; p. 17, lines 363–376), and Conclusion (p. 17, lines 378–386) to clearly position the study as a preliminary and exploratory investigation. Throughout the revised manuscript, we have avoided overstating the effectiveness of the LLM-assisted music-based intervention and have ensured that the conclusions remain consistent with the evidence provided by the data.

Once again, we sincerely thank you for this valuable suggestion, which has helped us improve the scientific rigor and overall presentation of the manuscript.

Attachment Submitted filename: Response to Reviewer .docx

# Editor report: Decision Letter 2

**DOI:** 10.1371/journal.pone.0360478.r005

**Authors:** Thiago P. Fernandes

17 Aug 2026

Dear Dr. Li,

Thank you for submitting your manuscript to PLOS ONE. After careful consideration, we feel that it has merit but does not fully meet PLOS ONE’s publication criteria as it currently stands. Therefore, we invite you to submit a revised version of the manuscript that addresses the points raised during the review process.

==============================

The authors have addressed most of the previous comments, and the revised manuscript, "Integrating Large Language Model-Generated Culturally Adapted Verbal Suggestions into Music Therapy for University Students: A Pilot Randomised Feasibility Study," is improved in structure and tone compared with the original submission. That said, several issues raised previously have only been partially resolved, and a number of statistical and conceptual inconsistencies remain unaddressed or have introduced new discrepancies. These are detailed below and should be resolved before the manuscript can be considered further.

1) The manuscript presents several statistical inconsistencies that would benefit from careful reconciliation. The Methods state that heart rate data were not normally distributed and would be analysed using non-parametric methods, yet the Results present paired t-tests for heart rate. Additionally, line 280 refers to the between-group analysis as a "one-way ANOVA," whilst the Methods, the Dunn's post-hoc test, and Table 3 all indicate that a Kruskal-Wallis test was employed. More concerning is the discrepancy between the main analysis, which reports a significant between-group difference in heart rate change (p = .02), and the sensitivity analysis, which states that all comparisons, including heart rate, were non-significant. Given that no heart rate outliers were removed, these analyses should logically operate on the same dataset;

- Furthermore, the same t-value (t = 2.07) is reported with p = .03 in the main analysis and p = .046 in the sensitivity analysis, which appears mathematically inconsistent. Finally, there are mismatches between the narrative text and tables. The text reports p = .07 for negative affect at baseline whilst Table 2 shows p = .10. Similarly, for heart rate, the text reports p = .43 whilst Table 3 presents p = .48. The authors might consider verifying these values against the raw data and clarifying which figures are correct, or providing an explanation for any genuine differences between analyses;

2) This study is described as a "feasibility study," yet no feasibility outcomes have been formally defined or reported. Standard feasibility metrics such as recruitment rate, retention, intervention completion, adherence, protocol fidelity, acceptability, and adverse events are absent. Additionally, any pre-specified progression criteria that would support a transition to a definitive trial have not been provided. The post-hoc cultural evaluation, conducted in a single group, is insufficient to substantiate a claim of feasibility on its own;

- The authors might consider two options moving forward. One possibility would be to strengthen the feasibility framework by explicitly defining and reporting these outcomes in the Results section, along with clear progression criteria for a future trial. Alternatively, the study could be reframed more modestly as a "pilot randomised exploratory study" if a comprehensive feasibility assessment is not feasible given the data already collected. In either case, statements asserting that feasibility was "confirmed" or "demonstrated" would benefit from softer language that better reflects what the data actually show;

3) Although the Title and abstract have been revised, the body of the manuscript contains several claims that exceed what the data support. These include "indicating the effectiveness of this intervention," "supports the functional role of verbal guidance," "potential advantage of culturally adapted language," "broadly applicable regulatory effect," and "feasible and promising." These phrases might be reconsidered in favour of more measured language such as "suggesting a possible effect that warrants further investigation" or "an effect that may warrant testing in broader populations," which better reflect the exploratory nature of the work;

- More importantly, the change in negative affect did not differ significantly between groups (p = .52), yet the discussion implies a between-group advantage based on a post-test difference alone. This is problematic given that the groups differed at baseline, meaning any post-test difference may reflect pre-existing disparities rather than a treatment effect. The authors might explore whether reanalysing this outcome using an ANCOVA or a group by time interaction model that accounts for baseline differences would alter the interpretation, or alternatively, consider whether the interpretive claim of a between-group advantage should be reconsidered in light of these baseline differences.

Overall, the manuscript addresses a timely and interesting question, and the effort to integrate LLM-generated culturally adapted verbal suggestions into music therapy is commendable. Addressing the statistical and conceptual points raised above would meaningfully strengthen the rigour and credibility of the study, and the authors are encouraged to revise the manuscript with these considerations in mind.

==============================

Please submit your revised manuscript by Oct 01 2026 11:59PM. If you will need more time than this to complete your revisions, please reply to this message or contact the journal office at plosone@plos.org. When you're ready to submit your revision, log on to https://www.editorialmanager.com/pone/ and select the 'Submissions Needing Revision' folder to locate your manuscript file.

- A letter that responds to each point raised by the academic editor and reviewer(s). You should upload this letter as a separate file labeled 'Response to Reviewers'.
- A marked-up copy of your manuscript that highlights changes made to the original version. You should upload this as a separate file labeled 'Revised Manuscript with Track Changes'.
- An unmarked version of your revised paper without tracked changes. You should upload this as a separate file labeled 'Manuscript'.

If you would like to make changes to your financial disclosure, please include your updated statement in your cover letter. Guidelines for resubmitting your figure files are available below the reviewer comments at the end of this letter.

If applicable, we recommend that you deposit your laboratory protocols in protocols.io to enhance the reproducibility of your results. Protocols.io assigns your protocol its own identifier (DOI) so that it can be cited independently in the future. For instructions see: https://journals.plos.org/plosone/s/submission-guidelines#loc-laboratory-protocols. Additionally, PLOS ONE offers an option for publishing peer-reviewed Lab Protocol articles, which describe protocols hosted on protocols.io. Read more information on sharing protocols at https://plos.org/protocols?utm_medium=editorial-email&utm_source=authorletters&utm_campaign=protocols.

As the corresponding author, your ORCID iD is verified in the submission system and will appear in the published article. PLOS supports the use of ORCID, and we encourage all coauthors to register for an ORCID iD and use it as well. Please encourage your coauthors to verify their ORCID iD within the submission system before final acceptance, as unverified ORCID iDs will not appear in the published article. *Only*  the individual author can complete the verification step; PLOS staff *cannot*  verify ORCID iDs on behalf of authors.

We look forward to receiving your revised manuscript.

Kind regards,

Thiago P. Fernandes, PhD

Academic Editor

PLOS One

Journal Requirements:

If the reviewer comments include a recommendation to cite specific previously published works, please review and evaluate these publications to determine whether they are relevant and should be cited. There is no requirement to cite these works unless the editor has indicated otherwise.

Please review your reference list to ensure that it is complete and correct. If you have cited papers that have been retracted, please include the rationale for doing so in the manuscript text, or remove these references and replace them with relevant current references. Any changes to the reference list should be mentioned in the rebuttal letter that accompanies your revised manuscript. If you need to cite a retracted article, indicate the article’s retracted status in the References list and also include a citation and full reference for the retraction notice.

[Note: HTML markup is below. Please do not edit.]

[NOTE: If reviewer comments were submitted as an attachment file, they will be attached to this email and accessible via the submission site. Please log into your account, locate the manuscript record, and check for the action link "View Attachments". If this link does not appear, there are no attachment files.]

To ensure your figures meet our technical requirements, please review our figure guidelines: https://journals.plos.org/plosone/s/figures

You may also use PLOS’s free figure tool, NAAS, to help you prepare publication quality figures: https://journals.plos.org/plosone/s/figures#loc-tools-for-figure-preparation.

NAAS will assess whether your figures meet our technical requirements by comparing each figure against our figure specifications.

# Author comment: Author response to Decision Letter 3

**DOI:** 10.1371/journal.pone.0360478.r006

7 Sep 2026

Response to Editor and Reviewers

Dear Editor and Reviewers,

Thank you for the opportunity to revise and resubmit our manuscript. We sincerely appreciate the time, effort, and constructive comments provided by the Editor and all reviewers. Their insightful suggestions were highly valuable and helped improve the clarity, rigor, and overall quality of the manuscript.

We carefully considered every comment and revised the manuscript accordingly. Below, we provide a detailed point-by-point response to each reviewer’s comments. We hope that the revised manuscript meets the journal’s standards and we sincerely appreciate your further consideration.

Sincerely,

The authors

Comment 1.

The manuscript presents several statistical inconsistencies that would benefit from careful reconciliation. The Methods state that heart rate data were not normally distributed and would be analysed using non-parametric methods, yet the Results present paired t-tests for heart rate. Additionally, line 280 refers to the between-group analysis as a "one-way ANOVA," whilst the Methods, the Dunn's post-hoc test, and Table 3 all indicate that a Kruskal-Wallis test was employed. More concerning is the discrepancy between the main analysis, which reports a significant between-group difference in heart rate change (p = .02), and the sensitivity analysis, which states that all comparisons, including heart rate, were non-significant. Given that no heart rate outliers were removed, these analyses should logically operate on the same dataset;

- Furthermore, the same t-value (t = 2.07) is reported with p = .03 in the main analysis and p = .046 in the sensitivity analysis, which appears mathematically inconsistent. Finally, there are mismatches between the narrative text and tables. The text reports p = .07 for negative affect at baseline whilst Table 2 shows p = .10. Similarly, for heart rate, the text reports p = .43 whilst Table 3 presents p = .48. The authors might consider verifying these values against the raw data and clarifying which figures are correct, or providing an explanation for any genuine differences between analyses;

Response

We thank the reviewer for identifying these inconsistencies. In response to this comment and Comment 3 below, we revised the primary analytical approach by replacing the between-group comparisons of change scores with baseline-adjusted analyses of covariance (ANCOVAs). Accordingly, all relevant statistical results, tables, and narrative descriptions were recalculated and revised. The revised manuscript now reports adjusted means with 95% confidence intervals, omnibus F tests, partial eta squared (ηp²), and Holm-adjusted pairwise comparisons. We appreciate the reviewer’s helpful recommendation.

Regarding heart rate, although the raw scores were not normally distributed, the ANCOVA residuals were approximately normally distributed (Shapiro–Wilk test, p = .719), the residual variances did not differ significantly across groups (Levene’s test, p = .073), and the homogeneity-of-regression-slopes assumption was satisfied (group × baseline interaction, p = .412). We therefore used ANCOVA consistently as the primary analysis for heart rate.

The reviewer was also correct regarding the paired-samples test for state anxiety. The correct result was t(35) = 2.07, p = .046 (two-sided). Re-examination of the previous analyses showed that the baseline ANOVA result for negative affect should have been p = .07. For heart rate, the ANOVA result should have been p = .43, whereas the Kruskal–Wallis result should have been p = .48. However, because the revised manuscript now uses baseline-adjusted ANCOVA as the primary between-group analysis, these superseded analyses and values have been removed.

Finally, the corrected primary and sensitivity analyses are now reported consistently. No heart-rate observation met the prespecified model-outlier criterion; consequently, the primary and outlier-based sensitivity datasets were identical, and the numerical results remained unchanged.

Revisions made in manuscript

Methods—Data Analysis, p. 9, lines 206–222;

Results—p. 10, lines 224–260; Tables 2–4.

Sensitivity Analyses, p. 13, lines 271–281, and all relevant statements in the Abstract and Discussion.

Comment 2.

This study is described as a "feasibility study," yet no feasibility outcomes have been formally defined or reported. Standard feasibility metrics such as recruitment rate, retention, intervention completion, adherence, protocol fidelity, acceptability, and adverse events are absent. Additionally, any pre-specified progression criteria that would support a transition to a definitive trial have not been provided. The post-hoc cultural evaluation, conducted in a single group, is insufficient to substantiate a claim of feasibility on its own;

- The authors might consider two options moving forward. One possibility would be to strengthen the feasibility framework by explicitly defining and reporting these outcomes in the Results section, along with clear progression criteria for a future trial. Alternatively, the study could be reframed more modestly as a "pilot randomised exploratory study" if a comprehensive feasibility assessment is not feasible given the data already collected. In either case, statements asserting that feasibility was "confirmed" or "demonstrated" would benefit from softer language that better reflects what the data actually show;

Response

We agree with the reviewer. We have reframed the study as a “pilot randomized exploratory study” and revised the title accordingly: “Integrating Large Language Model-Generated Culturally Adapted Verbal Suggestions into Music Therapy for University Students: A Pilot Randomized Exploratory Study.” Statements claiming that feasibility was “confirmed” or “demonstrated” have been removed.

Revisions made in manuscript

Title; p.1

Abstract, p. 2, line 14;

Methods—p. 4, lines 79–80;

Discussion, p. 14, lines 295–296;

Conclusion, p. 17, lines 370–375.

Comment 3.

Although the Title and abstract have been revised, the body of the manuscript contains several claims that exceed what the data support. These include "indicating the effectiveness of this intervention," "supports the functional role of verbal guidance," "potential advantage of culturally adapted language," "broadly applicable regulatory effect," and "feasible and promising." These phrases might be reconsidered in favour of more measured language such as "suggesting a possible effect that warrants further investigation" or "an effect that may warrant testing in broader populations," which better reflect the exploratory nature of the work;

- More importantly, the change in negative affect did not differ significantly between groups (p = .52), yet the discussion implies a between-group advantage based on a post-test difference alone. This is problematic given that the groups differed at baseline, meaning any post-test difference may reflect pre-existing disparities rather than a treatment effect. The authors might explore whether reanalysing this outcome using an ANCOVA or a group by time interaction model that accounts for baseline differences would alter the interpretation, or alternatively, consider whether the interpretive claim of a between-group advantage should be reconsidered in light of these baseline differences.

Response

We agree with the reviewer and have moderated the relevant claims throughout the Results, Discussion, and Conclusion. Specifically, we removed or revised the following expressions: “indicating the effectiveness of this intervention,” “supports the functional role of verbal guidance,” “potential advantage of culturally adapted language,” “broadly applicable regulatory effect,” and “feasible and promising.” The revised manuscript describes the findings as possible differences between intervention conditions that require confirmation in a larger-scale trial.

Following the reviewer’s recommendation, we reanalysed all available data using ANCOVA. The post-intervention negative-affect score as the dependent variable and the baseline negative-affect score as the covariate. The baseline-adjusted group effect was statistically significant, F(2, 109) = 4.60, p = .012, ηp² = .078. Pairwise comparisons were conducted with Holm correction. This analysis replaces the previous interpretation based on unadjusted comparisons.

Revisions made in manuscript

Page 2, lines 24–27:

“The findings identify potentially meaningful response patterns but do not establish efficacy or the independent effect of culturally adapted verbal suggestions because the conditions differed in musical material and duration. Larger, controlled trials are required.”

Page 14, lines 306–308:

“The absence of a significant adjusted group effect therefore provides no evidence that the Eastern imagery condition reduced state anxiety more than the standardized therapy or music-only conditions.”

Page 15, lines 321–324:

“Nevertheless, the result cannot be attributed specifically to cultural adaptation or LLM generation. The Eastern imagery condition combined a distinctive musical repertoire with culturally framed language, whereas the comparison conditions differed on several components. The finding should therefore be regarded as a possible signal warranting confirmatory testing.”

Page 16, lines 344–345:

“This null finding should not be interpreted as evidence that the intervention is broadly applicable across trait-anxiety levels.”

Page 16, lines 346–347:

“The subgroup findings are therefore descriptive and require examination in a larger sample with prospectively defined strata or a continuous moderation analysis.”

Page 17, lines 373–375:

“These findings should be interpreted with caution: the intervention conditions differed in musical material and duration, so the independent effects of the LLM-generated, culturally adapted suggestions could not be isolated.”

Comment 4.

Overall, the manuscript addresses a timely and interesting question, and the effort to integrate LLM-generated culturally adapted verbal suggestions into music therapy is commendable. Addressing the statistical and conceptual points raised above would meaningfully strengthen the rigour and credibility of the study, and the authors are encouraged to revise the manuscript with these considerations in mind.

Response

Finally, we would like to express our sincere gratitude to the editor and reviewer for their careful and patient identification of the study’s limitations and for their valuable recommendations. We particularly appreciate your recognition of our efforts to integrate LLM-generated, culturally adapted verbal suggestions into music therapy. After addressing the limitations of the present study, we intend to continue developing this line of research.

Attachment Submitted filename: Response to Editor and Reviewers.docx

# Editor report: Decision Letter 3

**DOI:** 10.1371/journal.pone.0360478.r007

**Authors:** Thiago P. Fernandes

10 Sep 2026

Integrating Large Language Model-Generated Culturally Adapted Verbal Suggestions into Music Therapy for University Students: A Pilot Randomized Exploratory Study

PONE-D-26-05582R3

Dear Dr. Li,

We’re pleased to inform you that your manuscript has been judged scientifically suitable for publication and will be formally accepted for publication once it meets all outstanding technical requirements.

Within one week, you’ll receive an e-mail detailing the required amendments. When these have been addressed, you’ll receive a formal acceptance letter and your manuscript will be scheduled for publication.

An invoice will be generated when your article is formally accepted. Please note, if your institution has a publishing partnership with PLOS and your article meets the relevant criteria, all or part of your publication costs will be covered. Please make sure your user information is up-to-date by logging into Editorial Manager at Editorial Manager® and clicking the ‘Update My Information' link at the top of the page. For questions related to billing, please contact billing support.

If your institution or institutions have a press office, please notify them about your upcoming paper to help maximize its impact. If they’ll be preparing press materials, please inform our press team as soon as possible -- no later than 48 hours after receiving the formal acceptance. Your manuscript will remain under strict press embargo until 2 pm Eastern Time on the date of publication. For more information, please contact onepress@plos.org.

Kind regards,

Thiago P. Fernandes, PhD

Academic Editor

PLOS One

Additional Editor Comments (optional):

Thank you for your thoughtful and careful edits. Wishing you success with the study.

Reviewers' comments:

# Editor report: Acceptance letter

**DOI:** 10.1371/journal.pone.0360478.r008

**Authors:** Thiago P. Fernandes

PONE-D-26-05582R3

PLOS One

Dear Dr. Li,

I'm pleased to inform you that your manuscript has been deemed suitable for publication in PLOS One. Congratulations! Your manuscript is now being handed over to our production team.

At this stage, our production department will prepare your paper for publication. This includes ensuring the following:

* All references, tables, and figures are properly cited

* All relevant supporting information is included in the manuscript submission,

* There are no issues that prevent the paper from being properly typeset

You will receive further instructions from the production team, including instructions on how to review your proof when it is ready. Please keep in mind that we are working through a large volume of accepted articles, so please give us a few days to review your paper and let you know the next and final steps.

Lastly, if your institution or institutions have a press office, please let them know about your upcoming paper now to help maximize its impact. If they'll be preparing press materials, please inform our press team within the next 48 hours. Your manuscript will remain under strict press embargo until 2 pm Eastern Time on the date of publication. For more information, please contact onepress@plos.org.

You will receive an invoice from PLOS for your publication fee after your manuscript has reached the completed accept phase. If you receive an email requesting payment before acceptance or for any other service, this may be a phishing scheme. Learn how to identify phishing emails and protect your accounts at https://explore.plos.org/phishing.

If we can help with anything else, please email us at customercare@plos.org.

Thank you for submitting your work to PLOS One and supporting open access.

Kind regards,

PLOS One Editorial Office Staff

on behalf of

Dr. Thiago P. Fernandes

Academic Editor

PLOS One
